# Indole Derivatives as New Structural Class of Potent and Antiproliferative Inhibitors of Monocarboxylate Transporter 1 (MCT1; SLC16A1)

**DOI:** 10.1021/acs.jmedchem.2c01612

**Published:** 2022-12-30

**Authors:** Sachin Puri, Katja Stefan, Sharuk L. Khan, Jens Pahnke, Sven Marcel Stefan, Kapil Juvale

**Affiliations:** † Shobhaben Pratapbhai Patel School of Pharmacy & Technology Management, SVKM’s NMIMS, V.L. Mehta Road, Vile Parle (W), Mumbai400056, India; ‡ Department of Pathology, Section of Neuropathology, Translational Neurodegeneration Research and Neuropathology Lab (www.pahnkelab.eu), University of Oslo and Oslo University Hospital, Sognsvannsveien 20, 0372Oslo, Norway; § Department of Pharmaceutical Chemistry, N.B.S. Institute of Pharmacy, Ausa413520, Maharashtra, India; ∥ Drug Development and Chemical Biology Lab, Lübeck Institute of Experimental Dermatology (LIED), University of Lübeck and University Medical Center Schleswig-Holstein, Ratzeburger Allee 160, 23538Lübeck, Germany; ⊥ Department of Pharmacology, Faculty of Medicine, University of Latvia, Jelgavas iela 4, 1004Ri̅ga, Latvia

## Abstract

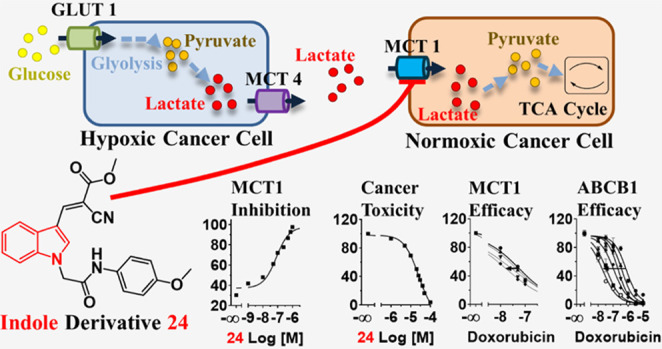

The solute carrier (SLC) monocarboxylate transporter 1 (MCT1; SLC16A1) represents a promising target for the treatment of cancer; however, the MCT1 modulator landscape is underexplored with only roughly 100 reported compounds. To expand the knowledge about MCT1 modulation, we synthesized a library of 16 indole-based molecules and subjected these to a comprehensive biological assessment platform. All compounds showed functional inhibitory activities against MCT1 at low nanomolar concentrations and great antiproliferative activities against the MCT1-expressing cancer cell lines A-549 and MCF-7, while the compounds were selective over MCT4 (SLC16A4). Lead compound **24** demonstrated a greater potency than the reference compound, and molecular docking revealed strong binding affinities to MCT1. Compound **24** led to cancer cell cycle arrest as well as apoptosis, and it showed to sensitize these cancer cells toward an antineoplastic agent. Strikingly, compound **24** had also significant inhibitory power against the multidrug transporter ABCB1 and showed to reverse ABCB1-mediated multidrug resistance (MDR).

## Introduction

Cancer is a leading cause of death in over 70% of the countries worldwide and accounts for 30% of all premature deaths of all noncommunicable diseases.^1^ Chemotherapy is the most important noninvasive treatment available. However, first- and even second-line anticancer drugs become often ineffective^2^ due to the occurrence of multidrug resistance (MDR) of cancer cells, by either mutations/changed drug targets^3^ or reduced drug uptake/upregulation of efflux transporters,^2,4,5^ amongst other reasons. Although tremendous advances have been made within the last two decades by the discovery of novel anticancer drug targets,^4,6−8^ the frequent occurrence of MDR forces research to focus on new pharmacological targets for the exploration of novel cancer drug targets of the future.

Cancer cells go through a process of metabolic reprogramming to contest changes in the metabolism such as hypoxia due to poor vascularization or increased glucose consumption to support enhanced cell growth.^9^ The tumor microenvironment and nutrition status have been acknowledged as driving factors in tumor growth, prognosis, and survival rate.^10,11^ Particularly, cancer cells within solid tumors are prone to hypoxia, leading to an overexpression of hypoxia-inducible factor-1 (HIF-1), triggering glucose transporter 1 (GLUT1) expression to increase glucose uptake.^9,11,12^ This changes the basic energy supply from mitochondrial oxidative phosphorylation (as in normoxic cells) to glycolytic metabolism.^9,11,13^ Thus, hypoxic cells convert glucose to pyruvate and lactate, and abundant lactate is extruded into the tumor microenvironment by monocarboxylate transporter 4 (MCT4). Strikingly, this lactate becomes imported by MCT1 and converted into pyruvate feeding the tricarboxylic acid (TCA) cycle^9,13^ and, thus, aerobic energy production. MCT1 overexpression is found in many cancer cells,^10,13−16^ and its support of cancer migration, invasion, and metastasis^13^ has been recognized. Hence, MCT1 represents a promising drug target for the development of novel anticancer agents.

MCT1 inhibition prevents lactate and pyruvate influx into aerobic cells, which forces the cells to use glucose for energy supply. This influx inhibition disrupts the symbiosis between aerobic (MCT1-expressing) and anaerobic (MCT4-expressing) cells in the tumor microenvironment by glucose deprivation, first in hypoxic cells, and eventually, normoxic cells, leading to apoptosis, finally increasing the susceptibility of the tumor cells to first- and second-line antineoplastic agents.^17^

**Figure 1:**
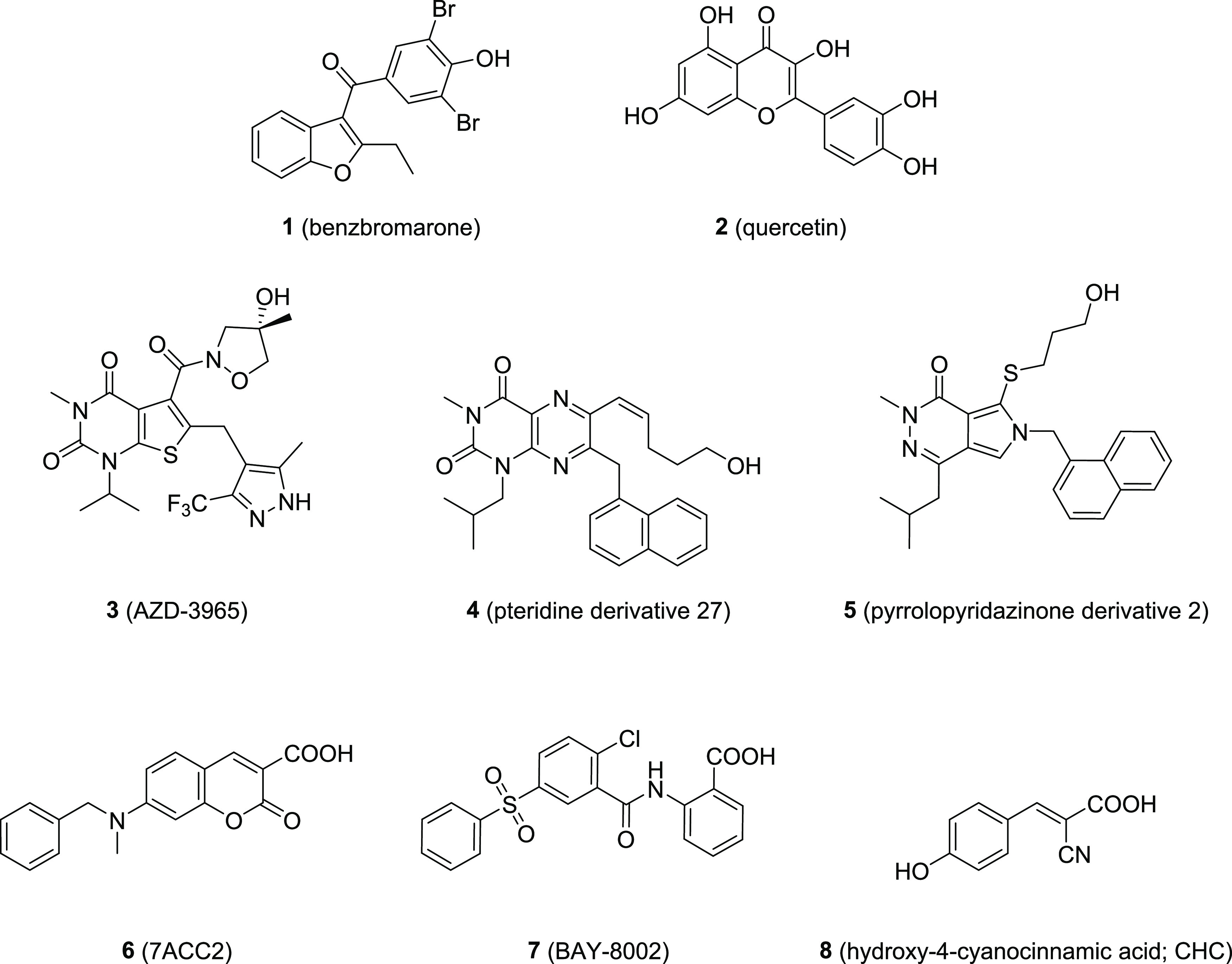
Molecular formulae of known drugs and druglike compounds inhibiting MCT1.

The number of MCT1-targeting agents is rather slim, and their structural diversity is highly limited.^18^ Until today, only roughly 100 compounds are known to interact with MCT1.^18−31^ Certain drugs and druglike compounds have been reported to inhibit MCT1,^26,30,31^ such as benzbromarone (**1**)^31^ and quercetin (**2**),^31^ however, with rather low potency. Amongst the high-throughput screening-(HTS)- and organic synthesis-derived compounds, only a few structurally distinctive compound classes can be differentiated: (i) thieno[2,3-*d*]pyrimidin-2,4-diones,^18,27−29^ [*e.g.*, AZD-3965 (**3**)^32^], pteridine derivatives^18,19,33^ (*e.g.*, compound **4**(33)), pyrrolo[3,4-*d*]pyridazinones^18,23,29^ (*e.g.*, compound **5**(29)), coumarin derivatives^18,19,25^ [*e.g.*, 7ACC2 (**6**)^25^], the sulfonyldibenzene BAY-8002 (**7**),^18,19,34^ as well as cinnamic acid derivatives^18,21,22,24,31^ [*e.g.*, hydroxy-4-cyanocinnamic acid (CHC); **8**].^18,31^Figure 1 shows the molecular structures of compounds **1**–**8**.

**Figure 2:**
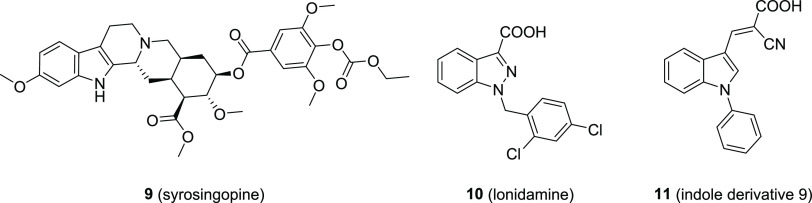
Indole and indole-like derivatives reported as MCT1 inhibitors. Left: the reserpine analog syrosingopine (**9**) as reported by Buyse et al. in 2022;^35^ middle: the antineoplastic agent lonidamide (**10**) as reported by Nancolas et al. in 2016;^37^ right: the indole derivative **9** as reported by Samuel in 2016.^38^

Recently, very few MCT1 inhibitors were reported with indole or indole-like scaffolds,^13,35−37^ namely, syrosingopine (**9**),^35^ lonidamine (**10**),^13,36,37^ and compound **11**.^38^Figure 2 shows the mentioned molecules.

To explore indoles as potential novel molecular–structural class of MCT1 inhibitors, we extended the structural diversity of indoles by synthesizing 16 derivatives including their comprehensive *in vitro* assessment against MCT1-mediated transport, cancer cell viability, and cancer cell cycle, but also against selected drug transporters. We were able to elucidate important structure–activity relationships (SARs) and discovered potent and antiproliferative agents that may represent the lead molecules for further clinical evaluations.

## Results

### Chemistry

**Scheme 1:**
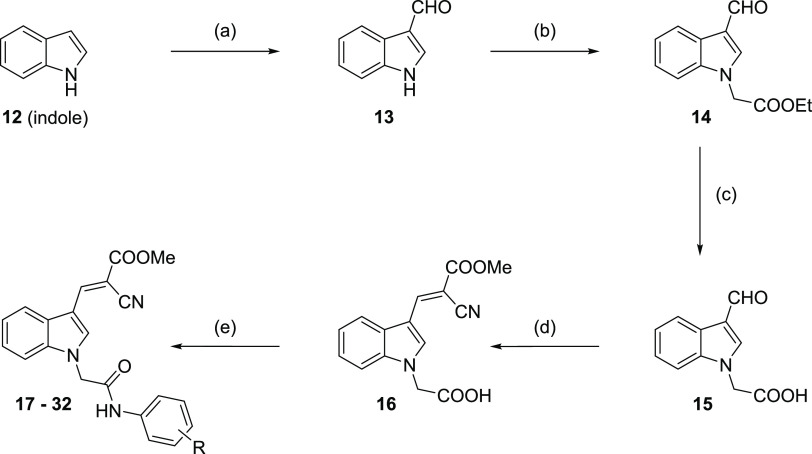
General Synthesis of Target Compounds **17**–**32** Reagents and conditions: (a) compound **12**, POCl_3_, *N*,*N*-dimethylformamide (DMF), 0 °C, overnight; (b) compound **13**, ethyl bromoacetate, Cs_2_CO_3_, DMF, 0 °C, 1 h; (c) compound **14**, NaOH, ethanol, reflux, 1 h; (d) compound **15**, methyl cyanoacetate, piperidine, ethanol, room temperature, overnight; (e) compound **16** with either (i) respective amine, pyridine, POCl_3_, dichloromethane (DCM), 0 °C–room temperature; 6–8 h or (ii) respective amine, EDC·HCl, HOBt, *N*,*N*-diisopropylethylamine (DIPEA), DMF, 0 °C–room temperature, 6–8 h.

The 2-cyano-3-(1-(2-oxo-2-(phenylamino)ethyl)-1*H*-indol-3-yl) acrylate lead structure was synthesized using indole (**12**) as the starting material. A Vilsmeier–Haack reaction provided the formylated 1*H*-indole-3-carbaldehyde (**13**), which was further reacted with ethyl bromoacetate to obtain ethyl 2-(3-formyl-1*H*-indol-1-yl) acetate (**14**). Hydrolyzation with sodium hydroxide yielded product 2-(3-formyl-1*H*-indol-1-yl) acetic acid (**15**). A Knoevenagel condensation with methyl cyanoacetate revealed 2-(3-(2-cyano-3-methoxy-3-oxoprop-1-en-1-yl)-1*H*-indol-1-yl) acetic acid (**16**). The respective amine was coupled to intermediate **16** to result in target compounds **17**–**32**. Scheme 1 visualizes the multistep synthesis of compounds **17**–**32**.

### Biological Evaluation

#### Inhibitory Activity against MCT1

The inhibitory activity of indole derivatives against MCT1 was determined by applying a functional 3-bromopyruvate (3-BP) cell viability assay, as described in the literature^20,39^ with minor modifications. In short, MCT1-expressing A-549 cells^14,15^ were exposed to the MCT1 substrate 3-BP, which becomes influxed by MCT1. 3-BP is toxic, leading to decreased cell viability. Functional inhibition of MCT1 results in reduced intracellular 3-BP levels and, thus, enhanced cell viability compared to no inhibition. The half-maximal growth inhibition (GI_50_) value of 3-BP was determined to be 25.0 ± 0.4 μM. As 50 μM 3-BP inhibited A-549 cell growth by ≥90% (GI_90_ = 49.8 ± 0.6 μM), this concentration was used to assess the inhibitory activity of indole derivatives. Table 1 provides the half-maximal inhibition concentration (IC_50_) values of compounds **17**–**32**.

**Table 1:**
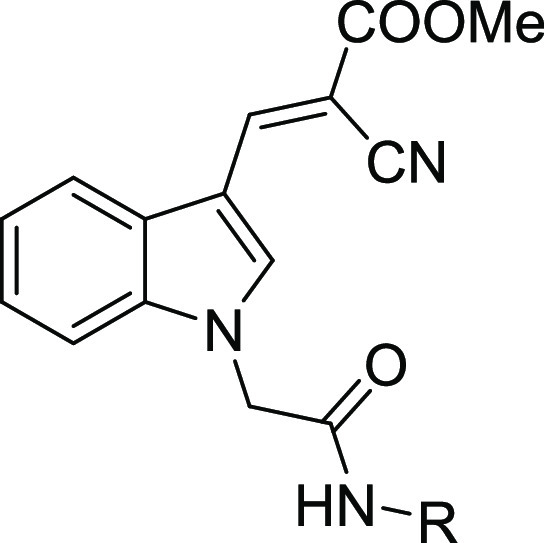
Inhibitory Activity of Compounds **17**–**32** Compared to MCT1 Inhibition by Reference Inhibitor **8** as Determined in a Functional 3-BP Cell Viability Assay as Described Earlier^20,39^ Using MCT1-Expressing A-549 Cells^14,15^^,^^a^

		MCT1 transport inhibition
no.	R	IC_50_ ± SEM [nM]
**8**		87.2 ± 3.5
**17**	phenyl	314 ± 47
**18**	1-naphthyl	193 ± 10
**19**	2-naphthyl	109 ± 3
**20**	2-methylphenyl	105 ± 6
**21**	4-methylphenyl	112 ± 10
**22**	2-methoxyphenyl	135 ± 2
**23**	3-methoxyphenyl	105 ± 9
**24**	4-methoxyphenyl	81.0 ± 4
**25**	4-fluorophenyl	793 ± 138
**26**	4-chlorophenyl	196 ± 5
**27**	2,6-dichlorophenyl	109 ± 2
**28**	4-bromophenyl	287 ± 47
**29**	2-pyridyl	198 ± 4
**30**	5-methyl-2-pyridyl	82.3 ± 4.4
**31**	5-bromo-2-pyridyl	214 ± 32
**32**	3-pyridyl	109 ± 3

a Data are expressed as IC_50_ ± standard error of the mean (SEM) of at least three independent experiments.

**Figure 3:**
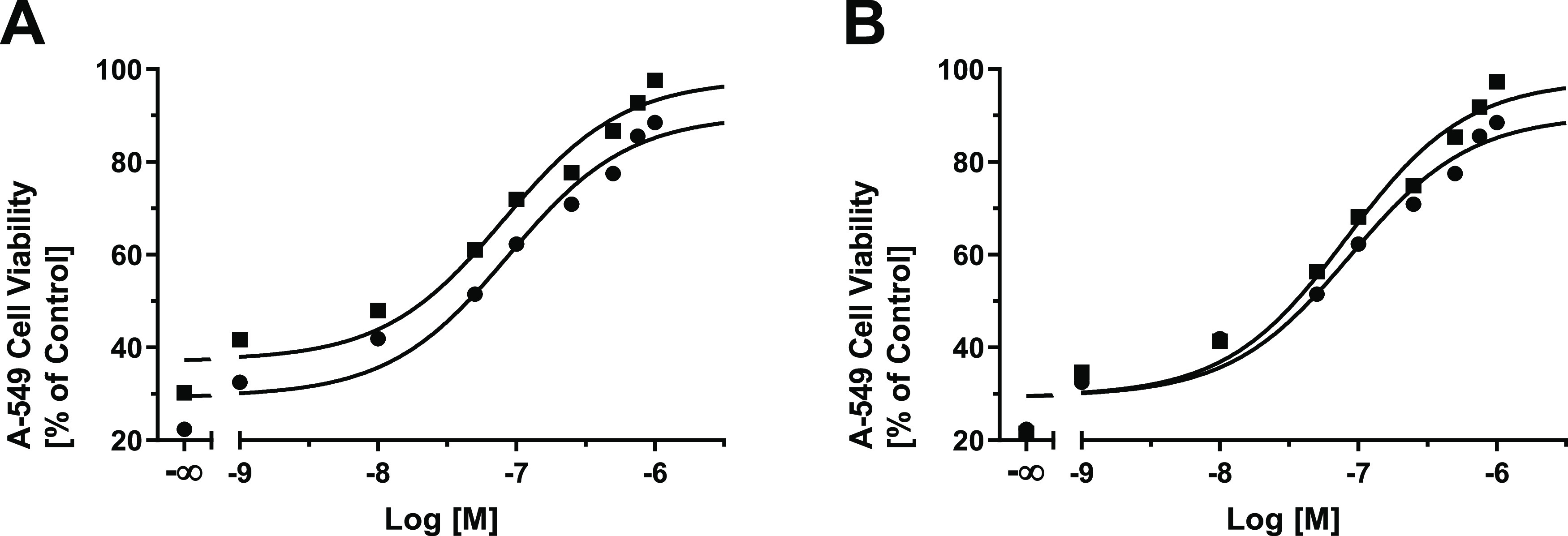
Concentration-effect curves of the most potent MCT1 inhibitors reported in this work, compound **24** (A; solid squares; IC_50_ = 81.0 nM) and compound **30** (B; solid squares, IC_50_ = 82.3 nM) in comparison to reference inhibitor **8** (A–B; solid circles, IC_50_ = 87.2 nM) in the functional 3-PB cell viability assay^20,39^ applying MCT1-expressing A-549 cells.^14,15^ 100% cell viability was defined by no supplementation of 3-BP, while 0% represents the effect of 3-BP alone without the addition of the test compounds. Mean ± SEM values of at least three independent experiments are shown.

All indole derivatives displayed an inhibition of MCT1-mediated transport in submicromolar concentration ranges. The plain lead compound **17** with a phenyl substitution at the amide possessed an IC_50_ value of 314 nM. Only compound **25** with a 4-flourine substitution showed weaker MCT1 inhibition (IC_50_ = 793 nM). Compound **25** represents an exception considering the SAR, as both para-substitutions in general (**19**, **21**, **24, 26**, **28**) and *para*-halogen substitutions in particular (**26**, **28**) led to a strong increase of inhibitory power. Particularly, compound **24** with its 4-methoxy substitution showed the highest MCT1 transport inhibition with an IC_50_ value of 81.0 nM, exceeding the inhibitory power of the reference MCT1 inhibitor **8**. Pyridyl residues (**29**–**32**) were also generally accepted with inhibitory activities below the IC_50_ of the plain lead compound **17**. Especially compound **30** with its 5-methyl-substituted 2-pyridyl residue showed similar inhibitory power against MCT1 as compound **24** and superior activity compared to the reference MCT1 inhibitor **8**. Figure 3 provides the concentration-effect curves of the most potent MCT1 inhibitors of the indole series, **24** (A) and **30** (B), compared to the reference MCT1 inhibitor **8**.

#### Inhibitory Power against MCT4

MCT4 is a key protein in the metabolic reprogramming of hypoxic tumor cells. Thus, we investigated the MCT1 inhibitors for their potential activity against MCT4, applying a pH-sensitive fluorescence assay as described in the literature^35,40^ with minor modifications. In short, functional MCT4 inhibition prevents the extrusion of acidic lactate, which acidifies the cytosol of the MCT4-expressing cancer cell line MDA-MB-231.^22,41,42^ The degree of inhibition is reflected in the magnitude of pH reduction. However, none of the 16 evaluated indole derivatives showed a pH reduction in MCT4-expressing MDA-MB-231^22,41,42^ cells (data not shown).

#### Antiproliferative Activity against MCT1-Expressing Cancer Cell Lines

MCT1-expressing cells have an extraordinary energy demand and rely on pyruvate metabolism, through either lactate conversion or pyruvate consumption. Inhibition of MCT1 blocks lactate and pyruvate influx into cancer cells, which prevents their processing in the TCA cycle. Over time, the viability of cells exposed to an MCT1 inhibitor is impaired due to pyruvate deprivation, which is compensated by upregulating glucose metabolism—leading to an overconsumption of glucose. To confirm the antiproliferative nature of the indole derivatives, we investigated the compounds toward the MCT1-expressing cell lines A-549^14,15^ and MCF-7.^16^ Cancer cell viability was assessed *via* a 3-(4,5-dimethylthiazol-2-yl)-2,5-diphenyltetrazolium bromide (MTT) assay, as reported earlier.^43^

**Figure 4:**
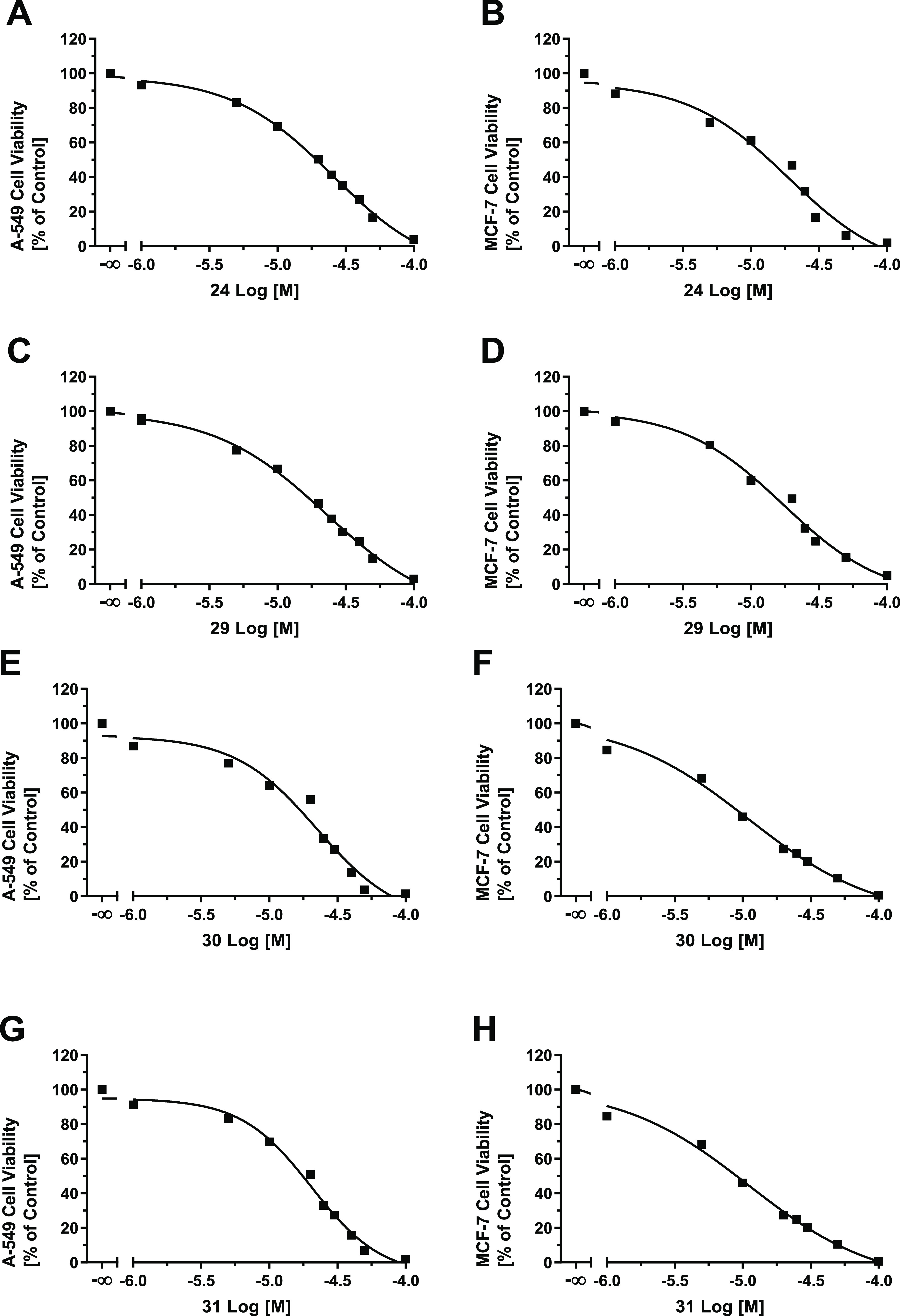
Concentration-effect curves of the most potent lead MCT1 inhibitor **24** (A, B), as well as the 2-pyridyl-derivatives **29** (C, D), **30** (E, F), and **31** (G, H) obtained from an MTT-based viability assay^43^ using MCT1-expressing^14−16^ A-549 (A, C, E, G) and MCF-7^16^ (B, D, F, H) cancer cells. DMSO and culture medium were used to define 0 and 100%, respectively. Mean ± SEM values of at least three independent experiments are shown.

As can be seen from Table 2, the entire compound class of indoles bears considerable cytotoxicity toward MCT1-expressing cancer cell lines, being more toxic than the reference MCT1 inhibitor **8**. Interestingly, the 2-pyridyl derivatives **29**–**31** including the second most potent lead MCT1 inhibitor **30** had the greatest effect on cancer cell viability. Figure 4 shows the concentration-effect curves of the most potent MCT1 inhibitor, compound **24** (A–B), as well as the 2-pyridyl-derivatives **29** (C–D), **30** (E–F), and **31** (G–H) using A-549^14,15^ (A, C, E, G) and MCF-7^16^ (B, D, F, H) cancer cells.

**Table 2:** Results of the MTT-Based Cell Viability Assay^43^ Assessing Compounds **8** and **17**–**32** for Their Toxicity against MCT1-Expressing A-549^14,15^ and MCF-7^16^ Cancer Cells^a^

no.	A-549 cancer cell toxicity GI_50_ ± SEM [μM]	MCF-7 cancer cell toxicity GI_50_ ± SEM [μM]
**8**	52.7 ± 0.2	108 ± 3
**17**	29.2 ± 0.2	15.4 ± 0.3
**18**	21.5 ± 0.4	15.4 ± 0.5
**19**	26.9 ± 0.3	15.5 ± 1.3
**20**	28.3 ± 0.1	15.3 ± 0.0
**21**	14.0 ± 0.5	13.9 ± 0.1
**22**	26.0 ± 0.3	19.1 ± 0.5
**23**	25.5 ± 0.5	14.7 ± 0.2
**24**	20.0 ± 0.6	15.1 ± 0.4
**25**	22.0 ± 0.6	15.1 ± 0.3
**26**	21.0 ± 0.4	10.7 ± 0.4
**27**	25.0 ± 0.5	17.3 ± 0.6
**28**	19.2 ± 0.2	9.99 ± 0.46
**29**	17.1 ± 0.4	4.32 ± 0.14
**30**	19.3 ± 0.3	9.12 ± 0.22
**31**	18.8 ± 0.2	3.13 ± 0.23
**32**	19.2 ± 0.4	11.1 ± 0.2

a Dimethyl sulfoxide (DMSO) and culture medium were used to define 0 and 100%, respectively. Half-maximal growth inhibition (GI_50_) values ± SEM of at least three independent experiments are shown.

**Figure 5:**
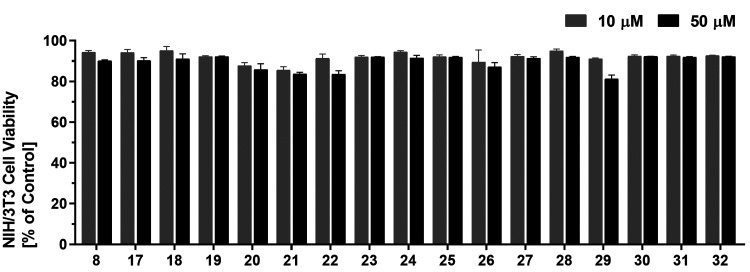
Screening of indole derivatives **8** and **17**−**32** for their cell toxicity against non-MCT1-expressing, noncancerous murine embryonic fibroblast NIH/3T3 cells at 10 μM (gray) and 50 μM (black) compound concentrations applying an MTT cell viability assay, as described earlier.^43^ DMSO and culture medium were used to define 0 and 100%, respectively. Mean ± SEM values of at least three independent experiments are shown.

In the next step, the cell toxicity^43^ of the compounds was analyzed against the non-MCT1-expressing, noncancerous murine embryonic fibroblast cell line NIH/3T3 to analyze whether the observed effects relate to malignant MCT1-(over)expression. Strikingly, none of the indole derivatives had a considerable impact on the cell viability of NIH/3T3 cells up to a concentration of 50 μM (Figure 5). This finding led us to the conclusion that the observed effects are due to the selective inhibition of MCT1, which is expressed in A-549^14,15^ and MCF-7^16^ cancer cell lines.

**Figure 6:**
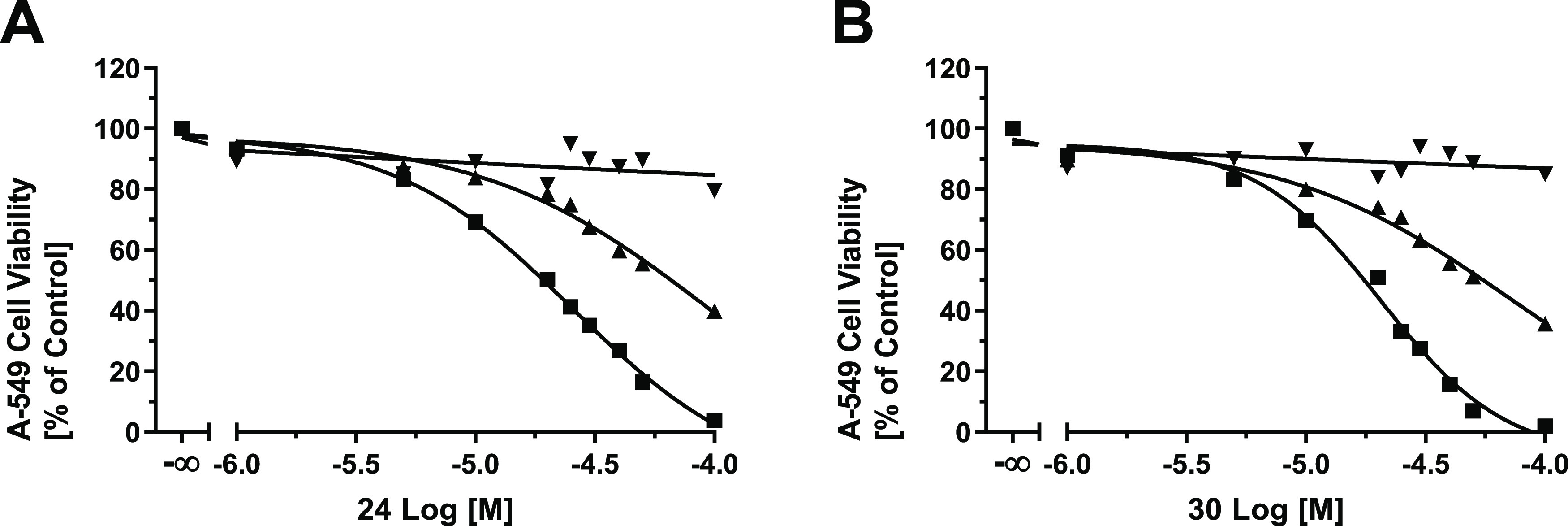
MTT-based cell viability assays of compounds **24** (A) and **30** (B) either without (solid circles) or with 25% (solid up triangles) and 50% (solid down triangles) supplemented glucose every 12 h over the time course of 72 h. DMSO and culture medium were used to define 0 and 100%, respectively. Mean ± SEM values of at least three independent experiments are shown.

To prove that the cell death of MCT1-expressing cancer cells results from glucose deprivation, we conducted additional MTT-based cell viability experiments with compounds **24** and **30** supplementing glucose (25 and 50% of the original glucose concentration of 4.5 g/L every 12 h) over a time span of 72 h. Strikingly, we could, for the first time, prove that the supplementation of glucose prevents cell toxicity of the MCT1 inhibitors **24** (Figure 6A) and **30** (Figure 6B), thus confirming that glucose deprivation is the main cause of cell death mediated by MCT1 inhibitors.

#### Efficacy of Compounds **24** and **30** against MCT1-Expressing Cells

**Figure 7:**
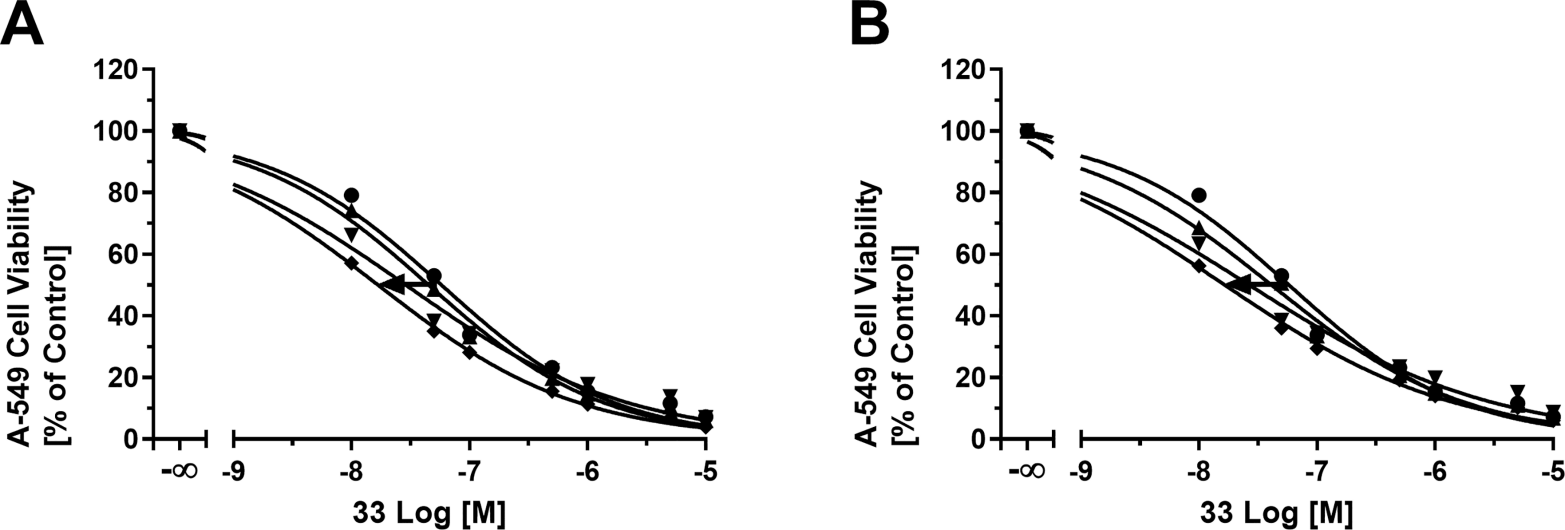
Concentration-effect curves of the antineoplastic agent **33** without (solid circles) and with 50 nM (solid up triangles), 100 nM (solid down triangles), and 500 nM (solid diamonds) of either compound **24** (A) or **30** (B) as determined in an MTT-based cell viability assay^43^ using MCT1-expressing A-549 cells.^14,15^ Mean ± SEM of at least three independent experiments is shown.

MCT1 inhibition anticipates challenging cancer cell energy supply and, thus, increases the efficacy of anticancer agents. To prove this hypothesis, we investigated the effect of the antineoplastic agent doxorubicin (**33**) on MCT1-expressing A-549^14,15^ cells in combination with either compound **24** or **30** (50, 100, or 500 nM) in a time frame of 72 h, applying an MTT-based efficacy assay.^43^Figure 7 shows the resultant concentration-effect curves of **33** without and with supplementation of compounds **24** (A) or **30** (B). In both cases, the compounds sensitized the cells against **33** by factors of 2.5 and 2.6, respectively, as indicated by the shift of the concentration-effect curve to the left, demonstrating their capability to increase susceptibility toward antineoplastic agents. Table 3 provides the GI_50_ values of **33** either without or with increasing concentrations of either compound **24** or **30**.

**Table 3:** Results of the MTT-Based Efficacy Assay^43^ Using the Antineoplastic Agent **33** Either without or in Combination with Either Compound **24** or **30** at Concentrations of 50 nM, 100 nM, or 150 nM Using MCT1-Expressing A-549 Cells^14,15^^,^^a^

	A-549	A-549	A-549	A-549
	cell toxicity **33** +	cell toxicity **33** +	cell toxicity **33** +	cell toxicity **33** +
	0 nM test cmpd.	50 nM test cmpd.	100 nM test cmpd.	500 nM test cmpd.
no.	GI_50_ ± SEM [nM]	GI_50_ ± SEM [nM]	GI_50_ ± SEM [nM]	GI_50_ ± SEM [nM]
**24**	47.6 ± 0.7	40.0 ± 0.2	27.7 ± 0.4	19.4 ± 0.3
**30**	47.6 ± 0.7	38.6 ± 0.5	25.4 ± 0.4	18.6 ± 0.4

a DMSO and culture medium were used to define 0 and 100%, respectively. GI_50_ values ± SEM of at least three independent experiments are shown.

#### Cell Cycle Distribution Analysis of Compound **24**

**Figure 8:**
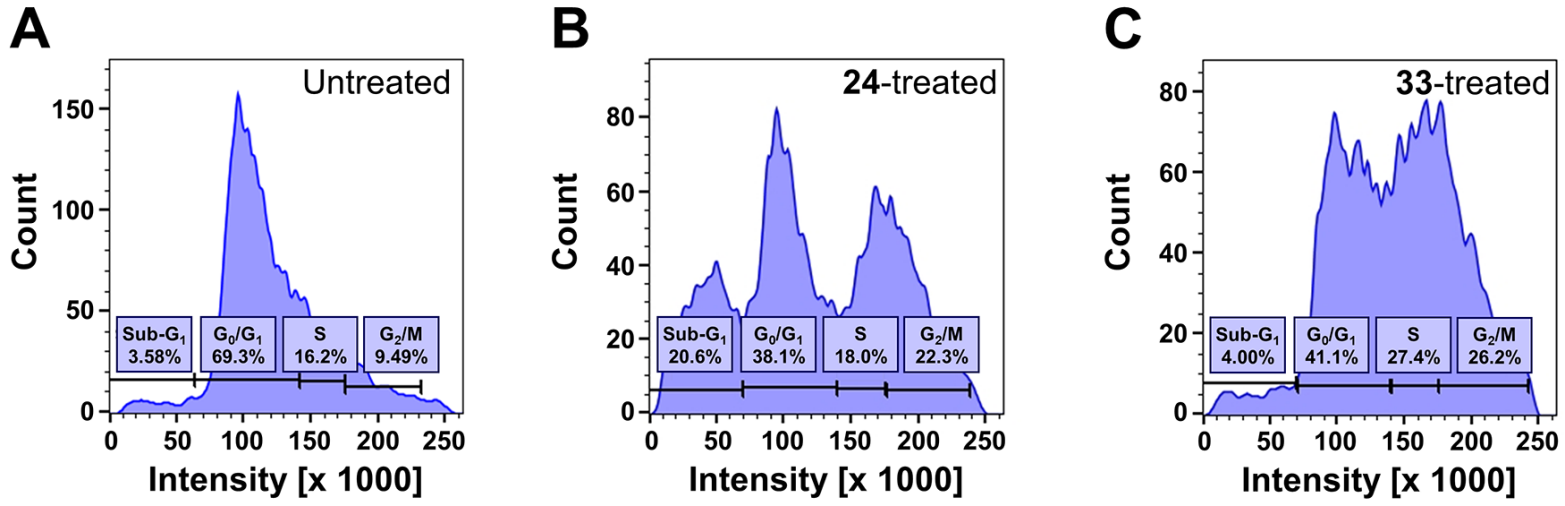
Distribution of cell cycle phases of untreated A-549 cancer cells (A) as well as A-549 cells treated with **24** (5 μM; B) or **33** (5 μM; C). Representative experiments out of three independent experiments are shown.

To prove that indole derivatives impair cancer cell proliferation and to confirm their antiproliferative nature, we conducted a cell cycle distribution analysis *via* a propidium iodide (PI) flow cytometry assay, as reported earlier.^44−46^ A-549 cells were exposed to the most potent MCT1 inhibitor, **24** (5 μM), and the distribution of the cells in the cell cycle phases sub-*G*_1_, *G*_0_/*G*_1_, *S*, and *G*_2_/*M* was calculated compared to untreated A-549 cells. The antineoplastic agent **33** (5 μM) was used as a reference. Figure 8 shows the histograms for untreated (A), **24**-treated (B), and **33**-treated cells (C). Compound **24** caused a disruption of the cell cycle of A-549 cancer cells, indicated by a shift from the predominant *G*_0_/*G*_1_ phase (Figure 8A) to the apoptotic sub-*G*_1_ phase as well as the *G*_2_/M phase (Figure 8B). A similar *G*_2_/M arrest could be observed for reference compound **33** (Figure 8C); however, MCT1 inhibitor **24** led to a larger fraction of cells into the sub-*G*_1_ phase, indicating induced apoptosis. This experiment discovered that compound **24** inhibits cell proliferation, causing cell cycle arrest and, eventually, apoptosis. To confirm our findings, we conducted an apoptosis assay using compound **24**.

#### Apoptosis Assay

**Figure 9:**
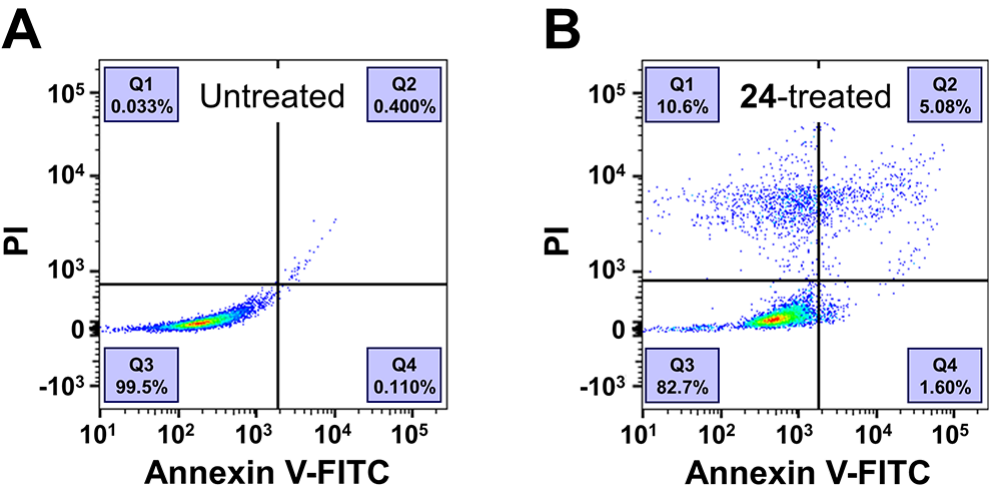
Distribution of apoptotic A-549 cancer cells when untreated (A) or exposed to compound **24** (B). Representative experiments out of three independent experiments are shown.

As compound **24** increased the sub-*G*_1_ cell cycle fraction of A-549 cells, we investigated its capability to induce apoptosis in a PI/Annexin V-FITC staining assay, as reported earlier.^44,46−51^ In short, cells that were stained with PI only were considered necrotic cells (Q1), while cells stained with both PI and Annexin V were late apoptotic (Q2), and cells stained with Annexin V only were early apoptotic (Q4). Cells negative on both PI and Annexin V were considered living cells (Q3). Compound **24** (5 μM) increased the percentage of total apoptotic cells by a factor of 13 from 0.51 to 6.68% when compared to the untreated A-549 cells, indicating that **24** exhibited cytotoxicity against the A-549 cancer cell line *via* inducing apoptosis. Figure 9 shows the flow cytometry scatter plot of untreated (A) compared to that of **24**-treated A-549 cells (B).

#### Inhibitory Activity against Multidrug Transporters ABCB1, ABCC1, and ABCG2

**Figure 10:**
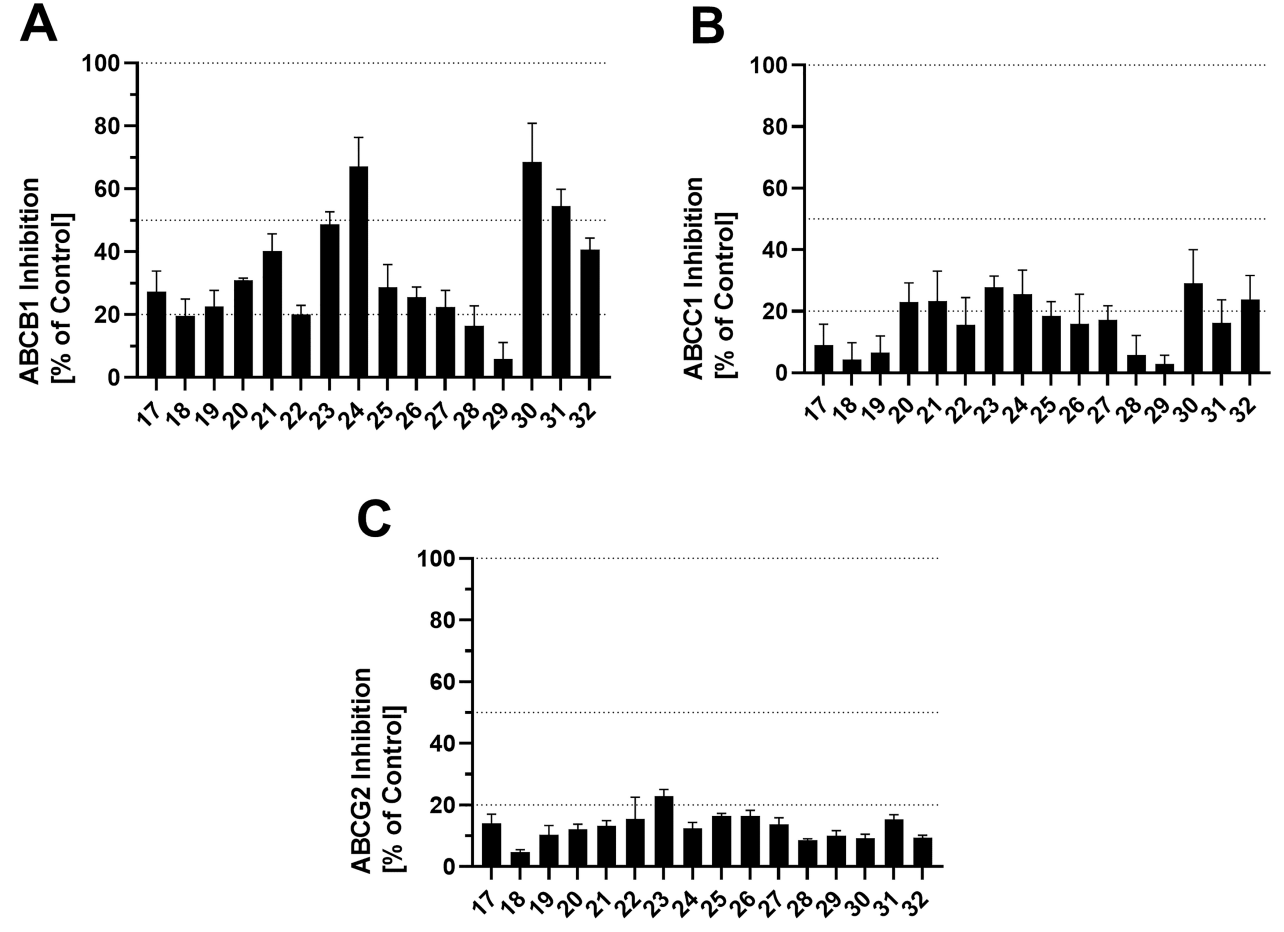
Screening of compounds **17**–**32** against the ABC transporters ABCB1 (A), ABCC1 (B), and ABCG2 (C) applying calcein AM (ABCB1, A),^2,52−59^ daunorubicin (ABCC1, B),^52,58−60^ and pheophorbide A (ABCG2, C)^2,52−60^ assays using ABCB1-expressing A2780/ADR (A), ABCC1-expressing H69AR (B), and ABCG2-expressing MDCK II BCRP (C) cells, respectively. Data were normalized by defining the effect of 10 μM cyclosporine A (**34**, ABCB1, A), compound **35** (ABCC1, B),^58,61^ and Ko143 (**36**, ABCG2, C) as a positive control (100%) and buffer medium as negative control (0%). Mean ± SEM values of at least three independent experiments are shown.

Multidrug transporters of the ATP-binding cassette (ABC) superfamily play a major role in MDR and the distribution of antineoplastic agents.^2,4,5^ To provide a detailed picture of the pharmacological profile of the indole derivatives, we investigated their modulatory nature on the most prominent ABC transporters ABCB1 (P-glycoprotein, P-gp), ABCC1, (multidrug resistance-associated protein 1, MRP1), and ABCG2 (breast cancer resistance protein, BCRP). Here, we conducted calcein AM (ABCB1),^2,52−59^ daunorubicin (ABCC1),^52,58−60^ and pheophorbide A (ABCG2)^2,52−60^ assays using ABCB1-expressing A2780/ADR, ABCC1-expressing H69AR, and ABCG2-expressing MDCK II BCRP cells, respectively. Figure 10 shows the screening results of the compounds at a concentration of 10 μM against ABCB1 (A), ABCC1 (B), and ABCG2 (C), and Table 4 provides the IC_50_ values of selected compounds.

**Table 4:** Inhibitory Potencies of Selected Indole Derivatives against the ABC Transporters ABCB1, ABCC1, and ABCG2 Obtained in Calcein AM (ABCB1),^2,52−59^ Daunorubicin (ABCB1^52,60^ and ABCC1^52,58−60^), and Pheophorbide A (ABCG2)^2,52−60^ Assays Using ABCB1-Expressing A2780/ADR (A), ABCC1-Expressing H69AR (B), and ABCG2-Expressing MDCK II BCRP (C) Cells, Respectively^a^

no.	ABCB1 calcein AMIC_50_ ± SEM [μM]	ABCB1 daunorubicinIC_50_ ± SEM [μM]	ABCC1 daunorubicinIC_50_ ± SEM [μM]	ABCG2 pheophorbide AIC_50_ ± SEM [μM]
**22**	24.3 ± 6.4^b^	n.t.	17.3 ± 2.2^i^	99.5 ± 24.5^j^
**23**	5.32 ± 1.67^b^	n.t.	19.1 ± 3.4^i^	43.5 ± 7.5^j^
**24**	0.983 ± 0.135^c^	1.49 ± 0.05^f^	n.t.	n.t.
**30**	1.51 ± 0.08^d^	2.77 ± 0.02^g^	n.t.	n.t.
**31**	1.40 ± 0.11^e^	1.19 ± 0.12^h^	n.t.	n.t.
**34**	0.960 ± 0.018	0.851 ± 0.003		
**35**			0.215 ± 0.033	
**36**				0.161 ± 0.011

a Mean ± SEM values of at least three independent experiments are shown.

b Constrained to the reference ABCB1 inhibitor **34**.

c *I*_max_ = 66.8 ± 3.3%.

d *I*_max_ = 61.1 ± 2.1%

e *I*_max_ = 47.9 ± 4.9%.

f *I*_max_ = 84.0 ± 2.5%.

g *I*_max_ = 93.3 ± 2.6%.

h *I*_max_ = 39.0 ± 2.8%.

i Constrained to the reference ABCC1 inhibitor **35**.^58,61^

j Constrained to the reference ABCG2 inhibitor **36**; n.t., not tested due to lack of inhibitory activity in the initial screening.

**Figure 11:**
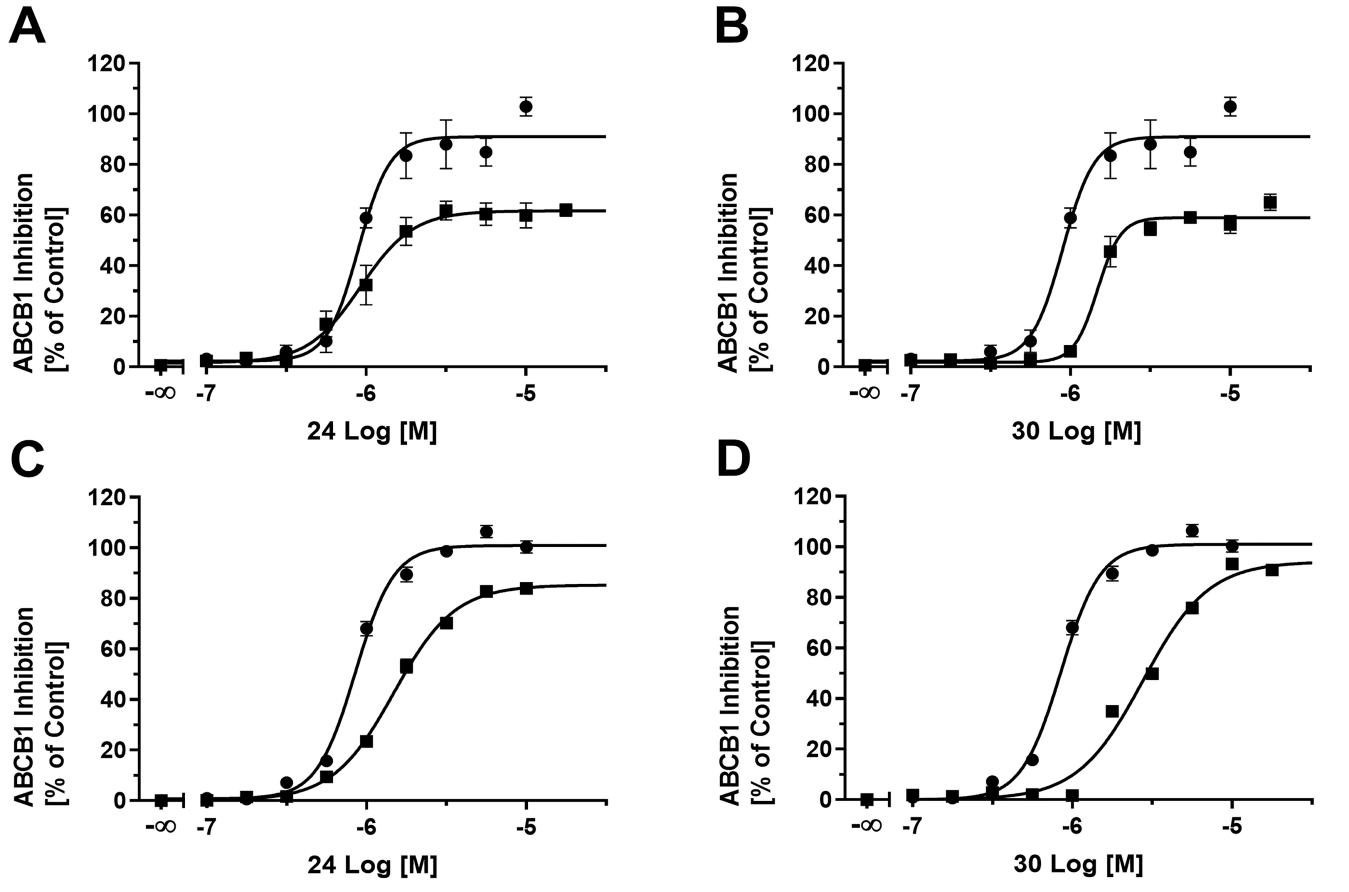
Concentration-effect curves of compounds **24** (A, C; solid squares) and **30** (B, D; solid squares) in comparison to reference inhibitor **34** (A–D; solid circles) applying calcein AM (A, B)^2,52−59^ and daunorubicin (C, D)^52,60^ assays using ABCB1-expressing A2780/ADR cells. Mean ± SEM values of at least three independent experiments are shown.

The lead MCT1 inhibitors **24** and **30**, as well as the pyridine derivative **31**, reached inhibition values of over 50% against ABCB1, while all compounds showed generally low biological activities against ABCC1 and ABCG2. Thus, lead compounds **24** and **30** are dual MCT1/ABCB1 inhibitors. Interestingly, the concentration-effect curves of compounds **24** (Figure 11A), **30** (Figure 11B), and **31** formed a plateau below 100%, as indicated by reference ABCB1 inhibitor cyclosporine A (**34**). The maximal inhibition (*I*_max_) of compounds **24** and **30** reached 66.8 and 61.1%, respectively, with IC_50_ values of 0.983 and 1.51 μM, respectively. This effect of apparent partial inhibition has frequently been observed in independent studies.^2,58,62−70^ It could be explained by impaired solubility or cell toxicity of the compounds. As this effect of partial inhibition frequently occurs in calcein AM assays,^57,58,63,66^ we verified our results in terms of the IC_50_ values in a daunorubicin assay, as reported earlier.^52,60^ Strikingly, the lead compounds **24** (Figure 11C) and **30** (Figure 11D) had inhibitory potencies of 1.49 and 2.77 μM, respectively, in the daunorubicin assay while having a similar *I*_max_ compared to reference ABCB1 inhibitor **34**.

Compounds **22**–**23**, on the other hand, showed inhibitory activities against all three evaluated ABC transporters (IC_50_ + SEM ≥ 20%) and, thus, qualify as the so-called “focused pan-ABC transporter inhibitors”.^71^ Multitarget agents with these polypharmacological profiles have been of increasing interest within recent years,^52−54,60,72−74^ and the herein discovered compounds **22**–**23** build a good starting point for the development of novel multitarget agents stretching from ABC to SLC transporters.

#### Efficacy of Compounds **24** and **30** against ABCB1-Expressing Cells

**Figure 12:**
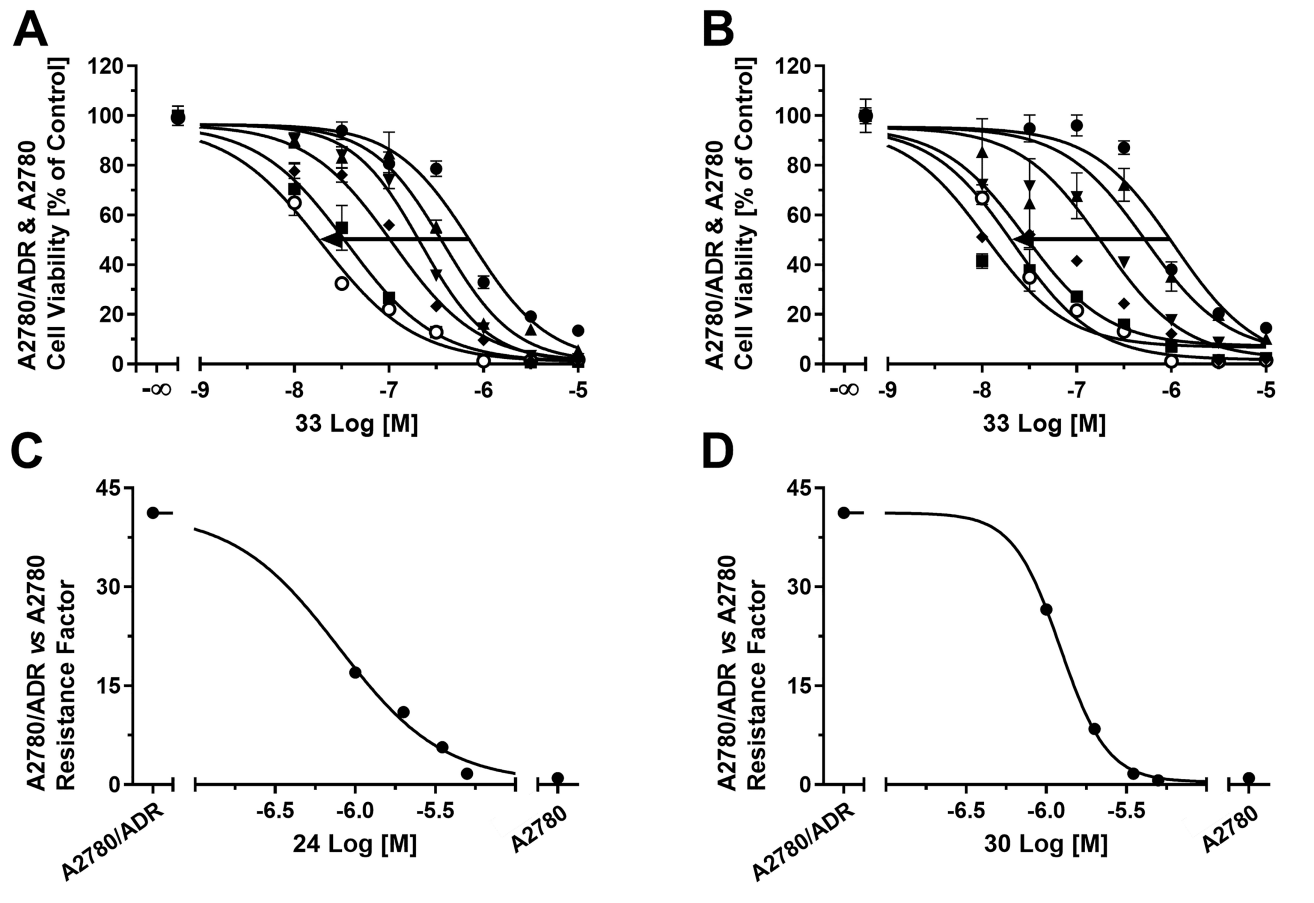
Concentration-effect curves of the antineoplastic agent **33** without (solid circles) and with 1.0 μM (solid up triangles), 2.0 μM (solid down triangles), 3.5 μM (solid diamonds), and 5.0 μM (solid squares) of either compound **24** (A) or **30** (B), as determined in an MTT-based cell viability assay^2,55−59^ using ABCB1-expressing A2780/ADR cells compared to the concentration-effect curve of **33** against the sensitive A2780 cell line (open circles). Plotting of the resistance factors (GI_50_ values of A2780/ADR cells divided by the GI_50_ value of A2780 cells) derived from the GI_50_ values of graphs (A) and (B) against the used concentrations of compounds **24** (C) or **30** (D; 1.0, 2.0, 3.5, and 5.0 μM) allowed for the determination of the EC_50_ values (**24** = 0.793 ± 0.039 μM; **30** = 1.22 ± 0.01 μM).

The surprisingly high inhibitory power against ABCB1 prompted us to investigate whether the compounds (1.0, 2.0, 3.5, and 5.0 μM) were capable of sensitizing ABCB1-expressing A2780/ADR cells toward the antineoplastic agents **33**. Thus, we applied an MTT-based efficacy assay as reported earlier.^2,55−59^Figure 12 shows the concentration-effect curves of **33** without and with the supplementation of compounds **24** (A) or **30** (B). Both compounds (5 μM) completely sensitized ABCB1-expressing A2780/ADR cells against compound **33**. This sensitization is indicated by a shift of the concentration-effect curve of compound **33** from the right (=high concentrations of **33** needed to induce A2780/ADR cell death) to the left (=lower concentrations of **33** needed to induce A2780/ADR cell death). The concentration-effect curve of compound **33** in combination with 5 μM of either compound **24** or **30** resembles the concentration-effect curve of the sensitive cell line A2780. The half-maximal reversal concentration (EC_50_) of the compounds was 0.793 ± 0.039 μM (**24**; Figure 12C) and 1.22 ± 0.01 μM (**30**; Figure 12D), which matched the concentration range of their IC_50_ values. Thus, their potentiating effect with respect to the antiproliferative agent **33** against ABCB1-expressing A2780/ADR cells is due to the inhibition of ABCB1. Therefore, compounds **24** and **30** were dually effective against MCT1- and ABCB1-expressing cells (Table 5).

**Table 5:** Results of the MTT-Based Efficacy Assays^2,55−59^ Using the Antineoplastic Agent **33** Either without or in Combination with Either Compound **24** or **30** at Concentrations of 1.0, 2.0, 3.5, and 5.0 μM against ABCB1-Expressing A2780/ADR Cells Compared to the Effect of **33** Alone on Sensitive A2780 Cells^a^

no.	A2780/ADRcell toxicity **33** +0.0 μM cmpd.GI_50_ ± SEM [nM]	A2780/ADRcell toxicity **33** +1.0 μM cmpd.GI_50_ ± SEM [nM]	A2780/ADRcell toxicity **33** +2.0 μM cmpd.GI_50_ ± SEM [nM]	A2780/ADRcell toxicity **33** +3.5 μM cmpd.GI_50_ ± SEM [nM]	A2780/ADRcell toxicity **33** +5.0 μM cmpd.GI_50_ ± SEM [nM]	A2780 cell toxicity **33** +0.0 μM cmpd.GI_50_ ± SEM [nM]
**24**	802 ± 81	332 ± 50	214 ± 10	110 ± 1	32.2 ± 0.6	19.4 ± 1.7
**30**	802 ± 81	516 ± 45	165 ± 24	32.4 ± 3.1	12.9 ± 3.5	19.4 ± 1.7

a DMSO and culture medium were used to define 0% and 100%, respectively. GI_50_ values ± SEM of at least three independent experiments are shown.

### Computational Analyses

#### Molecular Docking of Lead MCT1 Inhibitors

**Figure 13:**
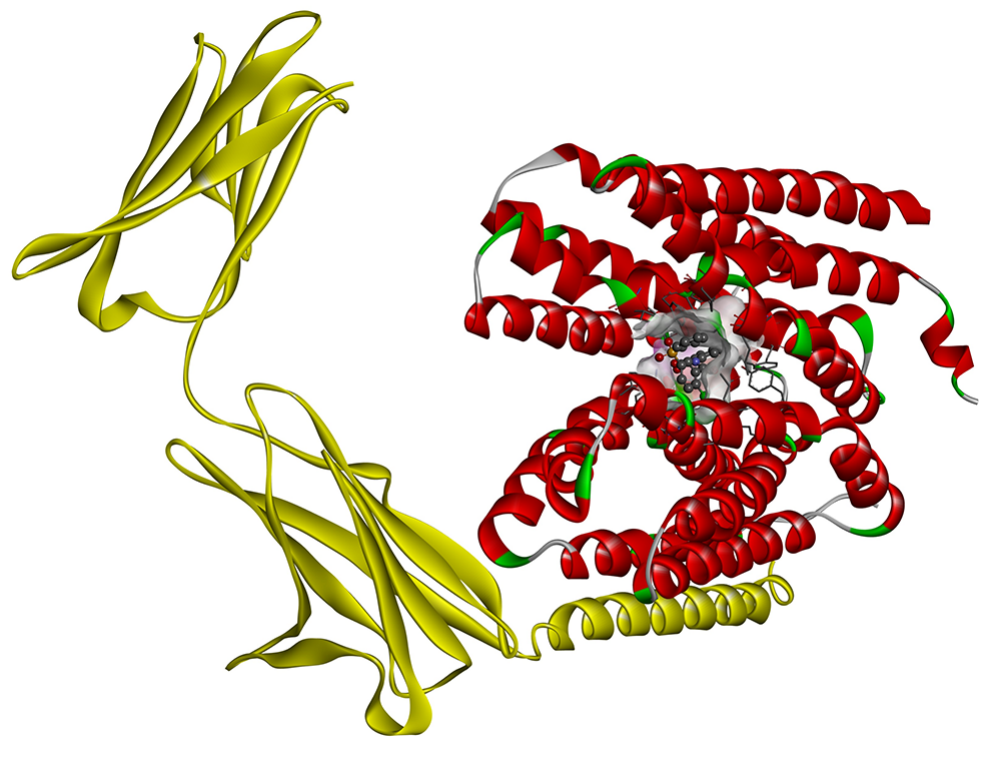
Ribbon structure of human MCT1 (red) in the outward-open conformation in complex with basagin-2 (yellow; PDB ID 7CKR)^19^ with the embedded MCT1 inhibitor **7**.

To get further insights into the ligand–transporter interactions and the structural background of MCT1 inhibition, molecular docking of the reference MCT1 inhibitors **7** and **8**, as well as the lead MCT1 inhibitors **24** and **30**, was applied using a recently released cryo-EM structure of human MCT1 (PDB ID: 7CKR).^19^Figure 13 shows the human MCT1 cryo-EM structure with compound **7** embedded within the transmembrane regions.

Molecular docking of reference inhibitor **7** resulted in the very same binding pose, as shown in the cryo-EM structure (PDB ID 7CKR;^19^Figure 13). It demonstrated a free binding energy of −10.7 kcal·mol^–1^ forming four conventional hydrogen bonds with Tyr34, Lys38, and Arg313. Moreover, **7** developed hydrophobic interactions (*i.e.*, π–π stacked, π–π T-shaped, π–alkyl, and alkyl) with Tyr34, Pro37, Lys38, Leu66, Phe278, Val282, Phe367, and Pro406. On the other hand, reference inhibitor **8** had an inferior free binding energy of −6.5 kcal·mol^–1^ and formed two conventional hydrogen bonds with Arg313. In addition, **8** demonstrated electrostatic π–cation as well as hydrophobic π–alkyl interaction with Lys38 in close proximity to Tyr34 and Leu66.

**Figure 14:**
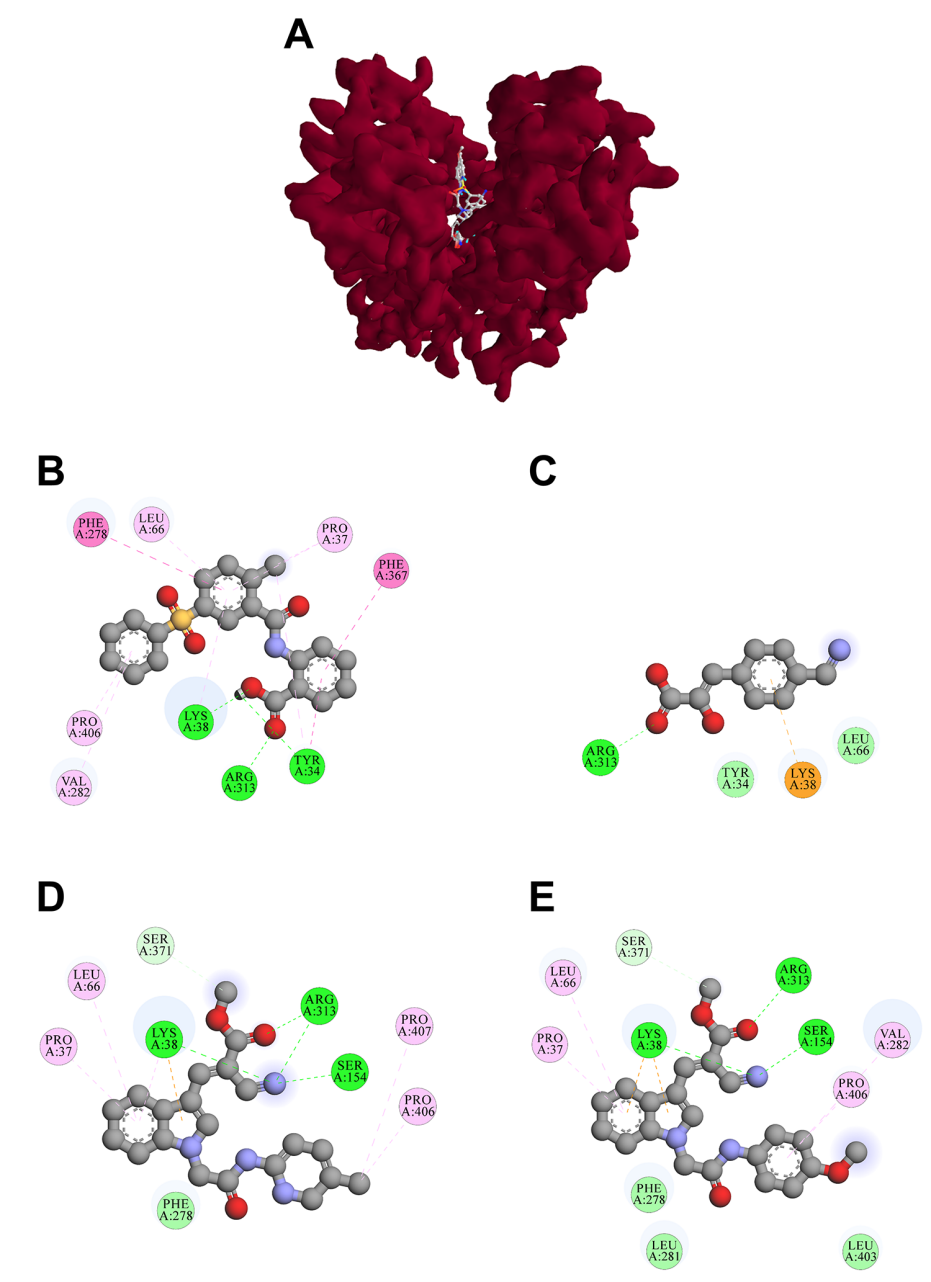
Top-ranking docking poses of compounds **7**, **8**, **24**, and **30** superimposed within MCT1 (PDB ID 7CKR;^19^ A). Two-dimensional (2D) representation of the binding poses of reference inhibitors **7** (B) and **8** (C) as well as the lead MCT1 inhibitors **24** (D) and **30** (E).

The most potent lead MCT1 inhibitor **24** had a very low free binding energy of −9.5 kcal·mol^–1^, which is comparable to the free binding energy of reference inhibitor **7**. Additionally, **24** formed three conventional hydrogen bonds with Lys38, Ser154, and Arg313 along with one carbon–hydrogen bond with Ser371. It additionally developed two electrostatic π–cation interactions with Lys38 and hydrophobic interactions (π–sigma, π–cation–alkyl) with Pro37, Leu66, Val282, and Pro406. The second most potent MCT1 inhibitor, **30**, had with −9.7 kcal·mol^–1^ a similar free binding energy compared to lead MCT1 inhibitor **24**, which is significantly lower compared to reference inhibitor **8** and comparable to reference inhibitor **7**. The interaction profile of compound **30** parallels the one of compound **24** due to its structural similarity. Compound **30** formed five conventional hydrogen bonds with Lys38, Ser154, and Arg313, along with one carbon–hydrogen bond with Ser371. Additionally, compound **30** displayed one electrostatic π–cation bond with Lys38 and numerous π–alkyl as well as alkyl interactions with Pro37, Lys38, Leu66, Pro406, and Pro407. Figure 14 outlines the interaction profiles of compounds **7** (B), **8** (C), **24** (D), and **30** (E).

The found results revealed that the lead MCT1 inhibitors **24** and **30** had binding affinities similar to the golden reference compound **7**. Compounds **7**, **8**, **24**, and **30** targeted the same binding pocket that was already earlier identified by a structure-focused study.^19^ Furthermore, the identified interactions of compounds **7**, **8**, **24**, and **30** resembled in the same binding pocket embedded by not only Lys38 and Arg313 (shared amongst all four compounds) but also Pro37, Leu66, and Pro406 (shared amongst compounds **7**, **24**, and **30**) despite the structural variation between reference inhibitors and indole derivatives. Thus, these interactions found the very basis of MCT1 inhibition.

#### Determination of Physicochemical Properties

Molecular–structural characteristics, such as hydrogen-(H)-bond donors, H-bond acceptors, or rotatable bonds are important for the pharmacokinetics and pharmacodynamics of drugs.^60,72^ Also, certain physicochemical properties, such as the calculated octanol/water partition coefficient (CLog *P*), molecular weight (MW), molar refractivity (MR), or the topological polar surface area (TPSA), shape cellular penetration and drug effectiveness.^60,72^ Thus, these and other important parameters of the investigated compounds were calculated by applying the online web service SwissADME (http://www.swissadme.ch).^75^Table 6 provides these data including the overall drug likeliness of the compounds according to the Lipinski rule-of-five and their interaction profile with the pharmacokinetic-influencing ABC transporters ABCB1, ABCC1, and ABCG2.

**Table 6:** Assessment of Indole Derivatives **17**–**32** with Respect to Important Molecular–Structural and Physicochemical Properties as Determined Using the Web Service SwissADME (http://www.swissadme.ch)^75^ Including Other Pharmacokinetic-Influencing Factors

no.	chemical formula	heavy atoms	rotatable bonds	H-bond donor	H-bond acceptor	CLog *P*^a^	MW	MR	TPSA^b^	rule-of-five	ABCB1 inhibition^c^	ABCC1 inhibition^c^	ABCG2 inhibition^c^
**17**	C_21_H_17_N_3_O_3_	27	7	1	4	3.06	359.38	102.58	84.12	X	X		
**18**	C_25_H_19_N_3_O_3_	31	7	1	4	4.21	409.44	120.09	84.12	X	X		
**19**	C_25_H_19_N_3_O_3_	31	7	1	4	4.21	409.44	120.09	84.12	X	X		
**20**	C_22_H_19_N_3_O_3_	28	7	1	4	3.37	373.40	107.55	84.12	X	X	X	
**21**	C_22_H_19_N_3_O_3_	28	7	1	4	3.37	373.40	107.55	84.12	X	X	X	
**22**	C_22_H_19_N_3_O_4_	29	8	1	5	3.07	389.40	109.07	93.35	X	X	X	X
**23**	C_22_H_19_N_3_O_4_	29	8	1	5	3.07	389.40	109.07	93.35	X	X	X	X
**24**	C_22_H_19_N_3_O_4_	29	8	1	5	3.07	389.40	109.07	93.35	X	X	X	
**25**	C_21_H_16_FN_3_O_3_	28	7	1	5	3.62	377.37	102.54	84.12	X	X	X	
**26**	C_21_H_16_ClN_3_O_3_	28	7	1	4	3.71	393.82	107.59	84.12	X	X	X	
**27**	C_21_H_15_Cl_2_N_3_O_3_	29	7	1	4	4.37	428.27	112.60	84.12	X	X	X	
**28**	C_21_H_16_BrN_3_O_3_	28	7	1	4	3.82	438.27	110.28	84.12	X	X		
**29**	C_20_H_16_N_4_O_3_	27	7	1	5	2.46	360.37	100.37	97.01	X			
**30**	C_21_H_18_N_4_O_3_	28	7	1	5	2.76	374.39	105.34	97.01	X	X	X	
**31**	C_20_H_15_BrN_4_O_3_	28	7	1	5	3.22	439.26	108.07	97.01	X	X	X	
**32**	C_20_H_16_N_4_O_3_	27	7	1	5	2.46	360.37	100.37	97.01	X	X	X	

a CLog *P* was determined applying the atomistic method.^76^

b TPSA was determined applying the fragment-based method.^77^

c Compounds were considered as inhibitors if they reached 20% (+SEM) inhibition compared to the reference inhibitors **33**–**35**; X = fulfilled.

## Discussion and Conclusions

### Standing of Indole Derivatives

The discovery of novel drug targets opening up new therapeutic options is the challenge of medicinal chemistry today. However, the discovery is only the first of multiple steps to access these discovered targets for pharmacotherapy. MCT1 has been described as a critical factor of cancer survival for several years now,^10,13−16^ and yet, the fund of compounds addressing this SLC transporter with sufficient efficacy and preferable pharmacological profile is highly limited.^18−31^ Only roughly 100 compounds have been described to address this underexplored drug target,^18−31^ including the drugs and druglike compounds that were the first ligands with poor inhibitory activity, such as **1** and **3**.^31^ Potent MCT1 inhibitors with affinities in the single-digit nanomolar concentration range or even at subnanomolar concentrations indeed exist;^18,28,29,32,34^ however, these compounds exclusively belong to the few known molecular–structural classes, as outlined in the Introduction section, and do not contribute to structural diversity. Up to date, only compound **4** reached the clinical drug development stage (NCT01791595, completed 2022);^18^ the screening of chemical space as well as the exploration and exploitation of novel molecular–structural compound classes necessitates. Indole represented a promising scaffold considering the initial indole- and indole-like scaffold-containing hit molecules addressing MCT1.^13,35−37^

In our study, we provided important SARs of indole derivatives and, thus, explored these as a novel molecular–structural class of MCT1 inhibitors. We obtained compounds with higher inhibitory potency compared to a significant fraction of the ∼100 reported MCT1 inhibitors in the literature,^18−31^ including pteridine derivatives,^33^ most coumarin derivatives,^25^ and many cinnamic acid derivatives including reference compound **8**.^21,24,31^ Nevertheless, many earlier reported MCT1 inhibitors have demonstrated inhibition concentrations significantly below the determined IC_50_ values as found within our work,^18^ including thieno[2,3-*d*]pyrimidin-2,4-diones,^28,32^ pyrrolo[3,4-*d*]pyridazinones,^29^ or **7**(34) as well as many cinnamic acid derivatives.^22,24^ However, three aspects have to be considered to put the bioactivity profile of indole derivatives into the right perspective:

(i) As MCT1 inhibitors are barely known with only roughly 100 representatives in the literature,^18−31^ no standardized assay procedures have been developed compared to methodologically better-explored protein (super)families, as, for example, ABC transporters.^2,7,52−61,63,66,72^ This led to rather diverse assay setups with a minor number of independent confirmatory studies to determine functional compound bioactivities, limiting the overall comparability of data. These diverse assays included nonfunctional MTT-based cell viability assays,^21,25,33^ pH-dependent functional fluorescence assays,^34^ functional radiotracer assays [*e.g.*, ^3^H-labeling of test candidates,^34^^14^C-labeling of MCT1 substrates (^14^C-lactate^21,22,24,25,31,33,34^), or photoaffinity labeling (*e.g.*, by ^125^I-labeling^29^)]—often focusing on binding affinity^29,34^ rather than inhibitory power (IC_50_).^21,22,24,25,31,33,34^ Furthermore, the applied cell lines were diverse, including either genuine MCT1-(over)expressing (cancer) cells (*e.g.*, A-549,^14,15^ DLD-1,^34^ MCF-7,^16,21,33^ RBE4,^21,22,24^ or SiHa^25^) but also transfected artificial expression systems (*e.g.*, *Xenopus laevis* oocytes^31,34^)—both of which showed differences in MCT1 expression, resulting in variations in assay response and bioactivity data. Thus, the data presented in our work can only to a very limited extent be compared to literature data, as literature data itself can only be compared with each other to a limited extent in the current data situation.
(ii) Due to the mentioned variations in terms of the (non)functional assays, detection methodologies, and expression systems, even reference compounds of MCT1 inhibition have been reported with huge variations in inhibitory power. Compound **7**, for example, has been assessed in two different assay systems (fluorescence-based *vs* radioactivity-based) revealing IC_50_ values between 1 and 85 nM—with almost 2 orders of magnitude difference.^34^ The within this work used reference inhibitor **8** presented a similar picture in the literature from submillimolar to low micromolar concentration ranges.^21,22,25^ Thus again, the overall comparability of the literature data is limited.
(iii) To oppose the limited data situation and reduce data variation, the development of novel, easier-to-apply, reliable, and robust assays is an important factor, also in terms of the exploration of novel pharmacological drug targets. Particularly, the functional 3-BP cell viability assay that was used within our work is such a new assay that has not found its way into broad experimental use yet.^20,39^ This lack of application is of course accompanied by uncertainty with respect to the outcome data. However, our assay has several very pronounced advantages compared to assay setups as reported in point (i): (a) most assays conducted with respect to MCT1 are based on radioactivity measurement^21,22,24,25,29,31,33,34^ and thus are constrained to regulatory requirements, which limit general application. 3-BP, on the other hand, is a regular chemical without regulatory constraints, promoting general use; (b) radiotracer assays are accompanied—apart from regulatory constraints—by complex protocols, which makes the determination of full-blown concentration-effect curves or even high-throughput screenings a challenge. In contrast, the 3-BP assay is an easy-to-perform assay with a protocol comparable to other functional and/or MTT-based assays as described for other protein (super)families, as, for example, ABC transporters;^2,7,52−61,63,66,72^ and (c) furthermore, the 3-BP assay demonstrated extreme reliability and robustness, as can be seen from the low deviations depicted either in Table 1 as well as in Figure 3.

### Targeting Monocarboxylate Transport as Potential Anticancer Strategy

Lead compound **24** decreased the cell viability of MCT1-expressing cancer cells due to pyruvate and subsequent glucose deprivation and increased the susceptibility of MCT1-expressing cells to the antineoplastic agent **33**. Further biological investigations showed that compound **24** induced cell cycle arrest and cancer cell apoptosis. These findings present compound **24** as one of the most effective MCT1 inhibitors with an optimized additional pharmacological profile, demonstrating its aptitude as an adjuvant therapeutic together with first-line antineoplastic agents in use to treat several types of cancers.

None of the 16 evaluated compounds demonstrated an inhibitory feature against MCT4, the other promising anticancer target. However, the indole-like compound **10** was shown previously to inhibit the mitochondrial pyruvate carrier (MCP1).^37^ Thus, an interaction of the herein presented indole derivatives is thinkable, which would be of advantage as MCP1 has been identified as another potential anticancer drug target.^78,79^ Further investigations are warranted to explore these novel potential therapeutic options.

### Polypharmacology of Indole Derivatives

Interestingly, indoles turned out to have a rich polypharmacological profile as we identified many inhibitory interactions with ABC transporters. MDR caused by the upregulation of ABC transporters remains until today another big obstacle in anticancer therapy,^2,4,5^*e.g.*, by conferring resistance of cancer cells against antineoplastic agents, such as compound **33**. Compound **24** inhibited the multidrug transporter ABCB1 with an IC_50_ of roughly 1 μM, and it completely sensitized ABCB1-expressing cancer cells at 5 μM, which is a moderate potency in terms of the efficacy against ABCB1. In essence, in addition to its already optimized pharmacological profile, compound **24**’s polypharmacological nature addressing two phylogenetically unrelated but functionally similar membrane-bound transporters not only makes it the first synthesis-derived member of its kind but also represents a very promising starting structure for ongoing biological investigations.

In line with the polypharmacological nature of compound **24** is the discovery of the focused^71^ pan-ABC transporter inhibitors **22** and **23**, which additionally addressed MCT1, presenting themselves as the first synthesis-derived pan-ABC/SLC transporter modulators. The usefulness of pan-agents has been broadly discussed in the literature,^52−54,60,72−74^ and particularly intersuperfamily addressing molecules may be of great use to further explore target space and identify as well as validate potential pharmacological drug targets of the future.

## Experimental Section

### Chemistry

#### Materials

Chemicals and solvents were purchased from Omkar Traders (Mumbai, India), Sigma-Aldrich (Mumbai, India), and Sisco Research Laboratories (Mumbai, India) and were used without further purification. All reactions were carried out under an inert atmosphere, and thin-layer chromatography (TLC) was applied to monitor the reaction progress using an aluminum plate coated with silica gel 60 F_254_ (Merck Millipore, Billerica, MA). Chloroform/methane (95%/5%) was used as an eluent, and the results were viewed under a UV cabinet (Desaga, Biostep, Burkhardtsdorf, Germany) at a wavelength of 254 nm. Both column chromatography on silica gel (60–120 μm; Merck, Mumbai, India) and flash chromatography (Combiflash RF, Teledyne ISCO, NE, Lincoln) were used to accomplish the chromatographic purification using dichloromethane/methane 98%/2%.

The identity of compounds **17**–**32** was determined by Fourier transform infrared (FTIR, Spectrum RXI, Perkin Elmer Spectra, Waltham, MA) and ^1^H NMR spectroscopy (Bruker Advance DX 400 MHz, Billerica, MA). The chemical shifts (δ) were expressed in ppm in relation to the internal standard tetramethylsilane, and multiplicity of signals was indicated as singlet (s), doublet (d), doublet of doublets (dd), triplet (t), triplet of doublets (td), pentet (p), and multiplet (m). The molecular mass of compounds was determined by liquid chromatography-mass spectrometry (LCMS) analysis (LCMS-8040, Shimadzu, Kyoto, Japan), and all compounds are >95% pure by high-performance liquid chromatography (HPLC, Shimadzu, Kyoto, Japan). The melting point of the compounds was determined using the melting point apparatus (Veego, Mumbai, India).

#### 1*H*-Indole 3-carbaldehyde (**13**)

Intermediate **13** was prepared as described in the literature with minor modifications.^80^ After being cooled in an ice–salt bath, *N,N*-dimethylformamide was subjected to phosphoryl chloride (POCl_3_; 2 equiv). The resulting reaction mixture was stirred for 20 min before a solution of indole (**12**, 1 equiv) in *N,N*-dimethylformamide (25 mL) was added. The mixture was stirred for 4 h at room temperature. After adding a trace quantity of crushed ice, the mixture was basified with 5 M sodium hydroxide (pH = 14). Intermediate **13** precipitated after stirring for 1 h at room temperature, which was filtered, dried, and used without further purification and characterization.

#### Ethyl 2-(3-Formyl-1*H*-indol-1-yl) (**14**)

Cesium carbonate (Cs_2_CO_3_; 1.5 equiv) was added to a solution of intermediate **13** (1 equiv) in DMF (5 mL), and the reaction mixture was stirred for 10 min at room temperature. Ethyl bromoacetate (1.1 equiv) was added, and the reaction mixture was stirred for 6 h at room temperature. Ice-cold water was added to the mixture, forming intermediate **14**. The precipitate was filtered, dried, recrystallized from ethanol, and used without further characterization.

#### 2-(3-Formyl-1*H*-indol-1-yl) Acetic Acid (**15**)

NaOH (4 equiv) was added to a stirred solution of intermediate **14** in ethanol at 0 °C, followed by further stirring for 1 h. The solvent was concentrated, and eventually, the reaction mixture was acidified with 1 M hydrochloric acid. The precipitated intermediate **15** was filtered off, dried, recrystallized from ethanol, and used without further characterization.

#### 2-(3-(2-Cyano-3-methoxy-3-oxoprop-1-en-1-yl)-1*H*-indol-1-yl) Acetic Acid (**16**)

Intermediate **15** (1 equiv) and methyl cyanoacetate (1 equiv) in methanol (10 mL) were mixed with catalytic amounts of piperidine, and the mixture was stirred for 7 h. The solvent was evaporated under reduced pressure, and the mixture was acidified with 2 M HCl forming intermediate **16**. The formed precipitate was filtered off, dried, recrystallized from ethanol, and used without further characterization.

#### General Procedure for the Preparation of Compounds (**17**–**16**)

POCl_3_ (1.2 equiv) was added to a stirred solution of intermediate **16** (1 equiv) in dichloromethane and catalytic amounts of pyridine, which was stirred for 20 min at 0 °C. The respective substituted aromatic amine (1.1 equiv) was added at 0 °C, and the mixture was stirred for 6 h, followed by stirring for 30 min at room temperature. The reaction mixture was poured into ice-cold water (100 mL), and the respective target compound was extracted with EtOAc (2 × 100 mL). The combined organic phases were dried over Na_2_SO_4_, filtered, and concentrated under reduced pressure yielding the respective target compound.

#### Methyl-2-cyano-3-(1-(2-oxo-2-(phenylamino)ethyl)-1*H*-indol-3-yl) Acrylate (**17**)

Yellow crystals; yield: 65%; melting point: 192–194 °C; FTIR (cm^–1^): 3292.94 (NH), 2214.52 (CN), 1721.24 (C=O), 1673.19 (C=O), 1593.89 (C=C); ^1^H NMR (400 MHz, DMSO-*d*_6_) δ 10.56 (s, 1H), 8.67 (s, 1H), 8.58 (s, 1H), 8.08–7.84 (m, 1H), 7.61 (dd, *J* = 8.1, 5.3 Hz, 3H), 7.33 (m, 4H), 7.19–6.87 (m, 1H), 5.35 (s, 2H), 3.84 (s, 3H); MS (*m*/*z*) calculated for C_21_H_17_N_3_O_3_: 359.127, found: 377.25 [M + H_2_O] and 382.150 [M + Na]^+^; purity (HPLC): 98%.

#### Methyl-2-cyano-3-(1-(2-(naphthalen-1-ylamino)-2-oxoethyl)-1*H*-indol-3-yl) Acrylate (**18**)

Yellow solid; yield: 66%; melting point: 201–203 °C; FTIR (cm^–1^): 3118.97 (NH), 2215.49 (CN), 1659.00 (C=O), 1590.59 (C=O), 1515.83 (C=C); ^1^H NMR (400 MHz, DMSO-*d*_6_) δ 10.44 (s, 1H), 8.70 (s, 1H), 8.56 (s, 1H), 8.16 (d, *J* = 8.1 Hz, 1H), 7.99 (d, *J* = 7.9 Hz, 1H), 7.92 (d, *J* = 7.8 Hz, 1H), 7.75 (d, *J* = 8.2 Hz, 1H), 7.68 (d, *J* = 7.6 Hz, 2H), 7.55 (p, *J* = 6.8 Hz, 2H), 7.45 (t, *J* = 7.9 Hz, 1H), 7.37 (t, *J* = 7.6 Hz, 1H), 7.30 (t, *J* = 7.4 Hz, 1H), 5.51 (s, 2H), 3.81 (s, 3H); MS (*m*/*z*) calculated for C_25_H_19_N_3_O_3_: 409.143, found: 427.400 [M + H_2_O]; purity (HPLC) 100%.

#### Methyl-2-cyano-3-(1-(2-(naphthalen-2-ylamino)-2-oxoethyl)-1*H*-indol-3-yl) Acrylate (**19**)

Yellow solid; yield: 82%, melting point: 193–195 °C: FTIR (cm^–1^): 3255.87 (NH), 2219.58 (CN), 1670.16 (C=O), 1594.87 (C=O), 1517.83 (C=C); ^1^H NMR (400 MHz, DMSO-*d*_6_) δ 10.36 (s, 1H), 8.62 (s, 1H), 8.54 (s, 1H), 8.00–7.94 (m, 1H), 7.59–7.52 (m, 1H), 7.50–7.43 (m, 2H), 7.36–7.21 (m, 2H), 6.91–6.78 (m, 2H), 5.27 (s, 2H), 3.80 (s, 3H), 3.68 (s, 3H); MS calculated. for C_25_H_19_N_3_O_3_: 409.143, found: 427.400 [M + H_2_O]; purity (HPLC): 100%.

#### Methyl-2-cyano-3-(1-(2-oxo-2-(*o*-tolylamino)ethyl)-1*H*-indol-3-yl) Acrylate (**20**)

Yellow solid; yield: 73%; melting point: 161–163 °C: FTIR (cm^–1^): 3279.55 (NH), 2213.31 (CN), 1661.61 (C=O), 1573.95 (C=O), 1516.34 (C=C); ^1^H NMR (400 MHz, DMSO-*d*_6_) δ 9.89 (s, 1H), 8.67 (s, 1H), 8.58 (s, 1H), 8.01 (d, *J* = 7.8 Hz, 1H), 7.64 (d, *J* = 8.1 Hz, 1H), 7.45–7.28 (m, 3H), 7.26–7.19 (m, 1H), 7.20–7.05 (m, 2H), 5.39 (s, 2H), 3.84 (s, 3H), 2.24 (s, 3H); MS (*m*/*z*) calculated for C_22_H_19_N_3_O_3_: 373.143, found: 374.400 [M + H]^+^ and 391.400 [M + H_2_O]; purity (HPLC): 98%.

#### Methyl-2-cyano-3-(1-(2-oxo-2-(*p*-tolylamino)ethyl)-1*H*-indol-3-yl) Acrylate (**21**)

White solid; yield: 67%, melting point: 195–197 °C: FTIR (cm^–1^): 3314.69 (NH), 2210.98 (CN), 1678.03 (C=O), 1587.18 (C=O), 1514.71 (C=C); ^1^H NMR (400 MHz, DMSO-*d*_6_) δ 10.42 (s, 1H), 8.63 (s, 1H), 8.54 (s, 1H), 8.02–7.93 (m, 1H), 7.61–7.53 (m, 1H), 7.47–7.41 (m, 2H), 7.35–7.23 (m, 2H), 7.09 (d, *J* = 8.1 Hz, 2H), 5.29 (s, 2H), 3.80 (s, 3H), 2.21 (s, 3H); MS (*m*/*z*) calculated for C_22_H_19_N_3_O_3_; 373.143, found: 374.200 [M + H]^+^ and 396.200 [M + Na]^+^; purity (HPLC): 99%.

#### Methyl-2-cyano-3-(1-(2-((2-methoxyphenyl)amino)-2-oxoethyl)-1*H*-indol-3-yl) Acrylate (**22**)

Gray solid; yield: 68%; melting point: 182–184 °C: FTIR (cm^–1^): 3281.03 (NH), 2216.41 (CN), 1667.56 (C=O), 1587.62 (C=O), 1518.59 (C=C); ^1^H NMR (400 MHz, DMSO-*d*_6_) δ 9.74 (s, 1H), 8.63 (s, 1H), 8.54 (s, 1H), 8.00–7.94 (m, 1H), 7.88 (d, *J* = 8.0 Hz, 1H), 7.59 (d, *J* = 8.0 Hz, 1H), 7.37–7.24 (m, 2H), 7.06 (m 2H), 6.85 (m, 1H), 5.41 (s, 2H), 3.85 (s, 3H), 3.80 (s, 3H); MS (*m*/*z*) calculated for C_22_H_19_N_3_O_4_: 389.138, found: 390.400 [M + H]^+^ and 407.400 [M + H_2_O]; purity (HPLC): 97%.

#### Methyl-2-cyano-3-(1-(2-((3-methoxyphenyl)amino)-2-oxoethyl)-1*H*-indol-3-yl) Acrylate (**23**)

Yellow solid; yield: 73%, melting point: 184–186 °C: FTIR (cm^–1^): 3330.26 (NH), 2212.83 (CN), 1685.80 (C=O), 1587.31 (C=O), 1515.92 (C=C); ^1^H NMR (400 MHz, DMSO-*d*_6_) δ 10.51 (s, 1H), 8.63 (s, 1H), 8.55 (s, 1H), 8.01–7.94 (m, 1H), 7.57 (d, *J* = 7.9 Hz, 1H), 7.34–7.14 (m, 4H), 7.08 (dd, *J* = 7.9, 1.9 Hz, 1H), 6.62 (dd, *J* = 8.2, 2.5 Hz, 1H), 5.31 (s, 2H), 3.81 (s, 3H), 3.67 (s, 3H); MS (*m*/*z*) calculated for C_25_H_19_N_3_O_4_: 389.138, found: 390.400 [M + H]^+^ and 407.400 [M + H_2_O]; purity (HPLC): 100%.

#### Methyl-2-cyano-3-(1-(2-((4-methoxyphenyl)amino)-2-oxoethyl)-1*H*-indol-3-yl) Acrylate (**24**)

Yellow solid; yield: 80%; melting point: 187–189 °C: FTIR (cm^–1^): 3288.77 (NH), 2215.56 (CN), 1671.76 (C=O), 1587.92 (C=O), 1514.10 (C=C); ^1^H NMR (400 MHz, DMSO-*d*_6_) δ 10.36 (s, 1H), 8.62 (s, 1H), 8.54 (s, 1H), 8.00–7.94 (m, 1H), 7.59–7.52 (m, 1H), 7.50–7.43 (m, 2H), 7.36–7.21 (m, 2H), 6.91–6.78 (m, 2H), 5.27 (s, 2H), 3.80 (s, 3H), 3.68 (s, 3H); MS (*m*/*z*) calculated for C_22_H_19_N_3_O_4_: 389.138, found: 390.400 [M + H]^+^ and 407.400 [M + H_2_O]; purity (HPLC): 100%.

#### Methyl-2-cyano-3-(1-(2-((4-fluorophenyl)amino)-2-oxoethyl)-1*H*-indol-3-yl) Acrylate (**25**)

Pale yellow solid; yield: 71%; melting point: 190–192 °C: FTIR (cm^–1^): 3259.35 (NH), 2205.56 (CN), 1673.09 (C=O), 1572.50 (C=O), 1512.60 (C=C); ^1^H NMR (400 MHz, DMSO-*d*_6_) δ 10.56 (s, 1H), 8.63 (s, 1H), 8.54 (s, 1H), 7.99–7.95 (m, 1H), 7.57 (m, 3H), 7.30 (m, 2H), 7.16–7.10 (m, 2H), 5.30 (s, 2H), 3.81 (s, 3H); MS (*m*/*z*) calculated for C_21_H_16_FN_3_O_3_: 377.118, found: 395.300 [M + H_2_O]; purity (HPLC): 100%.

#### Methyl-3-(1-(2-((4-chlorophenyl)amino)-2-oxoethyl)-1*H*-indol-3-yl)-2-cyanoacrylate (**26**)

Yellow solid; yield: 79%, melting point: 165–167 °C: FTIR (cm^–1^): 3259.35 (NH), 2205.56 (CN), 1673.09 (C=O), 1572.50 (C=O), 1512.60 (C=C); ^1^H NMR (400 MHz, DMSO-*d*_6_) δ 10.66 (s, 1H), 8.63 (s, 1H), 8.54 (s, 1H), 7.99–7.94 (m, 1H), 7.58 (td, *J* = 5.9, 5.3, 3.4 Hz, 3H), 7.38–7.22 (m, 4H), 5.32 (s, 2H), 3.81 (s, 3H); MS (*m*/*z*) calculated for C_21_H_16_ClN_3_O_3_: 393.088, found: 411.400 [M + H_2_O]; purity (HPLC) 100%.

#### Methyl-2-cyano-3-(1-(2-((2, 6-dichlorophenyl)amino)-2-oxoethyl)-1*H*-indol-3-yl) Acrylate (**27**)

Yellow solid; yield: 76%; melting point: 186–188 °C: FTIR (cm^–1^): 3121.03 (NH), 2215.61 (CN), 1659.20 (C=O), 1591.52 (C=O), 1515.21 (C=C); ^1^H NMR (400 MHz, DMSO-*d*_6_) δ 11.20 (s, 1H), 8.64 (s, 1H), 8.54 (s, 1H), 8.46 (d, *J* = 2.4 Hz, 1H), 8.00–7.90 (m, 3H), 7.59–7.53 (m, 1H), 7.30 (m, 2H), 5.38 (s, 2H), 3.80 (s, 3H); MS (*m*/*z*) calculated for C_21_H_15_Cl_2_N_3_O_3_: 427.049, found 425.950 [M – H]^−^ and 463.950 [M + K]^−^; purity (HPLC): 96%.

#### Methyl-3-(1-(2-((4-bromophenyl)amino)-2-oxoethyl)-1*H*-indol-3-yl)-2-cyanoacrylate (**28**)

Beige solid; yield: 69%; melting point: 187–189 °C: FTIR (cm^–1^): 3263.44 (NH), 2215.99 (CN), 1679.00 (C=O), 1587.14 (C=O), 1517.21 (C=C); ^1^H NMR (400 MHz, DMSO-*d*_6_) δ 10.62 (s, 1H), 8.63 (s, 1H), 8.53 (d, *J* = 7.2 Hz, 2H), 8.00–7.91 (m, 1H), 7.59–7.50 (m, 3H), 7.29 (m, 3H), 5.32 (s, 2H), 3.80 (s, 3H); MS (*m*/*z*) calculated for C_21_H_16_BrN_3_O_3_: 437.038, found 476.150 [M + K]^+^; purity (HPLC): 99%.

#### Methyl-2-cyano-3-(1-(2-oxo-2-(pyridin-2-ylamino)ethyl)-1*H*-indol-3-yl) Acrylate (**29**)

Colorless solid; yield: 71%; melting point: 167–169 °C; FTIR (cm^–1^): 2952.50 (NH), 2210.55 (CN), 1699.57 (C=O), 1566.64 (C=O), 1515.37 (C=C); ^1^H NMR (400 MHz, DMSO-*d*_6_) δ 11.11–11.03 (m, 2H), 8.68 (s, 1H), 8.56 (d, *J* = 7.3 Hz, 1H), 8.36 (dd, *J* = 4.9, 1.9 Hz, 1H), 8.04–7.93 (m, 2H), 7.77 (td, *J* = 7.9, 2.0 Hz, 1H), 7.61 (d, *J* = 8.0 Hz, 1H), 7.40–7.28 (m, 3H), 7.13 (dd, *J* = 7.3, 5.0 Hz, 1H), 5.42 (s, 2H), 3.84 (s, 3H); MS (*m*/*z*) calculated for C_20_H_16_N_4_O_3_: 360.122, found: 361.40 [M + H]^+^ and 383.300 [M + Na]^+^; purity (HPLC): 98%.

#### Methyl-2-cyano-3-(1-(2-((5-methylpyridin-2-yl)amino)-2-oxoethyl)-1*H*-indol-3-yl) Acrylate (**30**)

Yellow solid; yield: 69%; melting point: 178–180 °C: FTIR (cm^–1^): 3281.03 (NH), 2216.41 (CN), 1667.58 (C=O), 1587.62 (C=O), 1518.59 (C=C); ^1^H NMR (400 MHz, DMSO-*d*_6_) δ 10.93 (s, 1H), 8.63 (s, 1H), 8.57–8.49 (m, 1H), 8.16 (d, *J* = 2.3 Hz, 1H), 7.90 (dd, *J* = 51.7, 8.2 Hz, 2H), 7.56 (dd, *J* = 8.0, 4.5 Hz, 2H), 7.30 (p, *J* = 7.1 Hz, 2H), 5.35 (s, 2H), 3.80 (s, 3H), 2.21 (s, 3H); MS (*m*/*z*) calculated for C_21_H_18_N_4_O_3_: 374.138, found: 375.300 [M + H]^+^; purity (HPLC): 100%.

#### Methyl-3-(1-(2-((5-bromopyridin-2-yl)amino)-2-oxoethyl)-1*H*-indol-3-yl)-2-cyanoacrylate (**31**)

Brown solid; yield 74%; melting point: 177–179 °C; FTIR (cm^–1^): 3288.77 (NH), 2215.66 (CN), 1671.76 (C=O), 1587.92 (C=O), 1514.10 (C=C); ^1^H NMR (400 MHz, DMSO-*d*_6_) δ 10.93 (s, 1H), 8.63 (s, 1H), 8.57–8.49 (m, 1H), 8.16 (d, *J* = 2.3 Hz, 1H), 7.90 (dd, *J* = 51.7, 8.2 Hz, 2H), 7.56 (dd, *J* = 8.0, 4.5 Hz, 2H), 7.30 (m, 2H), 5.35 (s, 2H), 3.80 (s, 3H), 2.21 (s, 3H); MS (*m*/*z*) calculated for C_20_H_15_BrN_4_O_3_: 438.033, found: 439.200 [M + H]^+^ and 441.200 [M + 3H]^3+^; purity (HPLC): 100%.

#### Methyl-2-cyano-3-(1-(2-oxo-2-(pyridin-3-ylamino)ethyl)-1*H*-indol-3-yl) Acrylate (**32**)

Yellow solid; yield 73%; melting point: 179–181 °C; IR (cm^–1^): 2952.50 (NH), 2210.55 (CN), 1698.57 (C=O), 1573.93 (C=O), 1515.37 (C=C); ^1^H NMR (400 MHz, DMSO-*d*_6_) δ 10.85 (s, 1H), 8.76 (d, *J* = 2.6 Hz, 1H), 8.67 (s, 1H), 8.58 (s, 1H), 8.29 (dd, *J* = 4.8, 1.6 Hz, 1H), 8.02 (m, 2H), 7.65–7.54 (m, 1H), 7.37–7.24 (m, 2H), 5.40 (s, 2H), 3.84 (s, 3H). MS (*m*/*z*) calculated for C_20_H_16_N_4_O_3_: 360.122, found: 361.350 [M + H]^+^; purity (HPLC) 98%.

### Biological Evaluation

#### Materials

The reference MCT1 inhibitor **8** has been synthesized in-house.^81^ The reference ABCB1 inhibitor **34** and the reference ABCG2 inhibitor **36** were procured from Sigma-Aldrich (St. Louis, MO). The standard ABCC1 inhibitor **35** was synthesized as reported previously.^58,61^ The cytotoxic functional MCT1 tracer 3-BP and MTT were purchased from Sigma-Aldrich (St. Louis, MO). The fluorescence dyes calcein AM, daunorubicin, and pheophorbide A were received from Calbiochem (EMD Chemicals, San Diego), EMD Millipore (Billerica, MA), and Cayman Chemicals (Ann Arbor, MI). All other chemicals were obtained from Sigma-Aldrich (St. Louis, MO) and VWR (Radnor, PA). Compounds **17**–**32** were stored at −20 °C as 10 mM stock solutions. Dilution series and the experimental cell culture were performed in either phenol red-free Dulbecco’s modified Eagle’s media (DMEM; GE Healthcare, Chicago, IL) or phenol red-free RPMI-1640 (GE Healthcare, Chicago, IL) without additional supplements.

#### Cell Culture

The MCT1-expressing lung and breast cancer cell lines A-549^14,15^ and MCF-7,^16^ the MCT4-expressing cancer cell line MDA-MB-231,^22,41,42^ as well as the non-MCT1-expressing, noncancerous murine embryonic cell line NIH/3T3 were obtained from the National Centre for Cell Science (NCCS; Pune, India). The ABCB1-, ABCC1-, and ABCG2-expressing cell lines A2780/ADR, H69AR, and MDCK II BCRP were a generous gift of Prof. Dr. Finn K. Hansen and Prof. Dr. Gerd Bendas (Pharmaceutical and Cellbiological Chemistry, University of Bonn, Germany).

A-549, MCF-7, MDA-MB-231, NIH/3T3, and MDCK II BCRP cells were cultured in DMEM cell culture media (Genetix Biotech Asia, New Delhi, India, and Biowest, Nuaillé, France) supplemented with 10% fetal bovine serum (FBS; Genetix Biotech Asia, New Delhi, India, and Biowest, Nuaillé, France). A2780/ADR and H69AR cells were cultivated using RPMI-1640 cell culture media (Biowest, Nuaillé, France) supplemented with 10 and 20% FBS (Biowest, Nuaillé, France), respectively. All cell lines were additionally supplemented with streptomycin (50 μg/μL), penicillin G (50 U/mL), and L-glutamine (2 mM; Genetix Biotech Asia, New Delhi, India, and Biowest, Nuaillé, France). The cells were stored under liquid nitrogen (media: 90%; DMSO: 10%) and cultivated at 37 °C under a 5% CO_2_-humidified atmosphere. A trypsin-ethylenediamine tetraacetic acid (EDTA) solution (0.05%/0.02%; Genetix Biotech Asia, New Delhi, India, and Biowest, Nuaillé, France) was used to detach the cells for either subculturing or biological evaluation at a confluence of ∼90%, followed by washing steps and the addition of fresh cell culture media. Cell counting was performed with either a TC20 automated cell counter (Bio-Rad, Berkeley, CA) or a Scepter handheld automated cell counter (60 μM capillary sensor; EMD Millipore, Billerica, MA).

#### Inhibitory Activity against MCT1

A functional 3-BP cytotoxicity assay was conducted as described in the literature^20,39^ with minor modifications. Dilution series of compounds **17**–**32** between 1 nM and 1 μM were prepared in DPBS (Cell Clone, Genetix Biotech Asia, New Delhi India), and 10 μL of each concentration was pipetted into a colorless flat-bottom 96-well microplate (SPL Life Sciences, Gyeonggi-do, Republic of Korea). One hundred and eighty microliters of a cell suspension (5000 cells/well) of A-549 cells was added, and subsequently, 10 μL of 3-BP was added at a concentration of 1 mM (final concentration per well: 50 μM). The plate was kept for 72 h at 37 °C under a 5% CO_2_-humidified atmosphere. Cell viability was determined as described before^43^ by adding 20 μL of a 5 mg/mL solution of MTT to each well and further incubation for 1 h. Eventually, the supernatant was removed and 100 μL of DMSO was added to each well. The absorbance was spectrophotometrically determined at 570 nm using a SpectraMax iD3 multi-mode microplate reader (Molecular Devices, San Jose, CA) with a background correction (690 nm). The determined absorbance values were correlated to the logarithmic concentrations of compounds **17**–**32**, and IC_50_ values were calculated applying nonlinear regression using GraphPad Prism (version 8.4.0., San Diego, CA).

#### Inhibitory Activity against MCT4

Determination of intracellular pH was carried out using the pH-sensitive dye 2′,7′-bis-(2-carboxyethyl)-5/6-carboxyfluorescein acetoxymethyl ester (BCECF-AM; Sigma-Aldrich, St. Louis, MO). Briefly, 90 μL of an MDA-MB-231 cell suspension (50,000 cells/well) was added to a black flat-bottom 96-well microplate (SPL Life Science, Gyeonggi-do, Republic of Korea). The plate was incubated overnight at 37 °C under a 5% CO_2_-humidified atmosphere. Then, 10 μL of the test compounds (10 μM) dissolved in DPBS (Genetix Biotech Asia, New Delhi, India) was added. The plates were further incubated for 12 h at 37 °C under a 5% CO_2_-humidified atmosphere. After incubation, the supernatant was removed and 100 μL of a BCECF-AM (5 μM) solution was added to each well, and the plates were further incubated for 1 h at 37 °C under a 5% CO_2_-humidified atmosphere. Afterward, the dye loading solution was removed and the cells were washed two times with water for injection (Genetix Biotech Asia, New Delhi, India), and subsequently, the intracellular pH was determined. The pH calibration was carried out for each plate by adding 100 μL of intercellular pH calibration buffers for pH 4.5, 5.5, 6.5, and 7.5 (Invitrogen by Thermo Fisher Scientific, Eugene, OR) with 10 μM of nigericin and 10 μM of valinomycin to each well, followed by an incubation period of 30 min at room temperature. Finally, the fluorescence was spectrophotometrically determined (excitation: 430/90 nm; emission: 535 nm) using a SpectraMax iD3 multi-mode microplate reader (Molecular Devices, San Jose, CA). For intracellular pH determination, the fluorescence ratio between the emission wavelengths 430 nM and 490 nm was determined and plotted against the calibration curve.

#### Antiproliferative Activity against MCT1-Expressing Cancer Cell Lines

To determine the growth inhibitory potential of compounds **17**–**32**, 20 μM of each compound either at concentrations between 1 and 100 μM (A-549 or MCF-7) or at 10 and 50 μM (NIH/3T3) was pipetted into colorless flat-bottom 96-well microplates. Thereafter, 180 μL of a cell suspension (5000 cells/well) of either A-549, MCF-7, or NIH/3T3 cells was added and subsequently kept for 72 h at 37 °C under a 5% CO_2_-humidified atmosphere. The cell viability as well as the GI_50_ values were determined as described above.^43^

The glucose deprivation analyses were conducted by adding 4.5 μL of glucose (final concentrations 25% and 50% of the original 4.5 g/L) to each well at an interval of 12 h over a period of 72 h to the 180 μL cell suspension of A-549 cells in a colorless flat-bottom 96-well microplate (SPL Life Sciences, Gyeonggi-do, Republic of Korea). Cell viability was determined as described above.

#### Efficacy of Compounds **24** and **30** against MCT1-Expressing Cells

The ability of the most potent MCT1 inhibitors **24** and **30** to enhance the susceptibility of the MCT1-expressing cell lines A-549^14,15^ toward antineoplastic agent **33** was investigated. For this purpose, 10 μL of compounds **24** or **30** (50 nM, 100 nM, or 500 nM) was transferred onto a colorless flat-bottom 96-well microplate and complemented with 180 μL of a cell suspension containing either approximately 5,000 A-549 cells. A dilution series of compound **33** at concentrations between 50 nM and 50 μM was generated, and 10 μL of each concentration was transferred onto the plate. The observed effects of compounds **24** and **30** were compared to the effect of compound **33** alone on A-549. The plates were incubated for 72 h at 37 °C and a 5% CO_2_-humidified atmosphere, before subsequent processing with the regular MTT assay as described above.^43^

#### Cell Cycle Distribution Analysis of Compound **24**

A standard PI staining procedure was used as described earlier^44−46^ to determine the effect of compounds **24** and **33** on cell cycle progression, followed by flow cytometry analysis (FACS Aria SORP and FACS Aria Fusion, BD Biosciences, Franklin Lakes, NJ). In brief, A-549 cells were seeded onto a 6-well plate (250,000 cells/well) and incubated for 24 h at 37 °C under a 5% CO_2_-humidified atmosphere before being observed under a microscope. The cells were treated with compound **24** (5 μM) or **33** (5 μM) and further incubated for 24 h at 37 °C under a 5% CO_2_-humidified atmosphere. Then, the cells were harvested and washed twice with cold PBS (Genetix Biotech Asia, New Delhi, India) before being fixed overnight in ice-cold 70% (v/v) ethanol at 4 °C. The cells were again washed two times with PBS and subsequently resuspended with RNase (100 μg/mL; HiMedia, Mumbai, India) and their DNA was stained with PI (40 μg/mL; Sigma-Aldrich, Mumbai, India) before being incubated in the dark for 10 min. The DNA content was measured at the Indian Institute of Technology Bombay (IITB, Mumbai, India) by applying flow cytometry.

#### Apoptosis Assay

A PI/Annexin V-FITC double staining method was used to carry out a flow cytometry-based apoptosis assay, as reported earlier.^44,46−51^ For this purpose, a PI/Annexin V-FITC apoptosis detection kit (BD Biosciences, Franklin Lakes, NJ) was used following the manufacturer’s instructions. A-549 cells were seeded onto 6-well plates with a density of 500,000 cells/well and incubated for 24 h at 37 °C under a 5% CO_2_-humidified atmosphere. Subsequently, the cells were exposed to compound **24** (5 μM) followed by a further incubation period of 24 h at 37 °C under a 5% CO_2_-humidified atmosphere. The cells were trypsinized, washed with PBS, and resuspended in 1x binding buffer (1,000,000 cells/mL). One hundred microliters of test solution containing 100,000 cells treated with a mixture of PI (5 μL) and Annexin V-FTIC (5 μL) was incubated in the dark for 10–15 min at room temperature. Four hundred microliters of binding buffer was added, and the cells were resuspended again just before the flow cytometry analysis by using flow cytometry.

#### Inhibitory Activity against Multidrug Transporters ABCB1, ABCC1, and ABCG2

The calcein AM (ABCB1),^2,52−59^ daunorubicin (ABCB1^52,60^ and ABCC1^52,58−60^), and pheophorbide A (ABCG2)^2,52−60^ assays were conducted as reported earlier. Compounds **17**–**32** were pipetted in a volume of 20 μL at a concentration of 100 μM onto a clear 96-well flat-bottom plate (Brand, Wertheim, Germany), adding 160 μL of cell suspension containing either 30,000 cells/well (calcein AM) or 45,000 cells/well (daunorubicin and pheophorbide A) in either phenol red-free RPMI-1640 (A2780/ADR and H69AR) or phenol red-free DMEM (MDCK II BCRP) without further supplements. Compounds **17**–**32** were incubated with the cells for 30 min before adding the respective fluorescence dye [20 μL of calcein AM (3.125 μM), daunorubicin (30 μM), or pheophorbide A (5 μM); final concentrations: calcein AM: 0.3125 μM; daunorubicin: 3 μM; pheophorbide A: 0.5 μM] to each well. The fluorescence increase was measured over a time period of 30 min in 30 s intervals in the case of calcein AM (excitation: 485 nm; emission: 520 nm) using a Paradigm microplate reader (Beckman Coulter, Brea, CA), while the average fluorescence value per well was measured after an incubation period of 180 min and 120 min in the case of daunorubicin and pheophorbide A, respectively (excitation: 488 nm; emission: 695/50 nm), using an Attune NxT flow cytometer (Invitrogen, Waltham, MA). The slopes (calcein AM) or average fluorescence values (daunorubicin and pheophorbide A) per well were calculated and compared to the standard inhibitors **34**–**36**. For compounds with an inhibitory power over 50% against a single target or 20% (+SEM) against all three ABC transporters, full-blown concentration-effect curves between 100 nM and 100 μM final compound concentrations have been generated. The data was processed using GraphPad Prism version 8.4.0, and IC_50_ values were determined, applying three- or four-parameter logistic equations, whatever was statistically preferred.

#### Efficacy of Compounds **24** and **30** against ABCB1-expressing Cells

An MDR-reversal assay^2,55−59^ was used to investigate the ability to reverse ABCB1-mediated MDR in A2780/ADR cells toward the cytotoxic ABCB1 substrate **33**. For this purpose, 20 μL of the most potent ABCB1 inhibitors **24** or **30** (1 μM, 5 μM, 10 μM or 50 μM) was transferred onto clear 96-well flat-bottom plates and complemented with 160 μL of a cell suspension containing approximately 10,000 A2780/ADR cells. A dilution series of **33** at concentrations between 0.1 μM and 100 μM was generated, and 20 μL of each concentration was transferred onto the plates (final concentration: 0.01–10 μM). The plates were incubated for 72 h at 37 °C and a 5% CO_2_-humidified atmosphere, before subsequent processing with the regular MTT assay as described above. The observed effects of compounds **24** and **30** were compared to the effect of **33** alone on A2780/ADR cells as well as its sensitive counterpart A2780 (∼10,000 cells/well).

### Computational Analyses

#### Molecular Docking of Lead MCT1 Inhibitors

Molecular docking was conducted as described earlier^82−85^ by applying Chimera version 1.10.2 with the Autodock vina 1.1.2 software in conjunction with the PyRx Virtual Screening Tool 0.8 and the Biovia Discovery studio.^86^ The reference MCT1 inhibitors **7** and **8**, as well as the most potent lead MCT1 inhibitors **24** and **30**, were visualized by applying ChemDraw professional 17.1 and stored in mol file format. Energy minimization was accomplished with the Universal Force Field (UFF) within the PyRx.^87^ For molecular docking of compounds **24** and **30**, a recently released cryo-EM structure of MCT1 in complex with basigin-2 (PDB ID: 7CKR)^19^ bound to the reference MCT1 inhibitor **7** was used, and the information deduced from its binding mode and binding affinity was used for subsequent analyses. A three-dimensional grid box (*x* = 67.52 Å; *y* = 62.25 Å; *z* = 66.43 Å) was adjusted, and the exhaustiveness parameter was set to 8.

#### Determination of Molecular–Structural and Physicochemical Properties

The numbers of H-bond donors, H-bond acceptors, and rotatable bonds as well as the physicochemical properties CLog *P*, MW, MR, and TPSA were calculated by applying the online web service SwissADME (http://www.swissadme.ch).^75^ CLog *P* was determined by using the atomistic method,^76^ while TPSA was determined using the fragment-based method.^77^

## Supporting Information Available

Molecular formula strings and the biological data (CSV)
Analytical data [FTIR, ^1^H NMR, MS, and HPLC data of compounds **17–32**] (PDF)

## Accession Codes

Computational data; PDB accession code: 7CKR.

## Abbreviations Used

ABC: ATP-binding cassette
ATP: adenosine triphosphate
BCRP: breast cancer resistance protein (ABCG2)
3-BP: 3-bromopyruvate
CHC: hydroxy-4-cyanocinnamic acid
CLog *P*: calculated octanol/water partition coefficient
FITC: fluorescein isothiocyanate
GI_50_: half-maximal growth inhibition concentration
GLUT1: glucose transporter 1
HIF-1: hypoxia-inducible factor-1
IC_50_: half-maximal inhibition concentration
LDH: lactate dehydrogenase
MCP1: mitochondrial pyruvate carrier 1
MCT1: monocarboxylate transporter 1
MDR: multidrug resistance
MRP1: multidrug resistance-associated protein 1 (ABCC1)
MR: molar refractivity
MTT: 3-(4,5-dimethylthiazol-2-yl)-2,5-diphenyltetrazolium bromide
MW: molecular weight
P-gp: P-glycoprotein (ABCB1)
PI: propidium iodide
SEM: standard error of the mean
SLC: solute carrier
SAR: structure–activity relationship
TCA: tricarboxylic acid cycle
TPSA: topological polar surface area
