# Supplementary material for: Indole Derivatives as New Structural Class of Potent and Antiproliferative Inhibitors of Monocarboxylate Transporter 1 (MCT1; SLC16A1)
Source: J Med Chem. 2022 Dec 30;66(1):657–76. doi: 10.1021/acs.jmedchem.2c01612 (PMC9841531; doi:10.1021/acs.jmedchem.2c01612)
Supplement: Supplementary file 2 — jm2c01612_si_002.pdf [file jm2c01612_si_002.pdf]

Indole Derivatives as New Structural Class of Potent and Antiproliferative Inhibitors of Monocarboxylate Transporter 1 (MCT1; SLC16A1)

Sachin Puri[a,†], *Katja Stefan*[b,†], *Sharuk L. Khan*[c], *Jens Pahnke*[b,d,e], *Sven Marcel Stefan*[b,d,*], *Kapil Juvale*[a,*]

[a] Shobhaben Pratapbhai Patel School of Pharmacy & Technology Management, SVKM's NMIMS, V.L. Mehta Road, Vile Parle (W), Mumbai, 400056, India

[b] Department of Pathology, Section of Neuropathology, Translational Neurodegeneration Research and Neuropathology Lab (www.pahnkelab.eu), University of Oslo and Oslo University Hospital, Sognsvannsveien 20, 0372 Oslo, Norway

[c] Department of Pharmaceutical Chemistry, N.B.S. Institute of Pharmacy, Ausa 413520, Maharashtra, India

[d] Drug Development and Chemical Biology Lab, Lübeck Institute of Experimental Dermatology (LIED), University of Lübeck and University Medical Center Schleswig-Holstein, Ratzeburger Allee 160, 23538 Lübeck, Germany

[e] Department of Pharmacology, Faculty of Medicine, University of Latvia, Jelgavas iela 4, 1004 Rīga, Latvia

† these authors contributed equally to this work

* Corresponding authors: Kapil Juvale (kjuvale@gmail.com; kapil.juvale@nmims.edu)

Sven Marcel Stefan (s.m.stefan@medisin.uio.no;

svenmarcel.stefan@uksh.de)

Supporting Information

FTIR, ^{1}H NMR, MS, and HPLC data of compounds **17–32** (pdf)

**Figure S1**	FTIR spectrum of compound **17**	S5
**Figure S2**	^{1}H-NMR spectrum of compound **17**	S5
**Figure S3**	MS spectrum of compound **17**	S6
**Figure S4**	HPLC chromatogram of compound **17**	S6
**Figure S5**	FTIR spectrum of compound **18**	S7
**Figure S6**	^{1}H-NMR spectrum of compound **18**	S7
**Figure S7**	MS spectrum of compound **18**	S8
**Figure S8**	HPLC chromatogram of compound **18**	S8
**Figure S9**	FTIR spectrum of compound **19**	S9
**Figure S10**	^{1}H-NMR spectrum of compound **19**	S9
**Figure S11**	MS spectrum of compound **19**	S10
**Figure S12**	HPLC chromatogram of compound **19**	S10
**Figure S13**	FTIR spectrum of compound **20**	S11
**Figure S14**	^{1}H-NMR spectrum of compound **20**	S11
**Figure S15**	MS spectrum of compound **20**	S12
**Figure S16**	HPLC chromatogram of compound **20**	S12
**Figure S17**	FTIR spectrum of compound **21**	S13
**Figure S18**	^{1}H-NMR spectrum of compound **21**	S13
**Figure S19**	MS spectrum of compound **21**	S14
**Figure S20**	HPLC chromatogram of compound **21**	S14
**Figure S21**	FTIR spectrum of compound **22**	S15
**Figure S22**	^{1}H-NMR spectrum of compound **22**	S15
**Figure S23**	MS spectrum of compound **22**	S16
**Figure S24**	HPLC chromatogram of compound **22**	S16

**Figure S25**	FTIR spectrum of compound **23**	S17
**Figure S26**	^{1}H-NMR spectrum of compound **23**	S17
**Figure S27**	MS spectrum of compound **23**	S18
**Figure S28**	HPLC chromatogram of compound **23**	S18
**Figure S29**	FTIR spectrum of compound **24**	S19
**Figure S30**	^{1}H-NMR spectrum of compound **24**	S19
**Figure S31**	MS spectrum of compound **24**	S20
**Figure S32**	HPLC chromatogram of compound **24**	S20
**Figure S33**	FTIR spectrum of compound **25**	S21
**Figure S34**	^{1}H-NMR spectrum of compound **25**	S21
**Figure S35**	MS spectrum of compound **25**	S22
**Figure S36**	HPLC chromatogram of compound **25**	S22
**Figure S37**	FTIR spectrum of compound **26**	S23
**Figure S38**	^{1}H-NMR spectrum of compound **26**	S23
**Figure S39**	MS spectrum of compound **26**	S24
**Figure S40**	HPLC chromatogram of compound **26**	S24
**Figure S41**	FTIR spectrum of compound **27**	S25
**Figure S42**	^{1}H-NMR spectrum of compound **27**	S25
**Figure S43**	MS spectrum of compound **27**	S26
**Figure S44**	HPLC chromatogram of compound **27**	S26
**Figure S45**	FTIR spectrum of compound **28**	S27
**Figure S46**	^{1}H-NMR spectrum of compound **28**	S27
**Figure S47**	MS spectrum of compound **28**	S28
**Figure S48**	HPLC chromatogram of compound **28**	S28
**Figure S49**	FTIR spectrum of compound **29**	S29
**Figure S50**	^{1}H-NMR spectrum of compound **29**	S29
**Figure S51**	MS spectrum of compound **29**	S30
**Figure S52**	HPLC chromatogram of compound **29**	S30

**Figure S53**	FTIR spectrum of compound **30**	S31
**Figure S54**	^{1}H-NMR spectrum of compound **30**	S31
**Figure S55**	MS spectrum of compound **30**	S32
**Figure S56**	HPLC chromatogram of compound **30**	S32
**Figure S57**	FTIR spectrum of compound **31**	S33
**Figure S58**	^{1}H-NMR spectrum of compound **31**	S33
**Figure S59**	MS spectrum of compound **31**	S34
**Figure S60**	HPLC chromatogram of compound **31**	S34
**Figure S61**	FTIR spectrum of compound **32**	S35
**Figure S62**	^{1}H-NMR spectrum of compound **32**	S35
**Figure S63**	MS spectrum of compound **32**	S36
**Figure S64**	HPLC chromatogram of compound **32**	S36

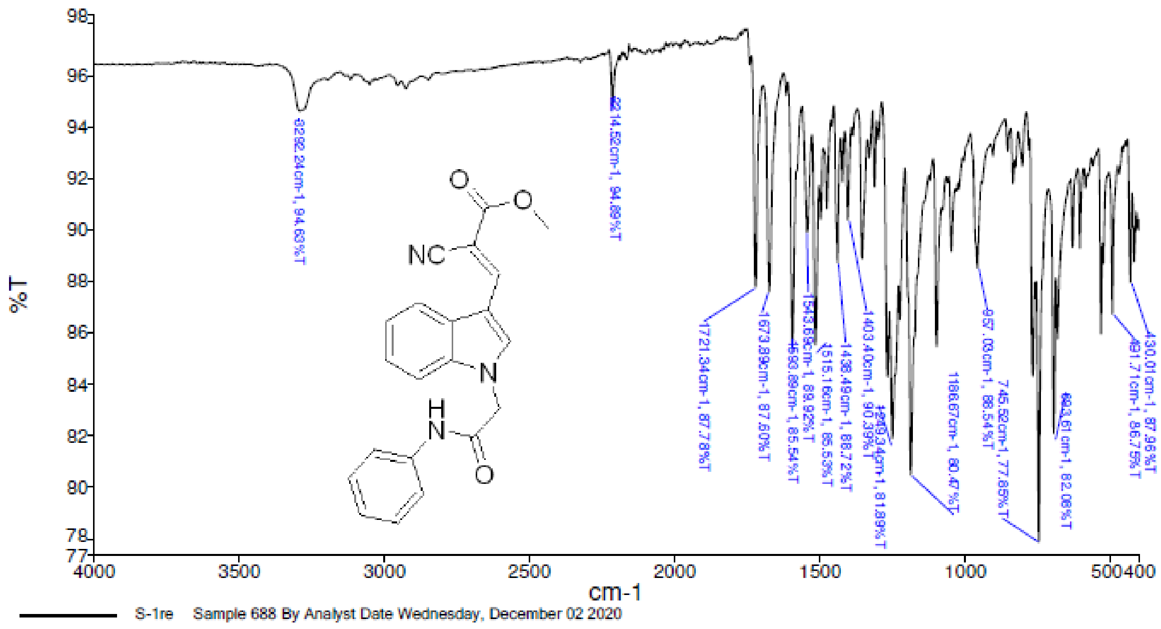

Figure S1 FTIR spectrum of compound **17**.

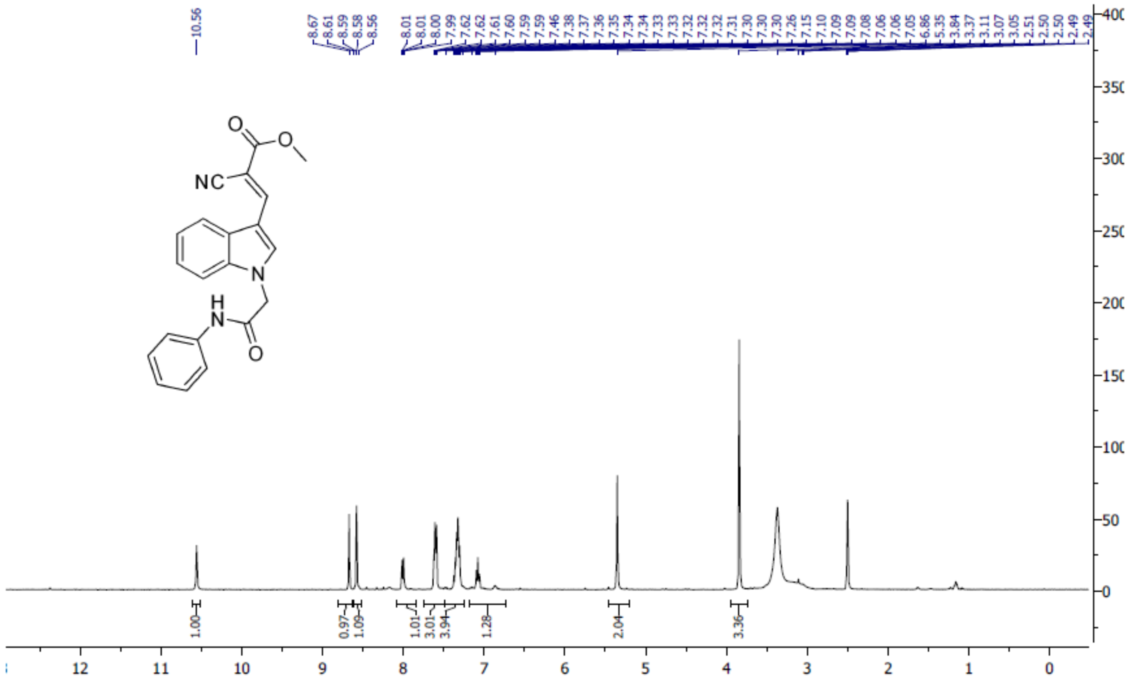

Figure S2 ^{1}H NMR spectrum of compound **17**.

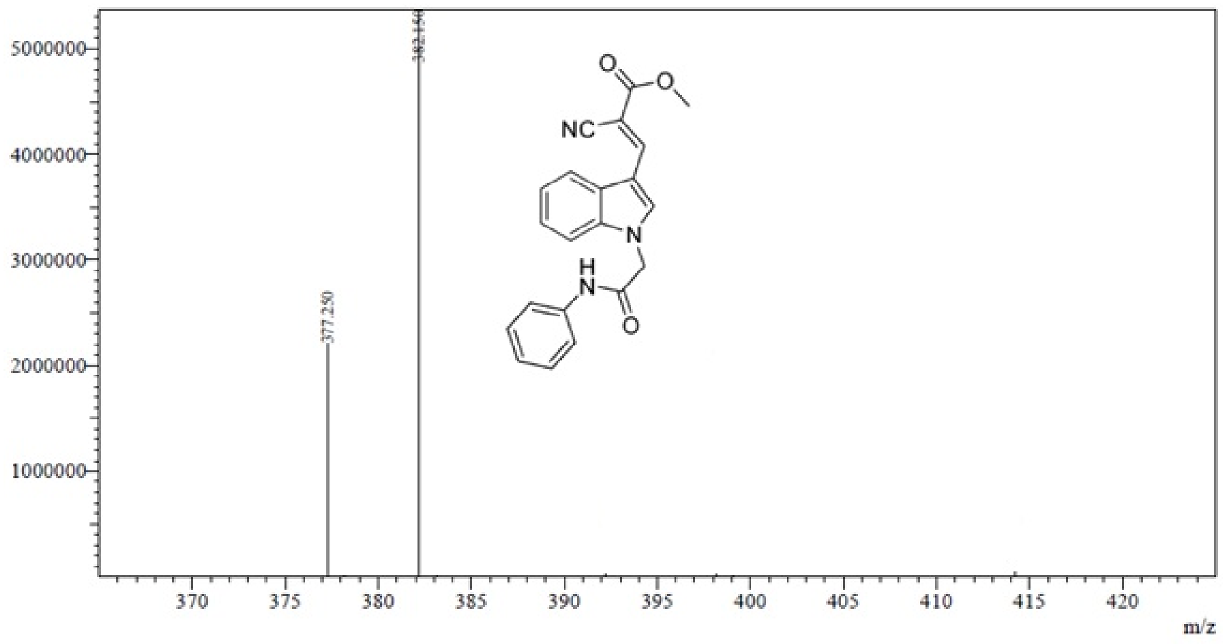

Figure S3 MS spectrum of compound **17**.

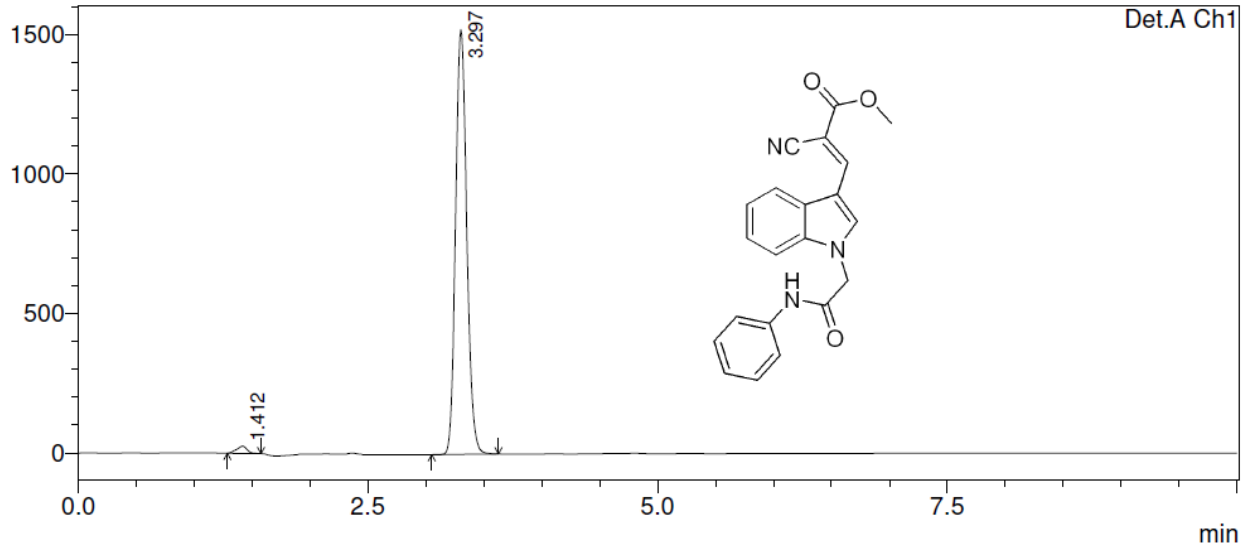

PeakTable

Detector A Ch1 254nm

Peak#	Ret. Time	Area	Height	Area %	Height %	Tailing Factor
1	1.412	167625	26574	1.618	1.714	0.969
2	3.297	10190335	1523997	98.382	98.286	1.183
Total		10357960	1550571	100.000	100.000	

Figure S4 HPLC chromatogram of compound **17**.

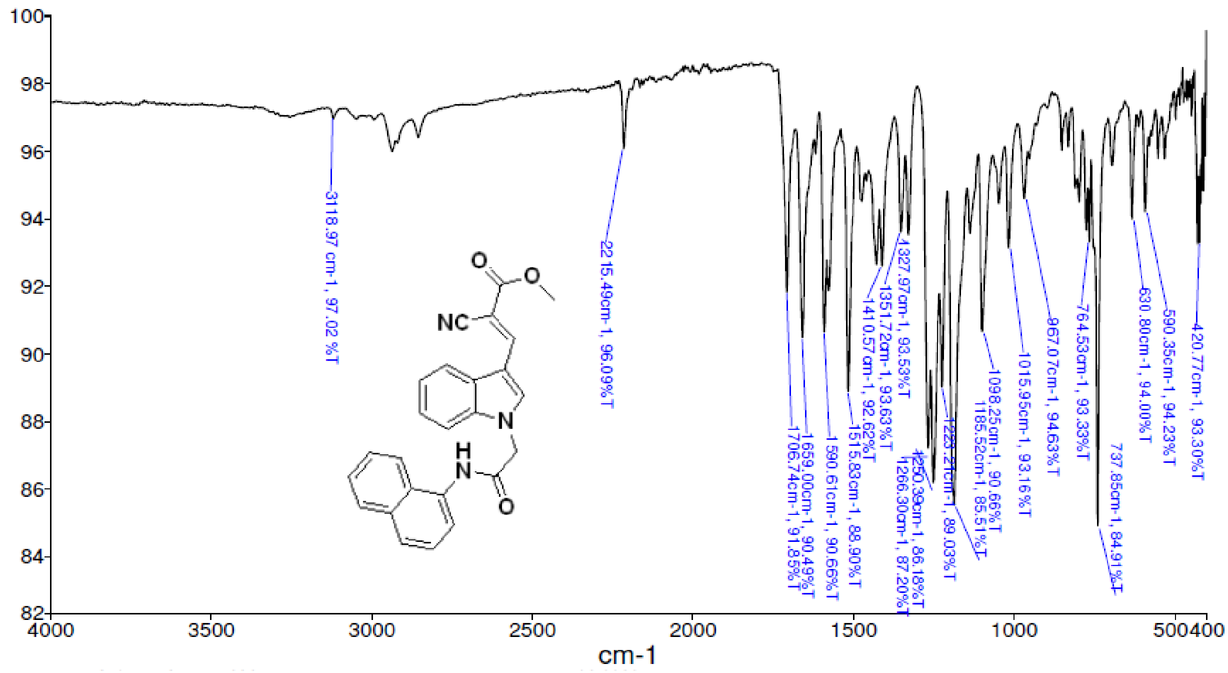

Figure S5 FTIR spectrum of compound **18**.

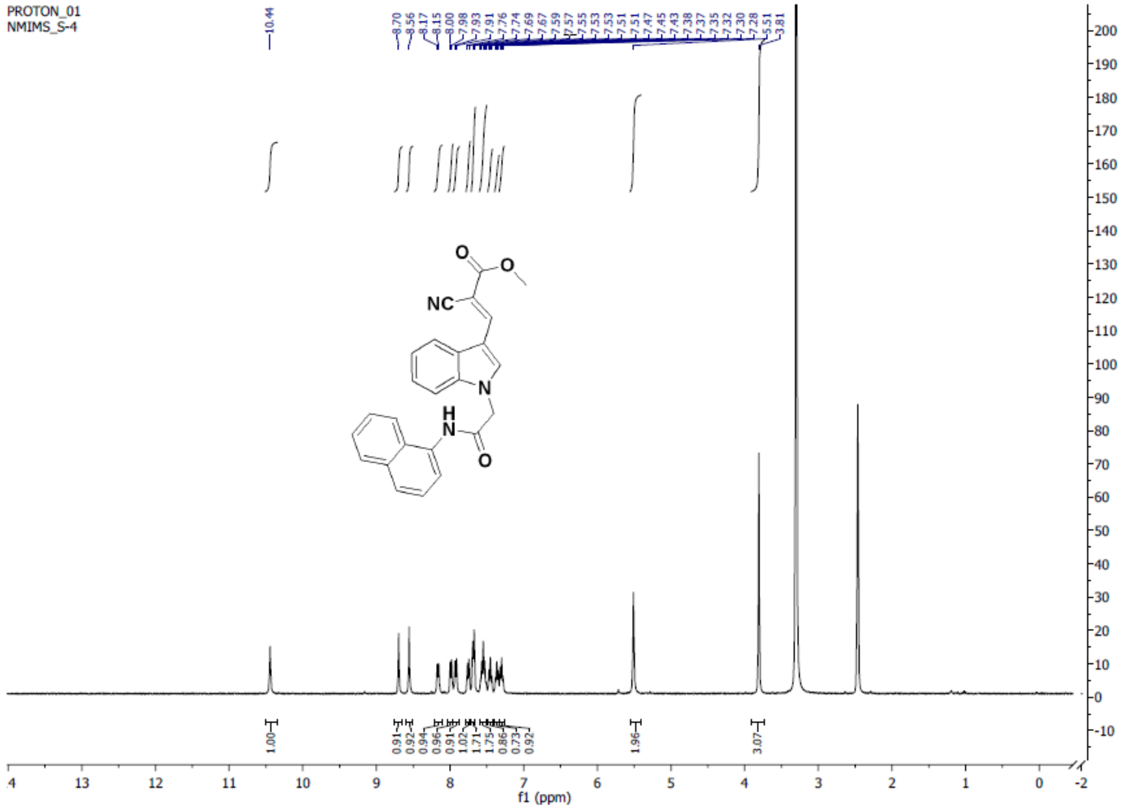

Figure S6 ^{1}H NMR spectrum of compound **18**.

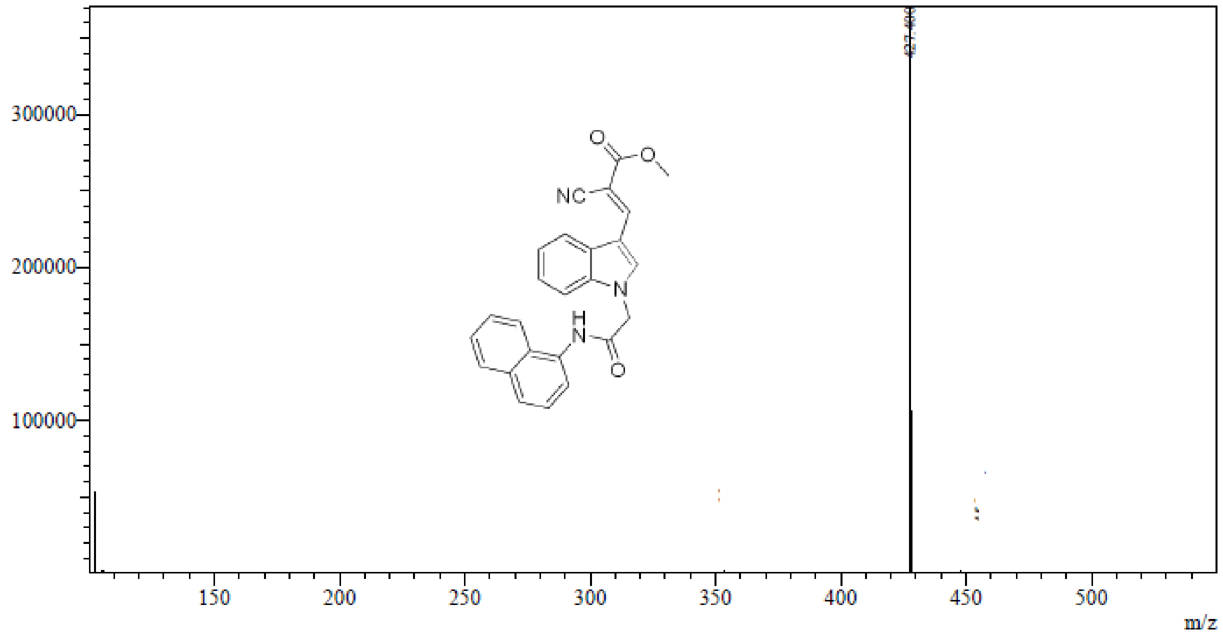

Figure S7 MS spectrum of compound **18**.

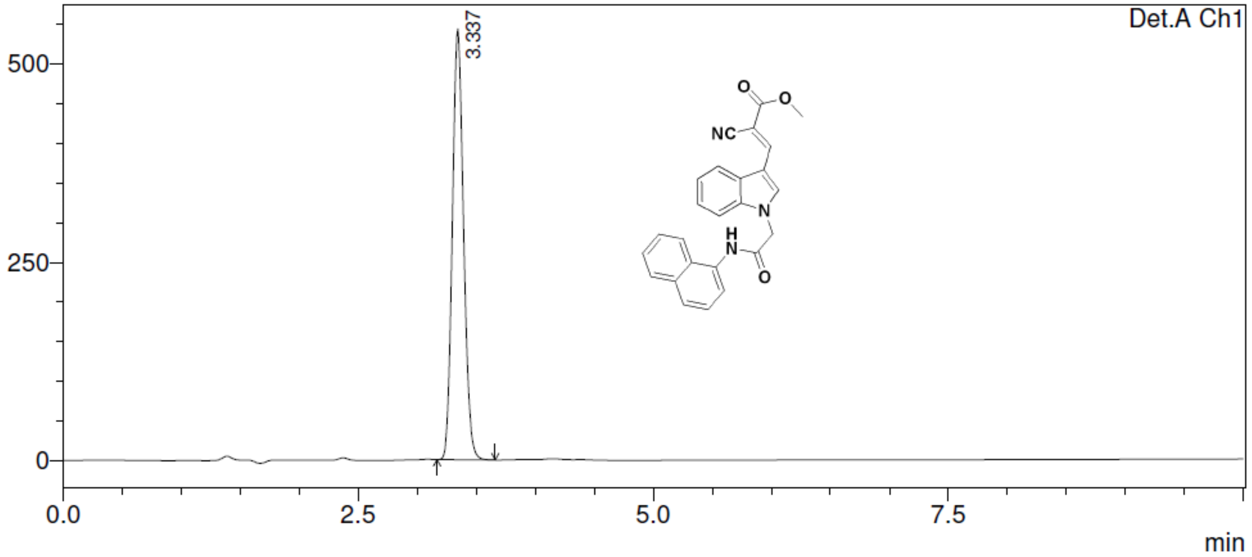

PeakTable

Detector A Ch1 254nm

Peak#	Ret. Time	Area	Height	Area %	Height %
1	3.337	3602721	543240	100.000	100.000
Total		3602721	543240	100.000	100.000

Figure S8 HPLC chromatogram of compound **18**.

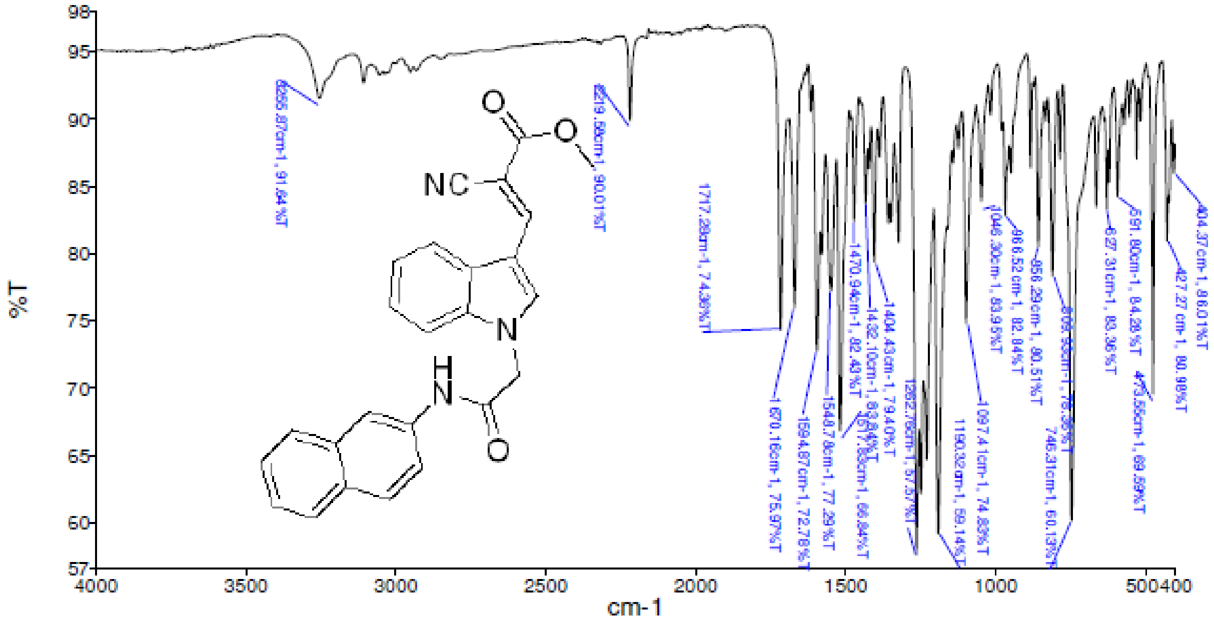

Figure S9 FTIR spectrum of compound **19**.

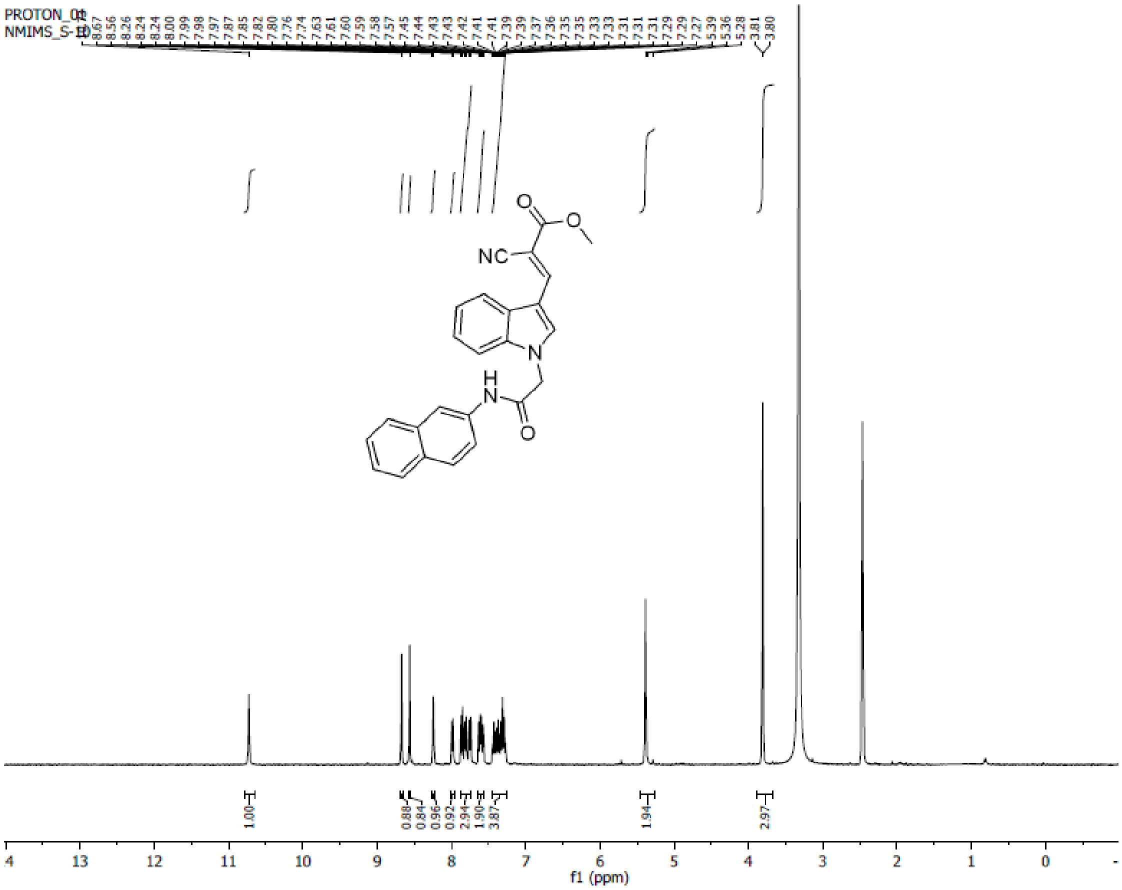

FigureS10 ^{1}H NMR spectrum of compound **19**.

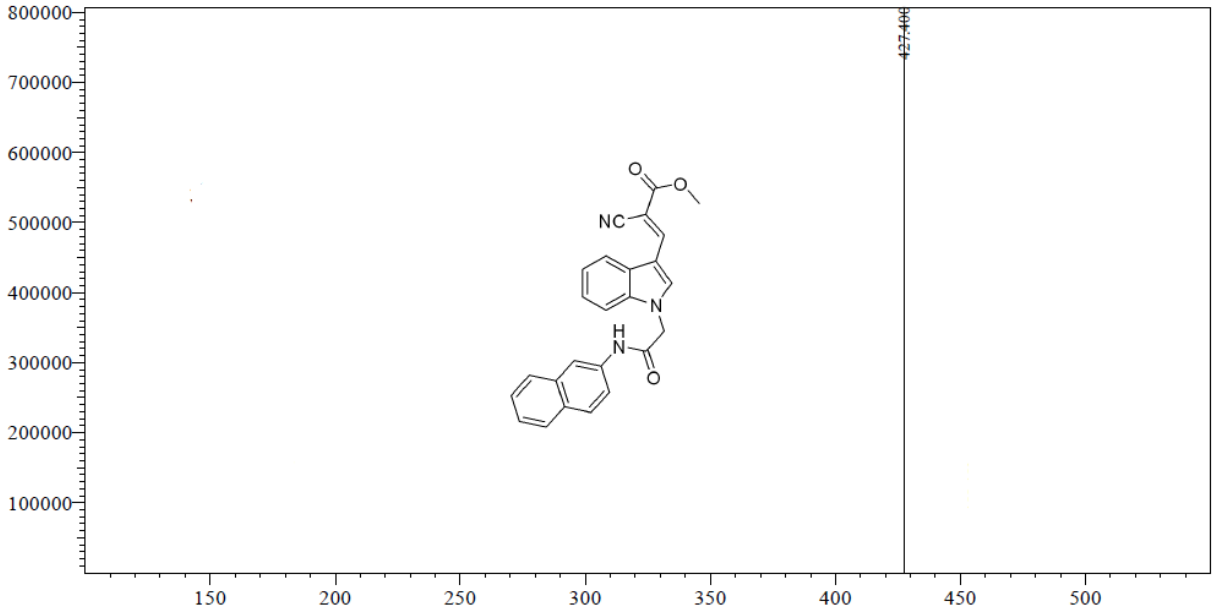

Figure 11 MS spectrum of compound **19**.

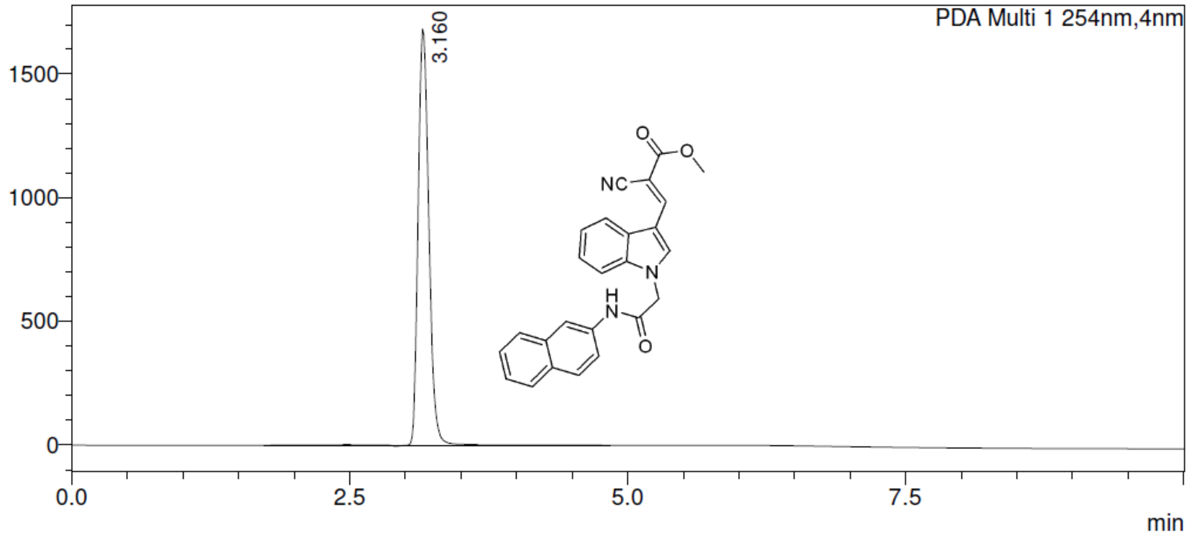

PDA Ch1 254nm

Peak#	Ret. Time	Area	Height	Area%	Height%
1	3.160	11281638	1681904	100.000	100.000
Total		11281638	1681904	100.000	100.000

Figure S12 HPLC chromatogram of compound **19**.

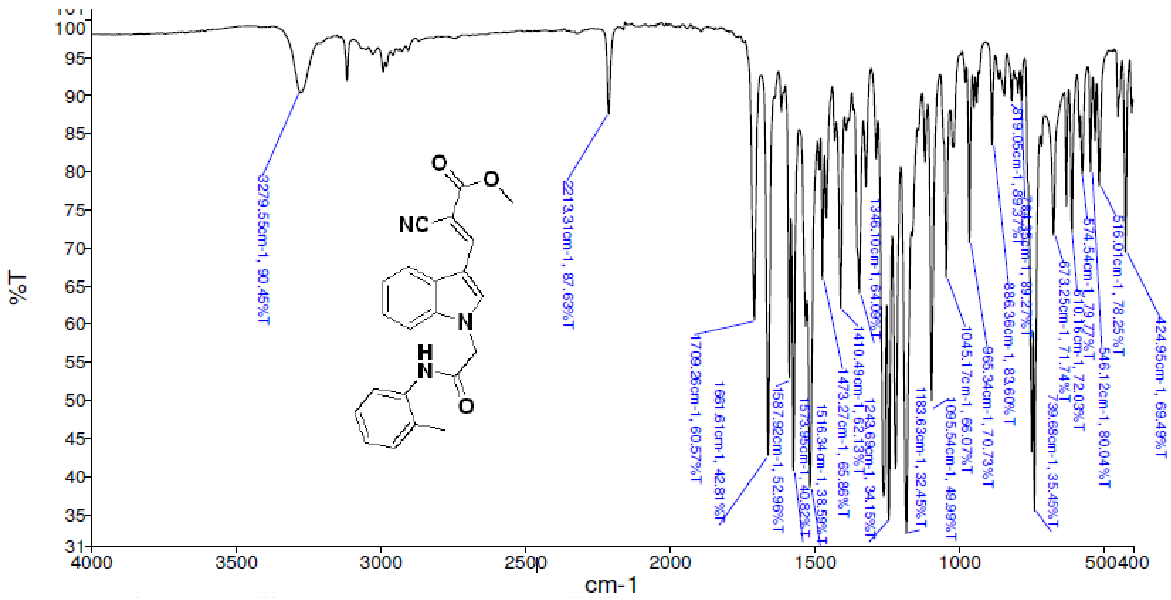

Figure S13 FTIR spectrum of compound **20**.

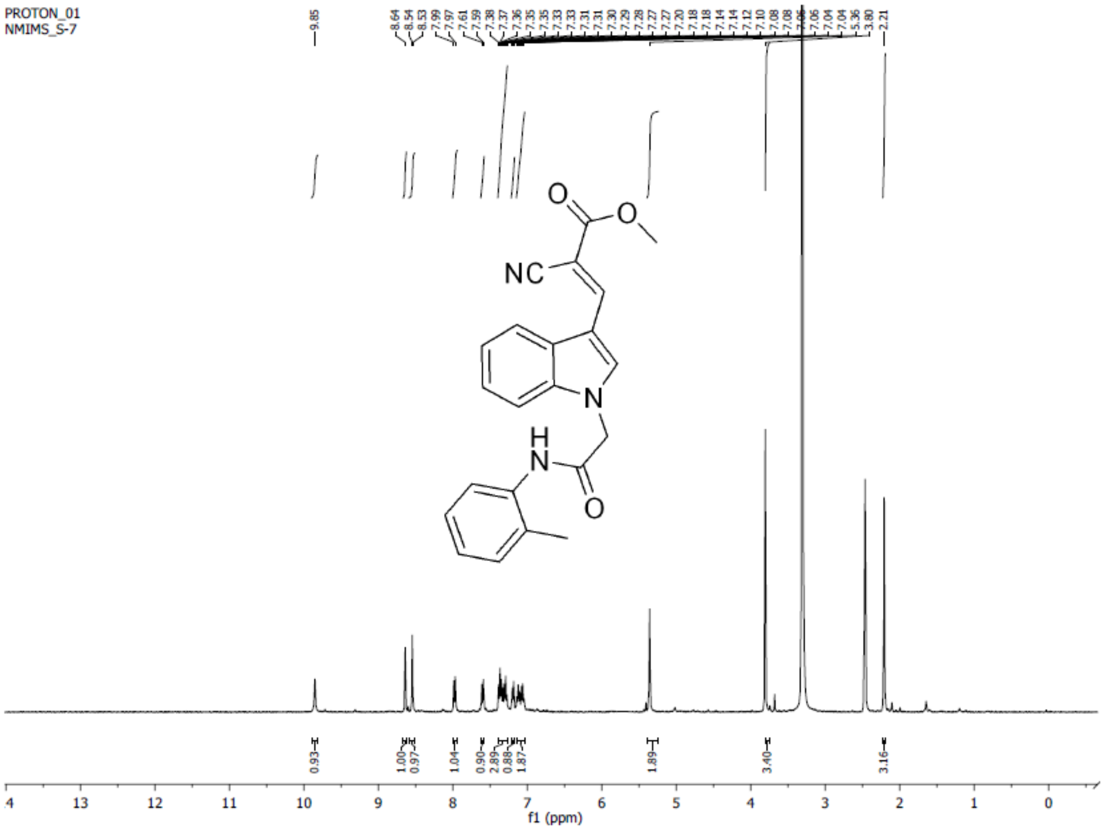

Figure S14 ^{1}H NMR spectrum of compound **20**.

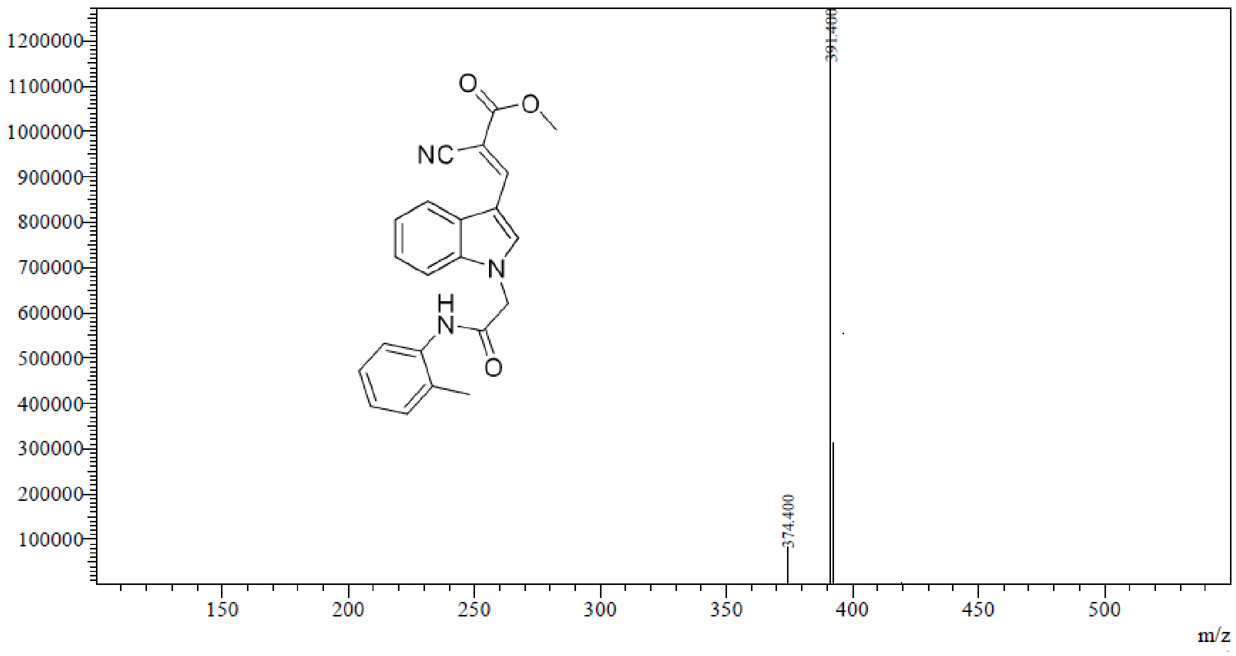

Figure S15 MS spectrum of compound **20**.

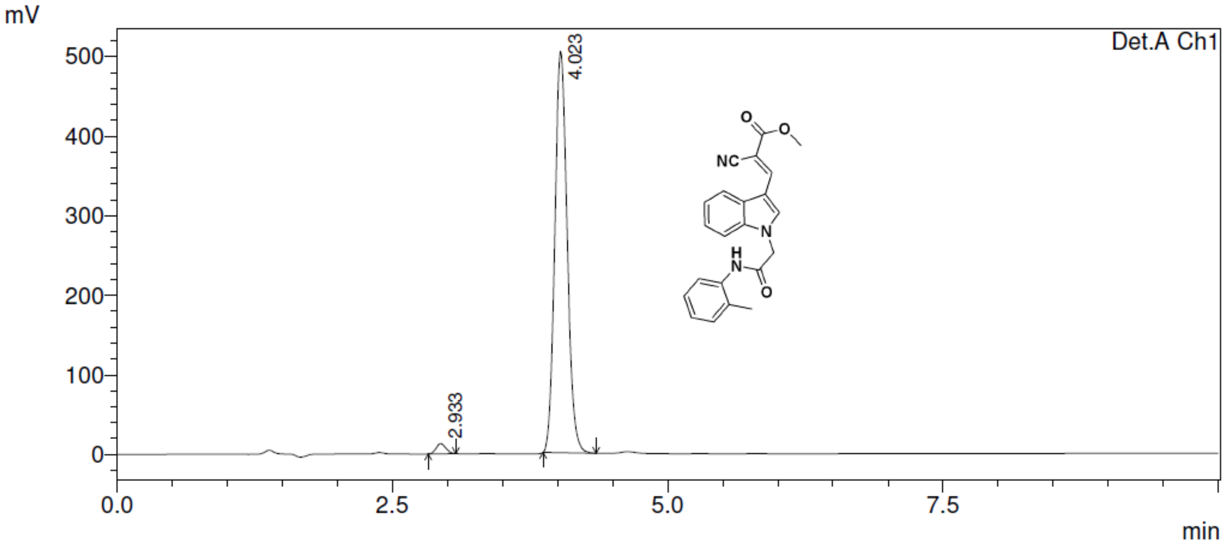

PeakTable

Detector A Ch1 254nm

Peak#	Ret. Time	Area	Height	Area %	Height %
1	2.933	79078	12806	1.972	2.476
2	4.023	3931133	504390	98.028	97.524
Total		4010211	517196	100.000	100.000

Figure S16 HPLC chromatogram of compound **20**.

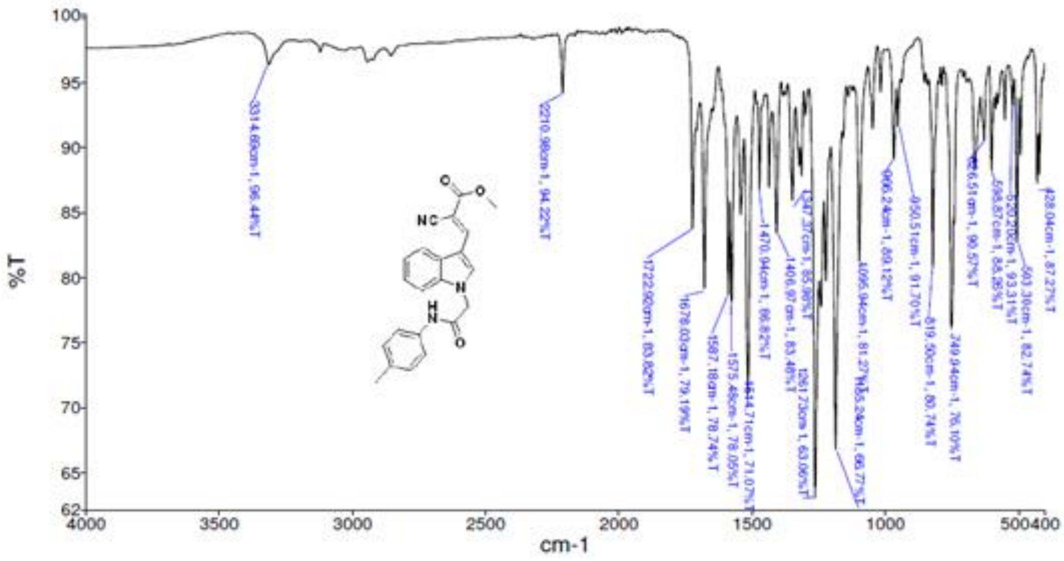

Figure S17 FTIR spectrum of compound **21**.

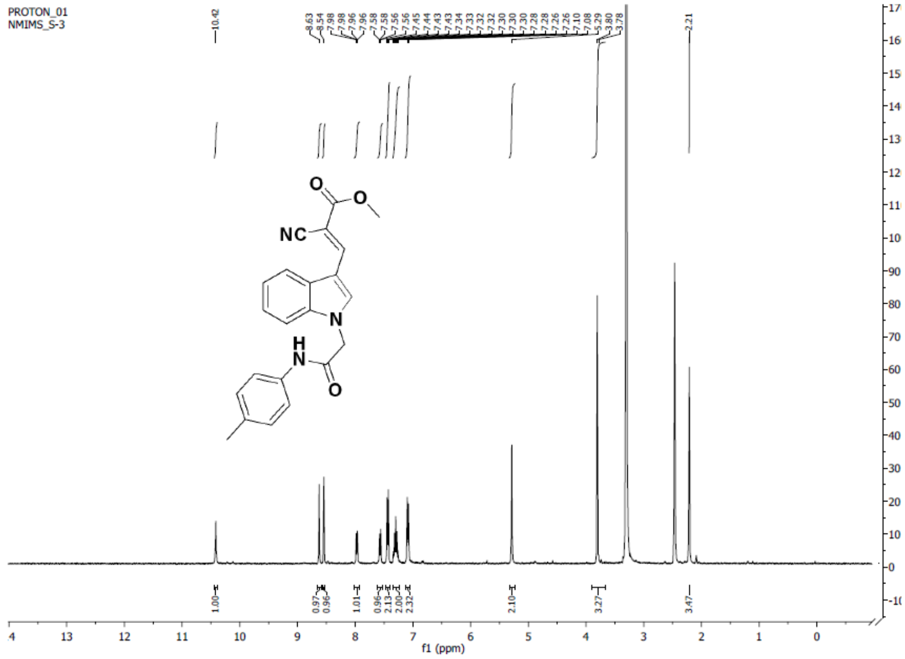

Figure S18 ^{1}H NMR spectrum of compound **21**

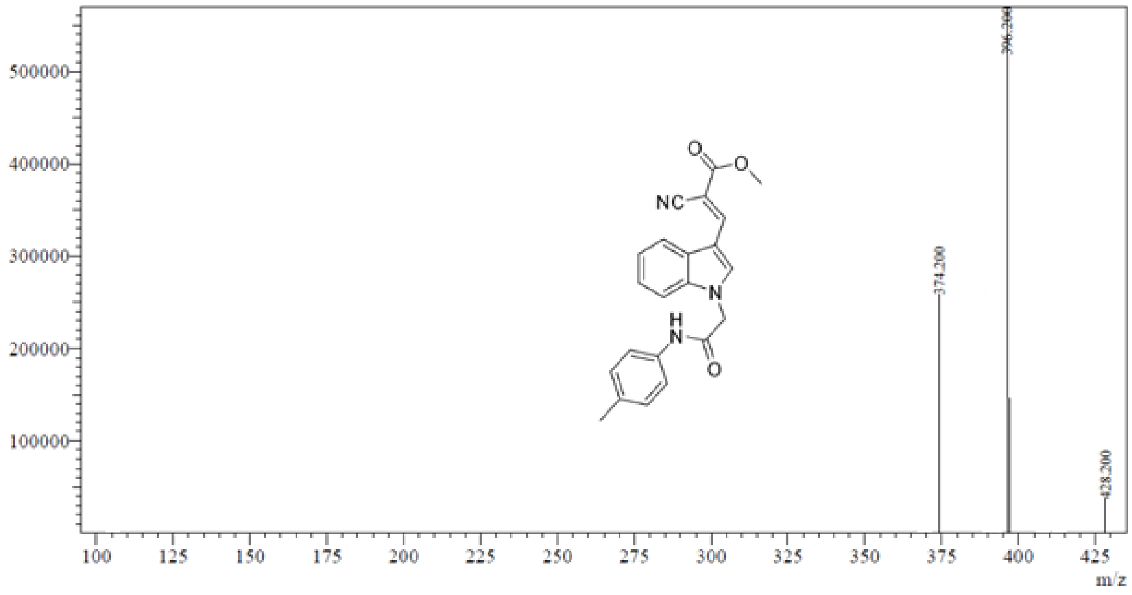

Figure S19 MS spectrum of compound **21**.

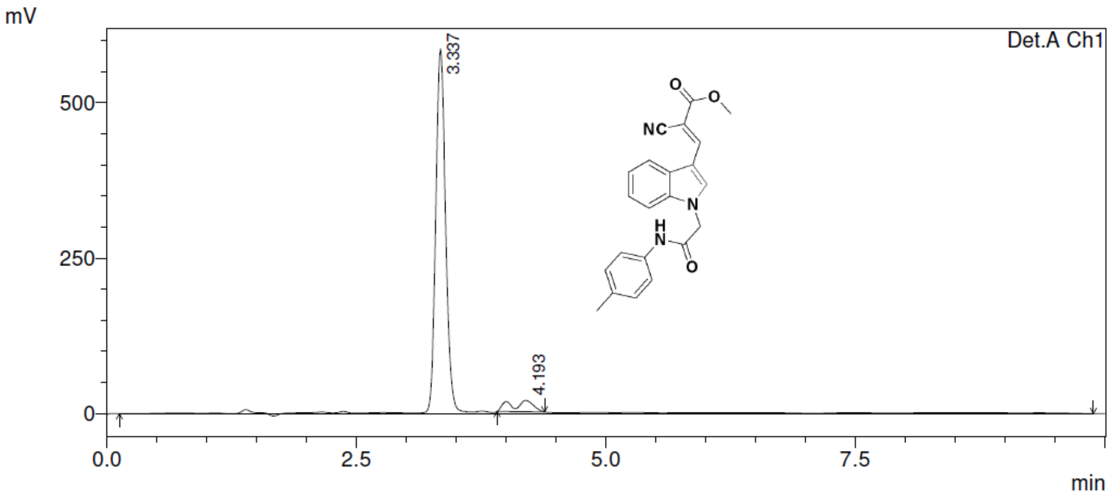

PeakTable

Detector A Ch1 254nm

Peak#	Ret. Time	Area	Height	Area %	Height %
1	3.337	4799500	587001	94.604	97.000
2	4.193	273764	18152	5.396	3.000
Total		5073264	605154	100.000	100.000

Figure S20 HPLC chromatogram of compound **21**.

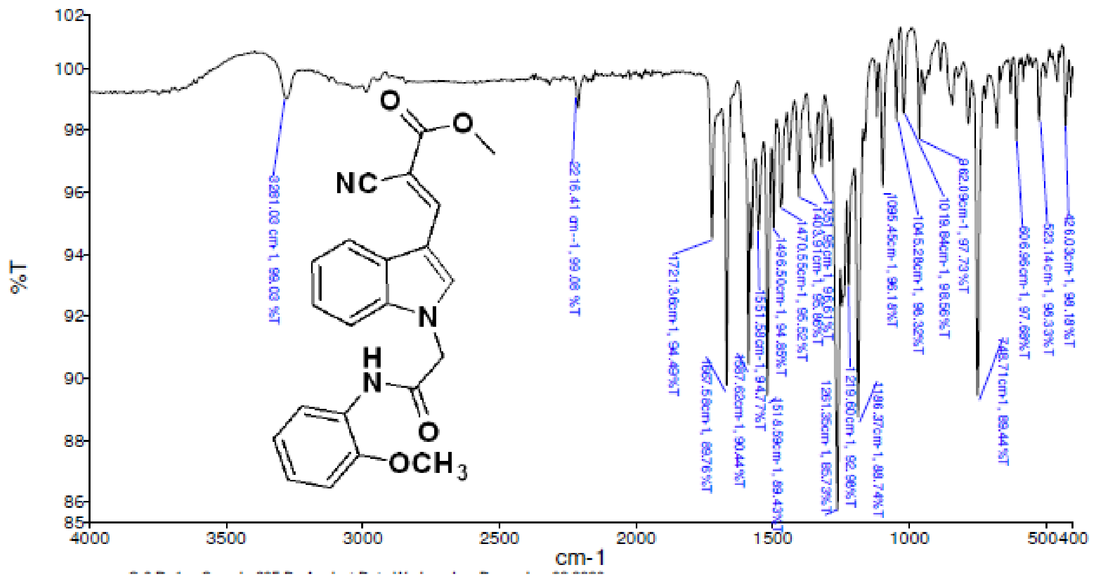

Figure S21 FTIR spectrum of compound **22**.

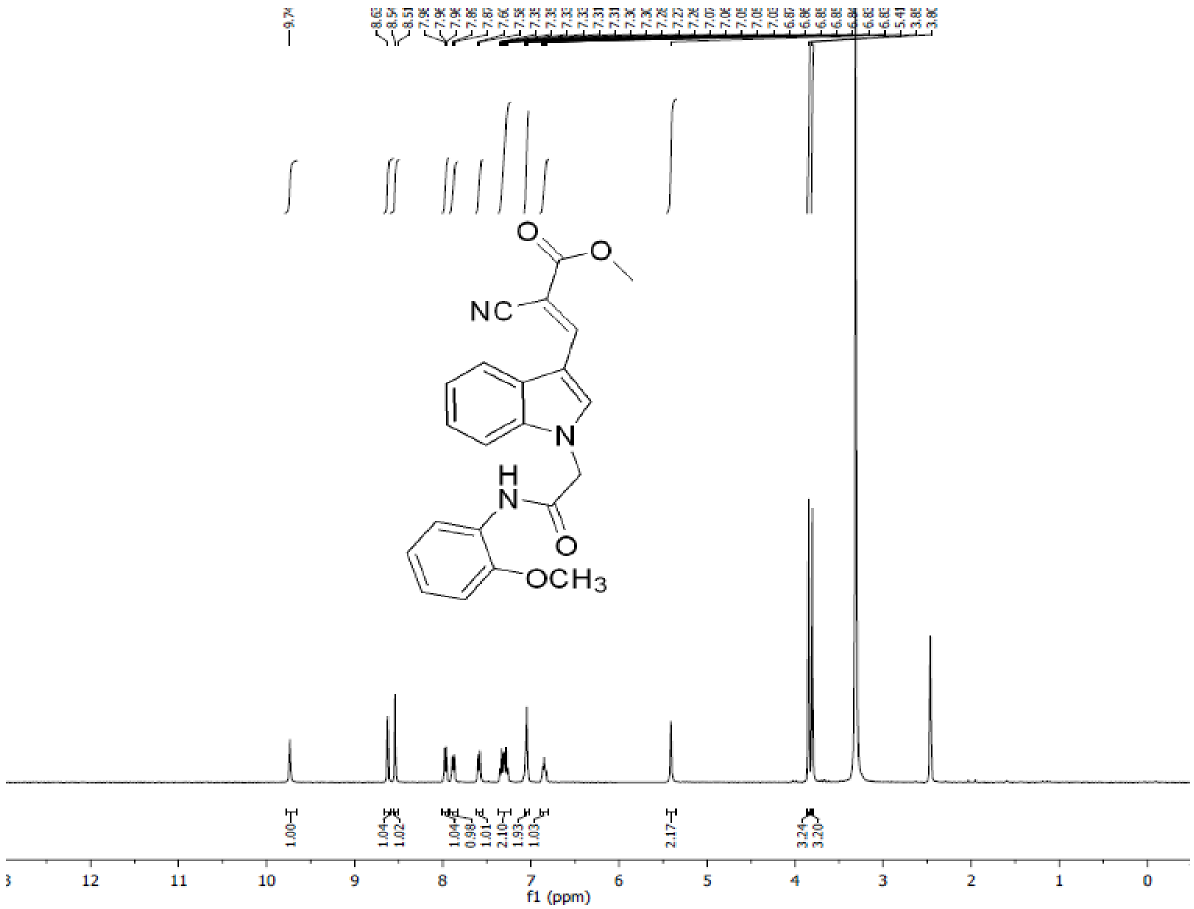

Figure S22 ^{1}H NMR spectrum of compound **22**.

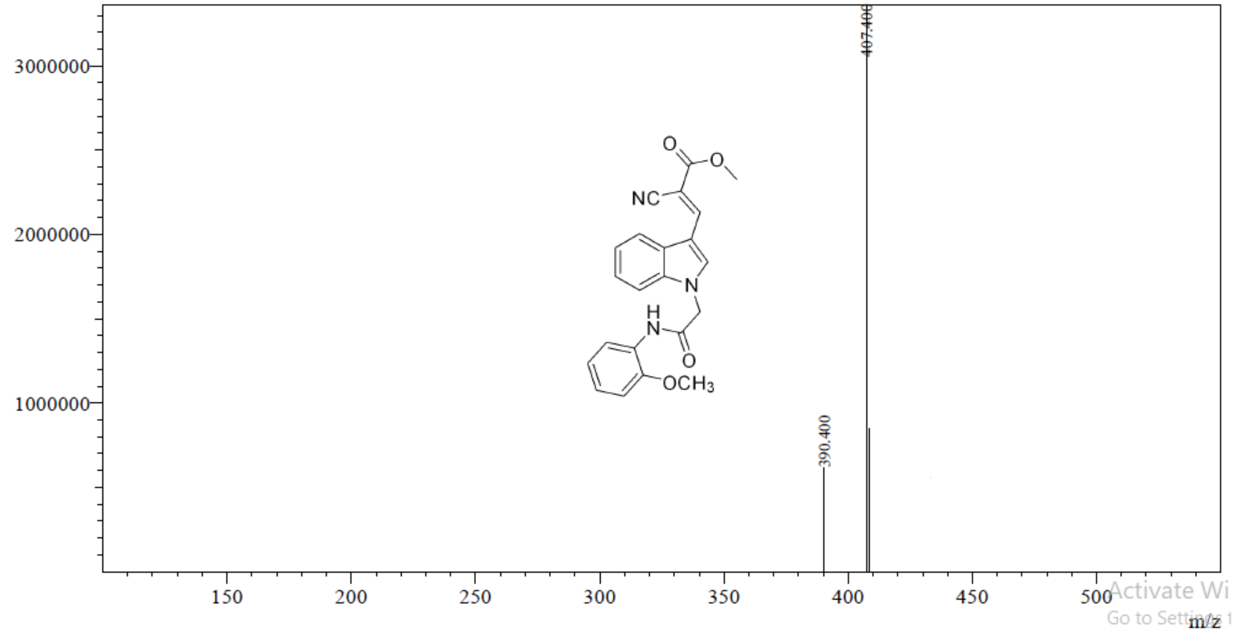

Figure S23 MS spectrum of compound **22**.

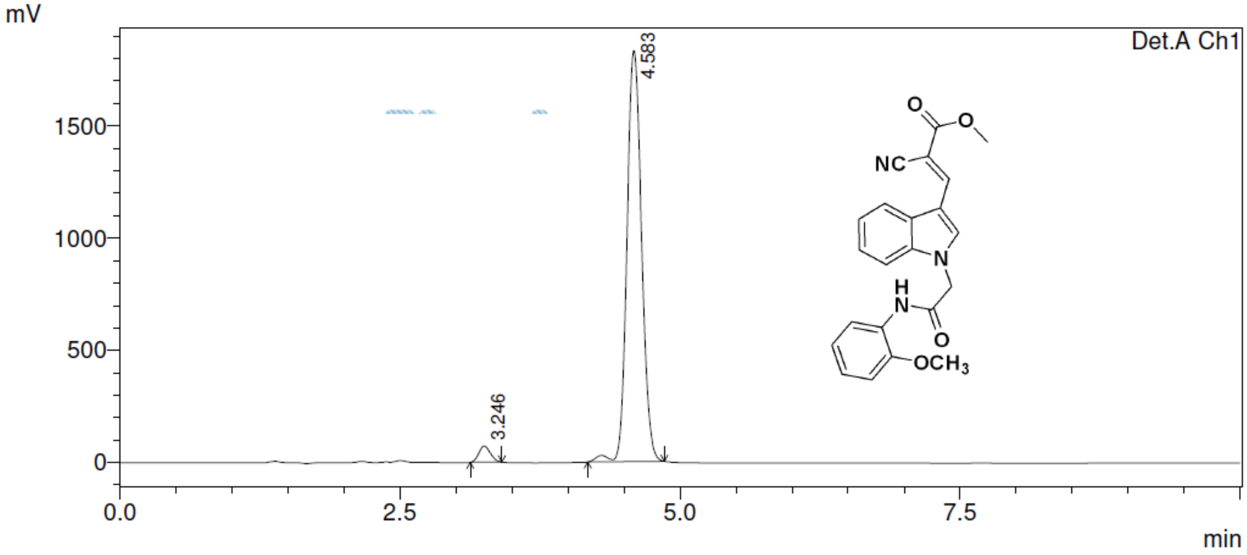

PeakTable

Detector A Ch1 254nm

Peak#	Ret. Time	Area	Height	Area %	Height %
1	3.246	518301	72410	3.013	3.807
2	4.583	16684296	1829417	96.987	96.193
Total		17202597	1901827	100.000	100.000

Figure S24 HPLC chromatogram of compound **22**.

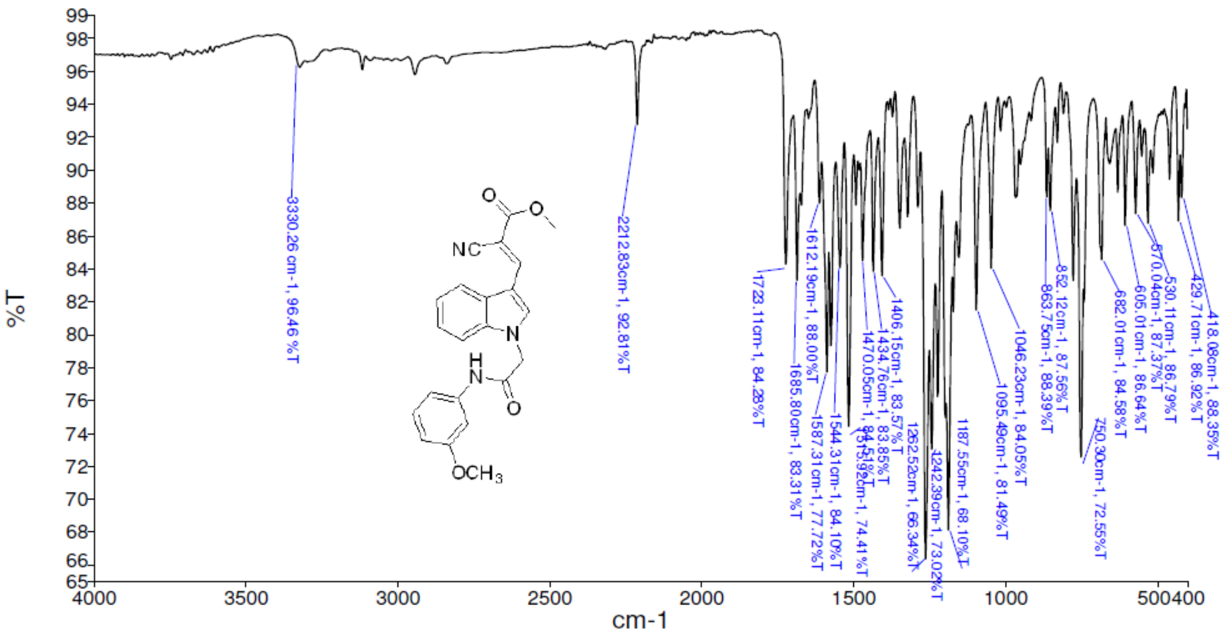

Figure S25 FTIR spectrum of compound **23**.

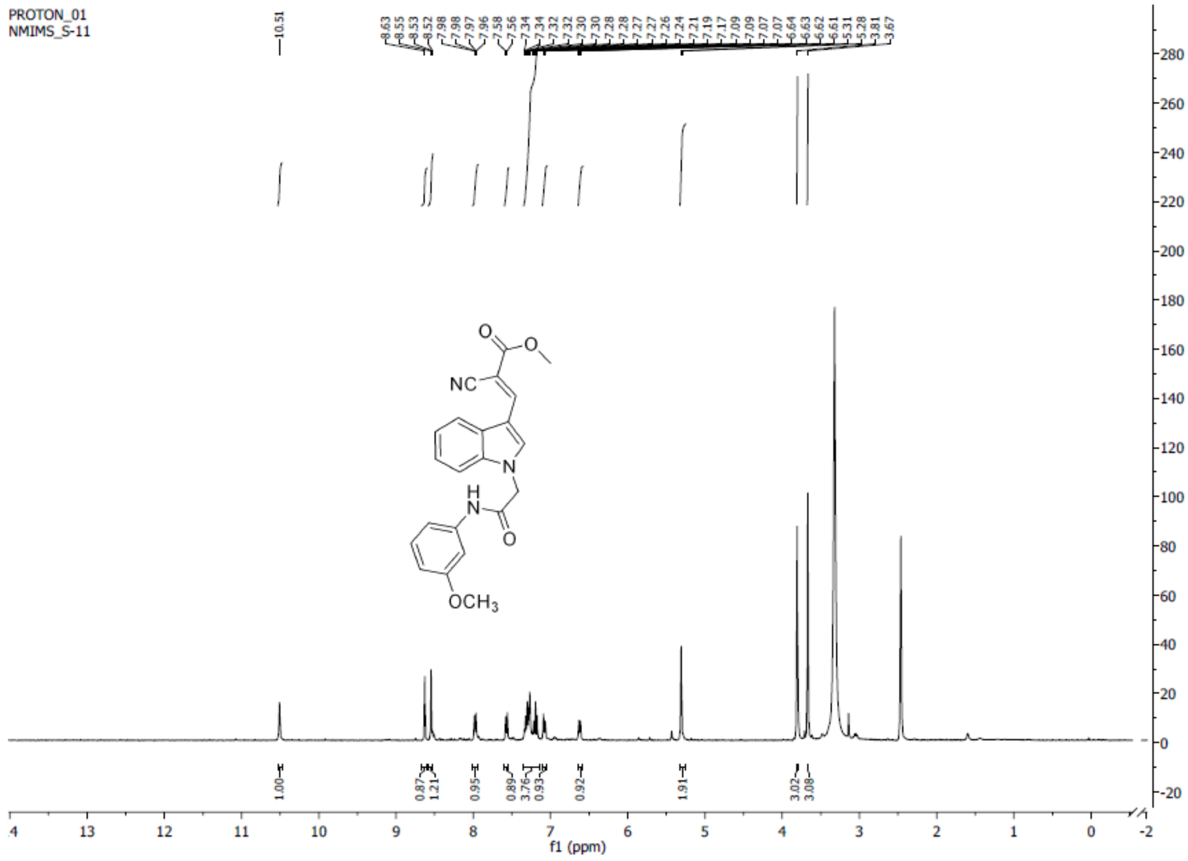

Figure S26 ^{1}H NMR spectrum of compound **23**.

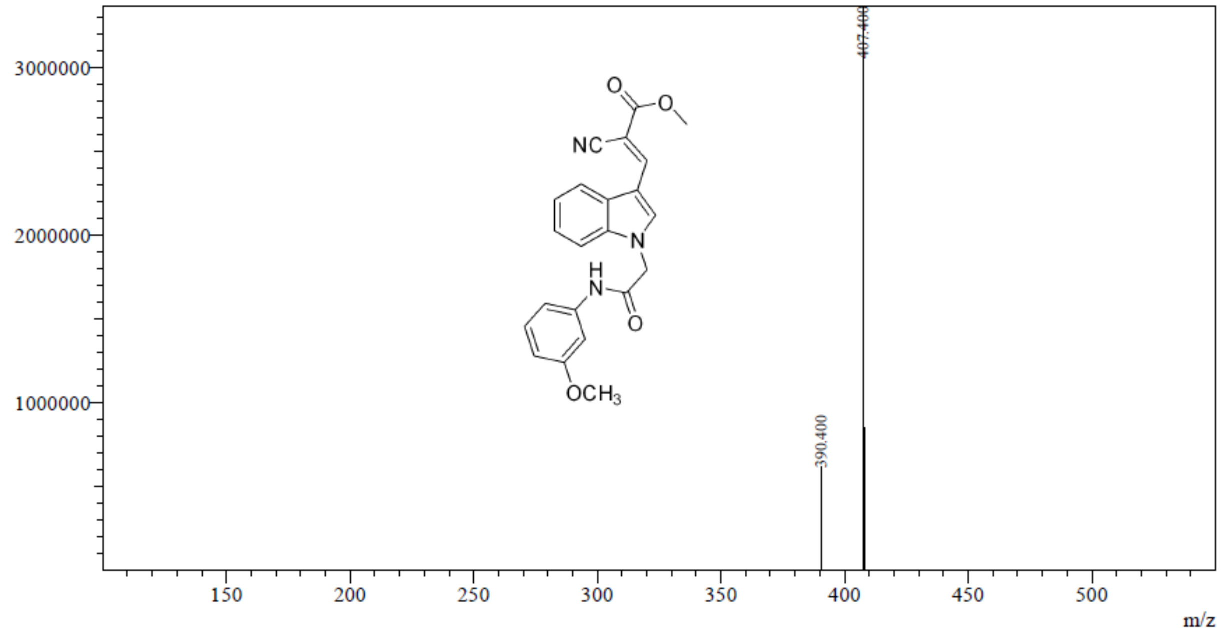

Figure S27 MS spectrum of compound **23**.

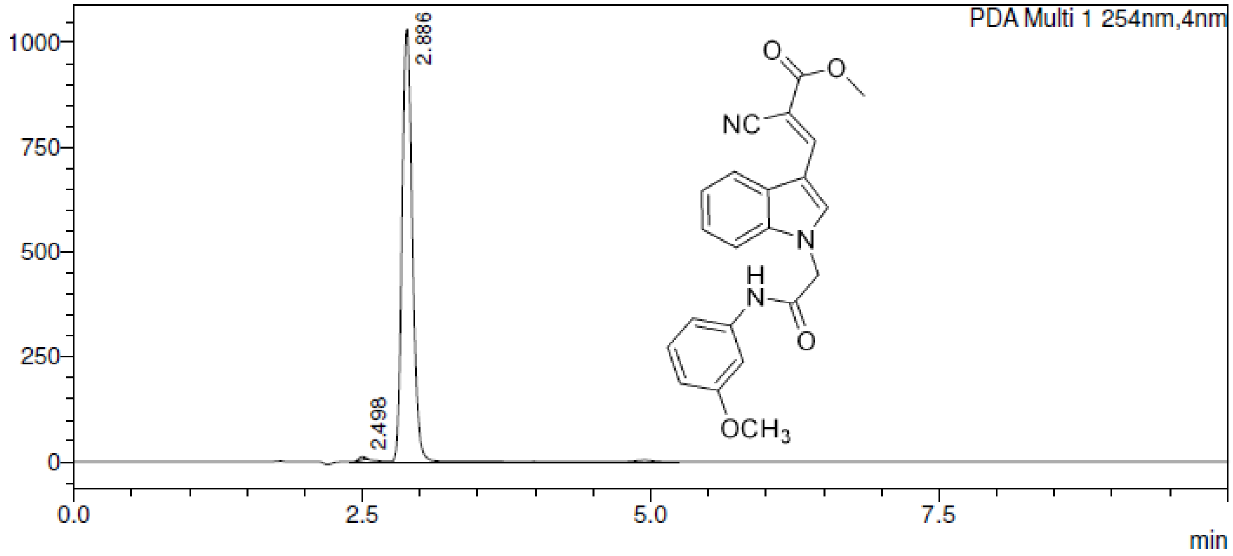

PDA Ch1 254nm

Peak#	Ret. Time	Area	Height	Area%	Height%
1	2.498	22015	6119	0.334	0.590
2	2.886	6574993	1031296	99.666	99.410
Total		6597008	1037415	100.000	100.000

Figure S28 HPLC chromatogram of compound **23**.

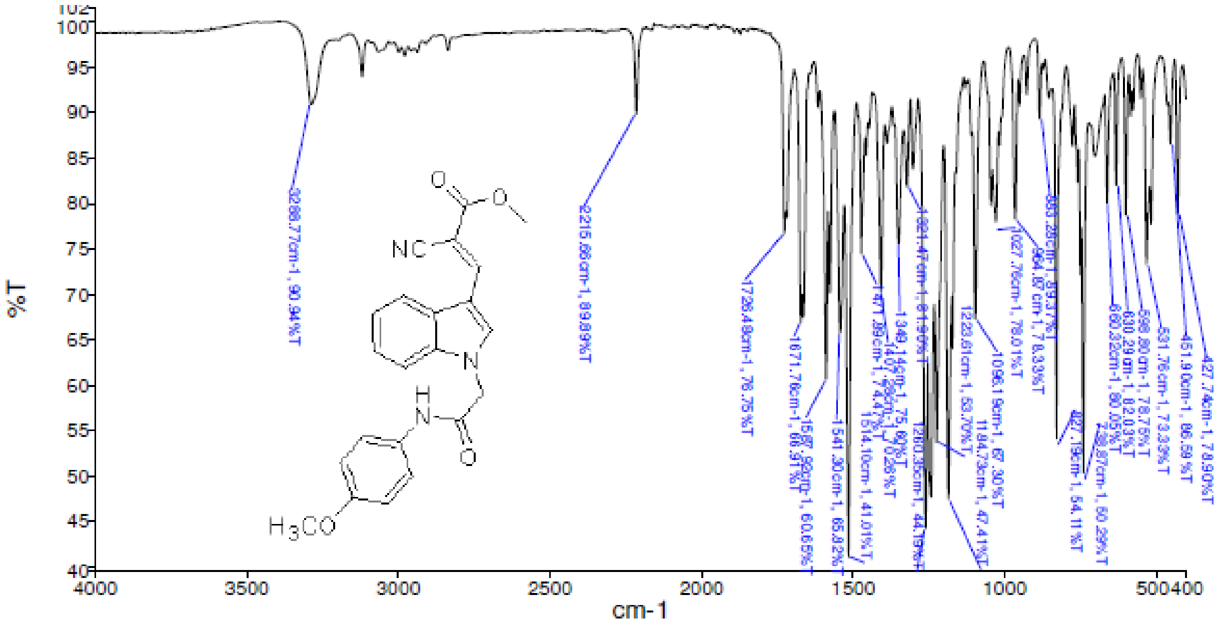

Figure S29 FTIR spectrum of compound **24**.

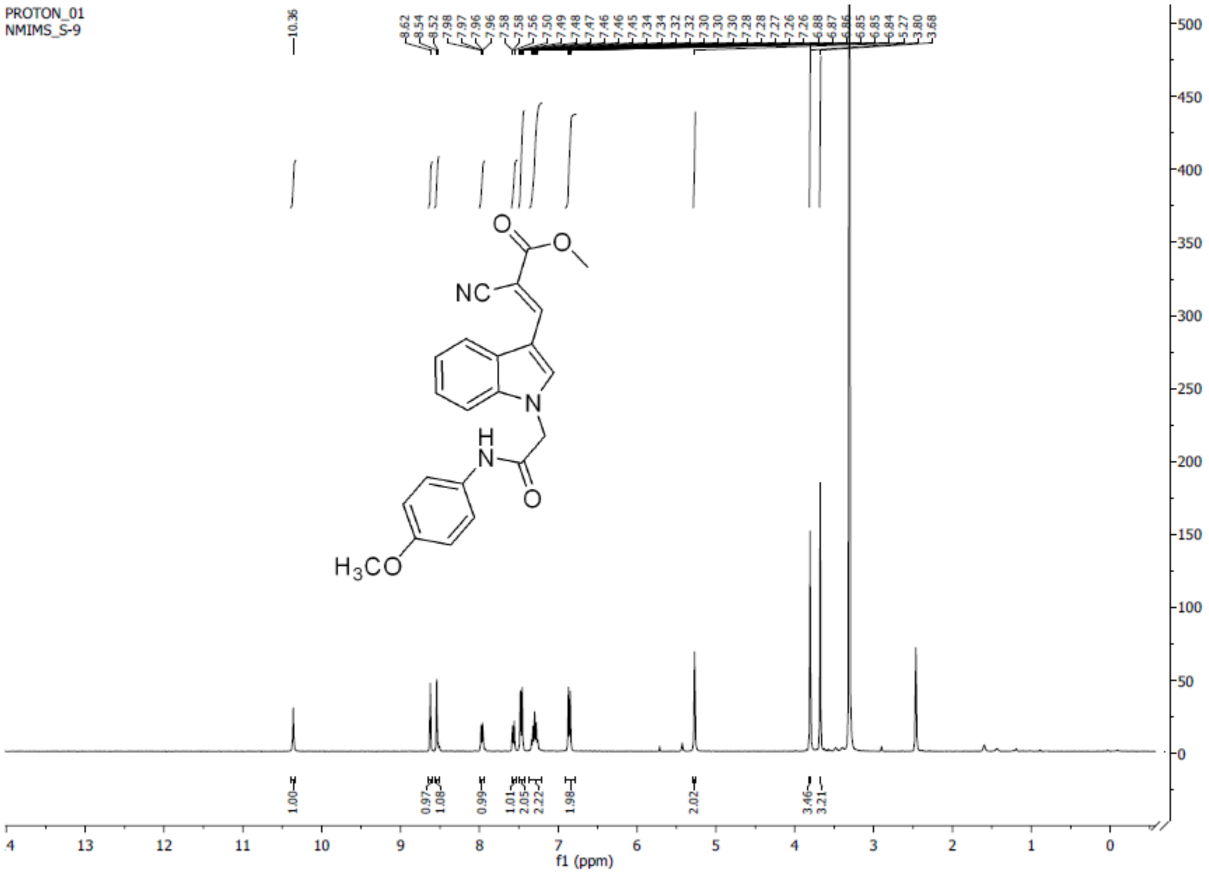

Figure S30 ^{1}H NMR spectrum of compound **24**.

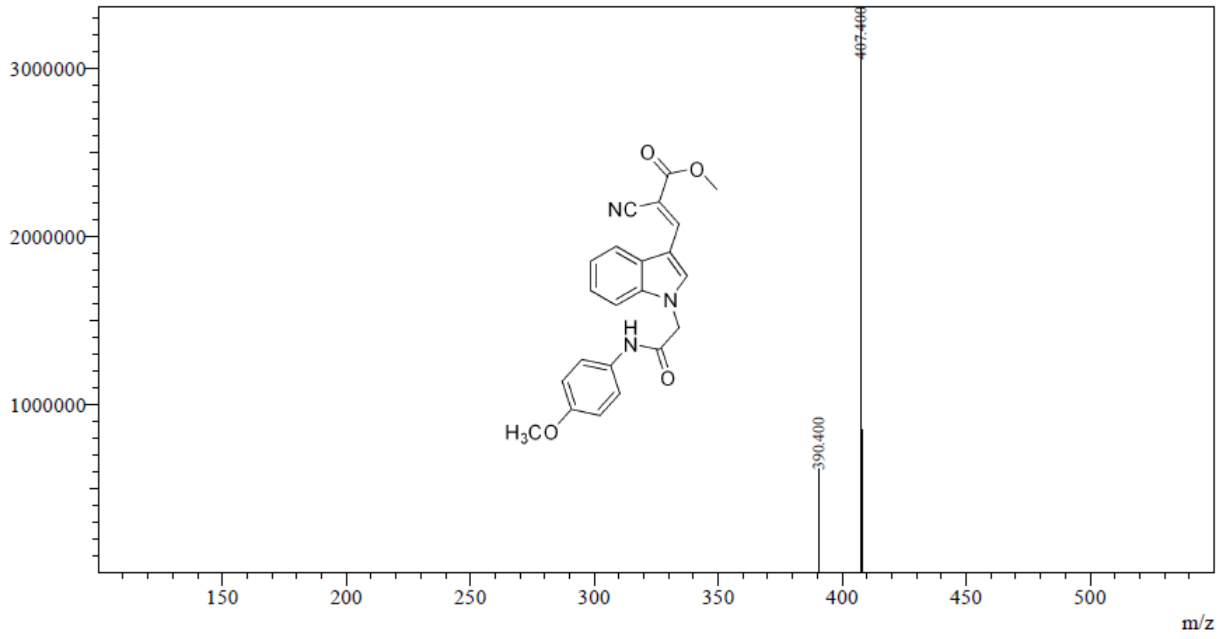

Figure S31 MS spectrum of compound **24**.

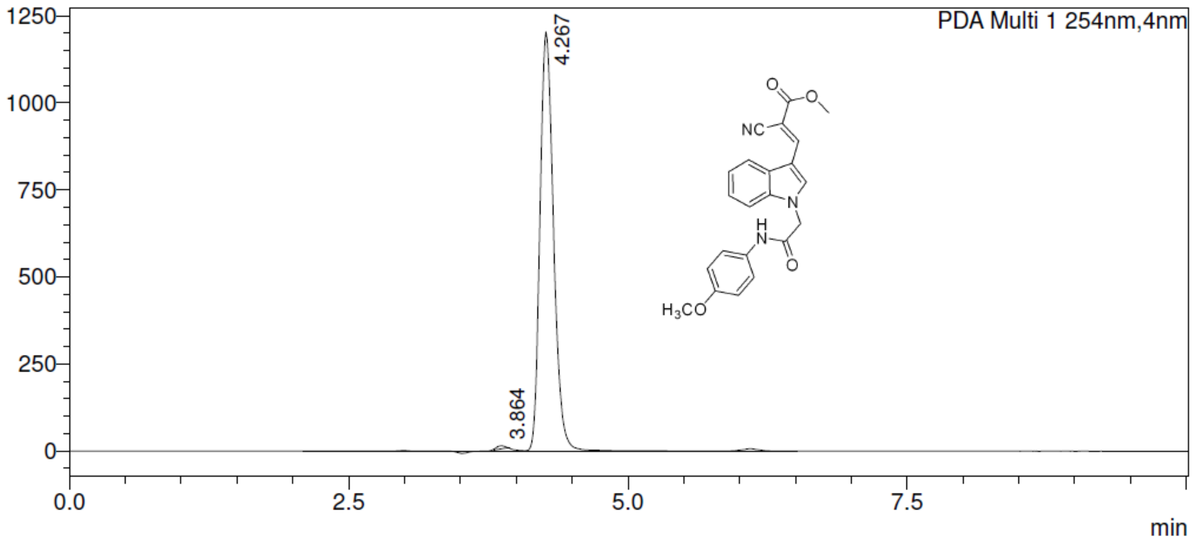

PDA Ch1 254nm

Peak#	Ret. Time	Area	Height	Area%	Height%
1	3.864	42895	8381	0.413	0.691
2	4.267	10344127	1203780	99.587	99.309
Total		10387022	1212162	100.000	100.000

Figure S32 HPLC chromatogram of compound **24**.

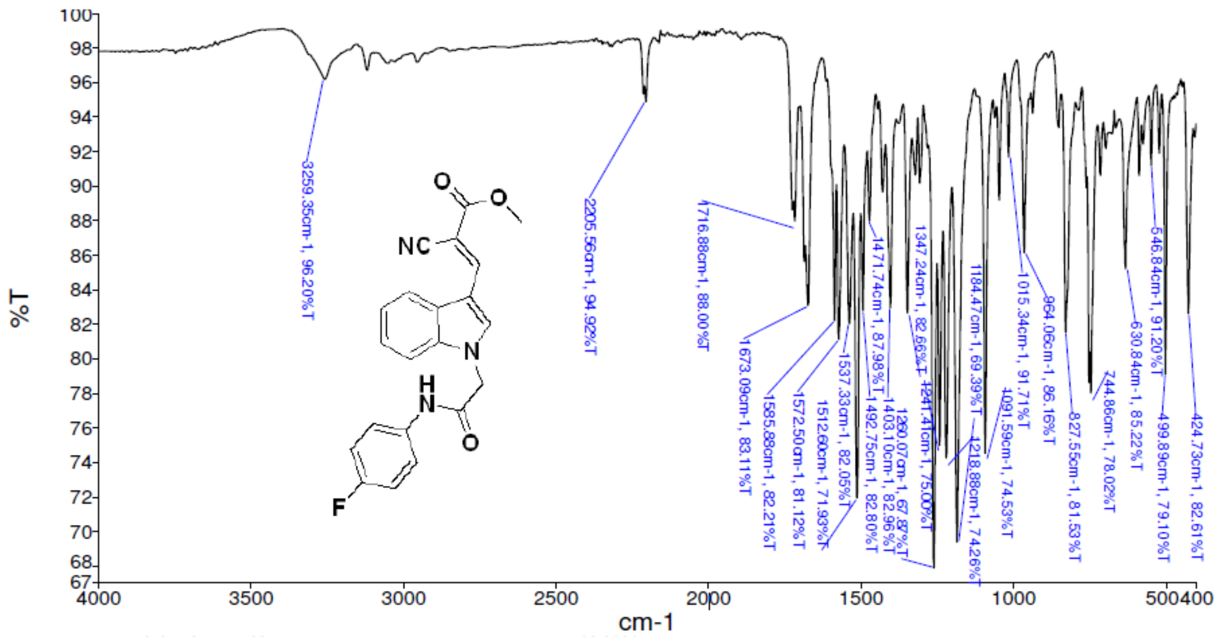

Figure S33 FTIR spectrum of compound **25**.

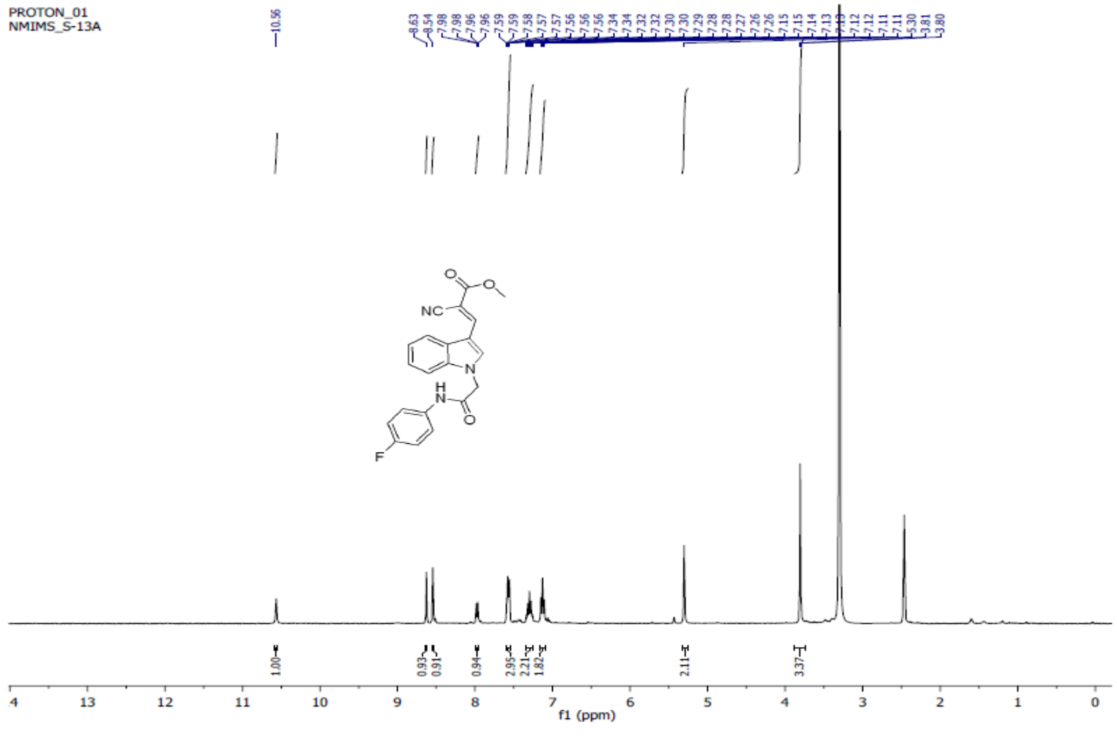

Figure S34 ^{1}H NMR spectrum of compound **25**.

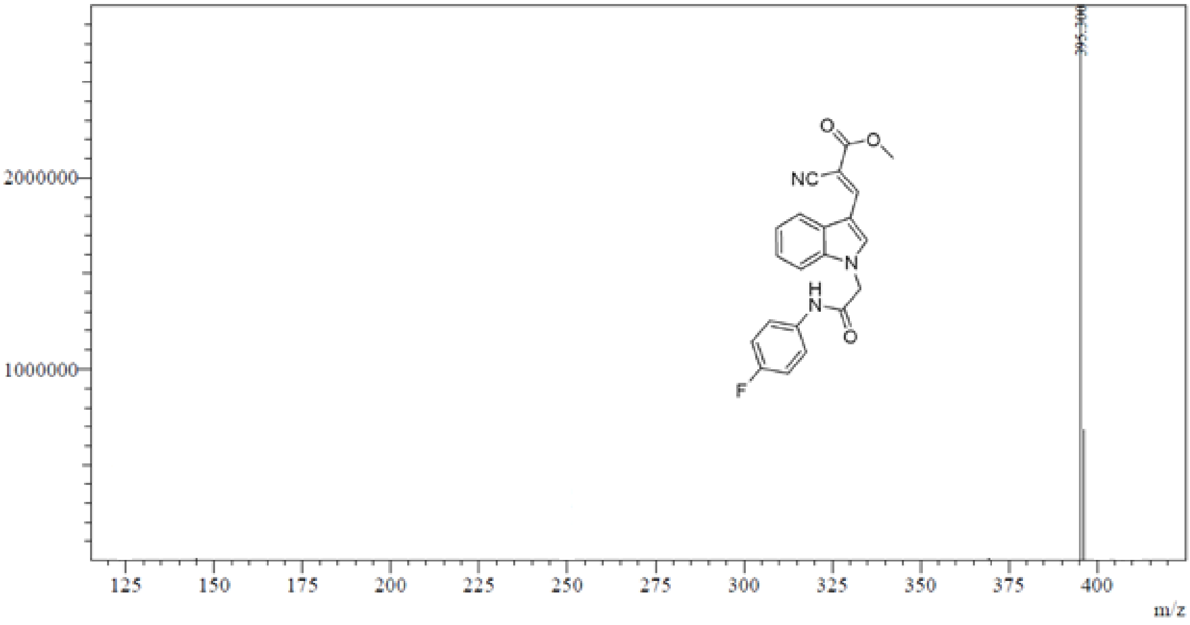

Figure S35 MS spectrum of Compound **25**.

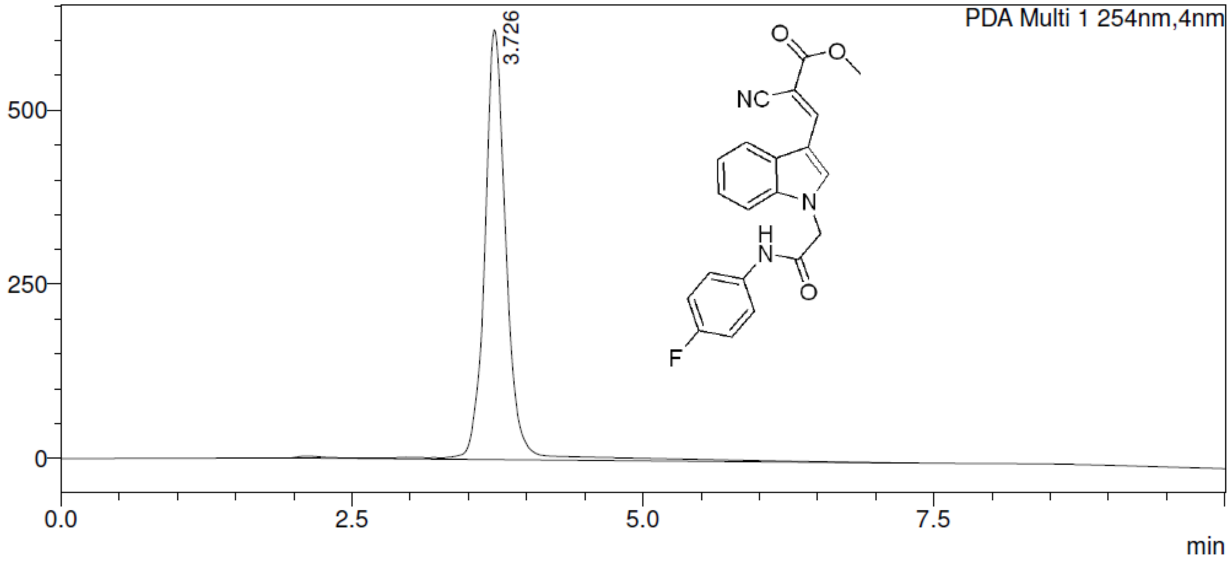

PDA Ch1 254nm

Peak#	Ret. Time	Area	Height	Area%	Height%
1	3.726	8276142	616974	100.000	100.000
Total		8276142	616974	100.000	100.000

Figure S36 HPLC chromatogram of compound **25**.

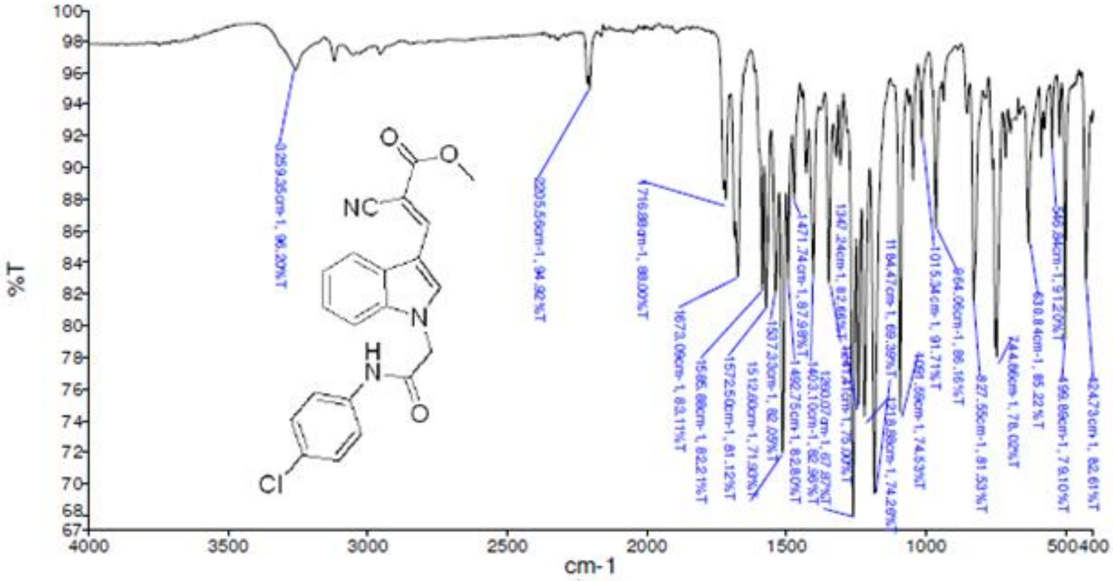

Figure S37 FTIR spectrum of compound **26**.

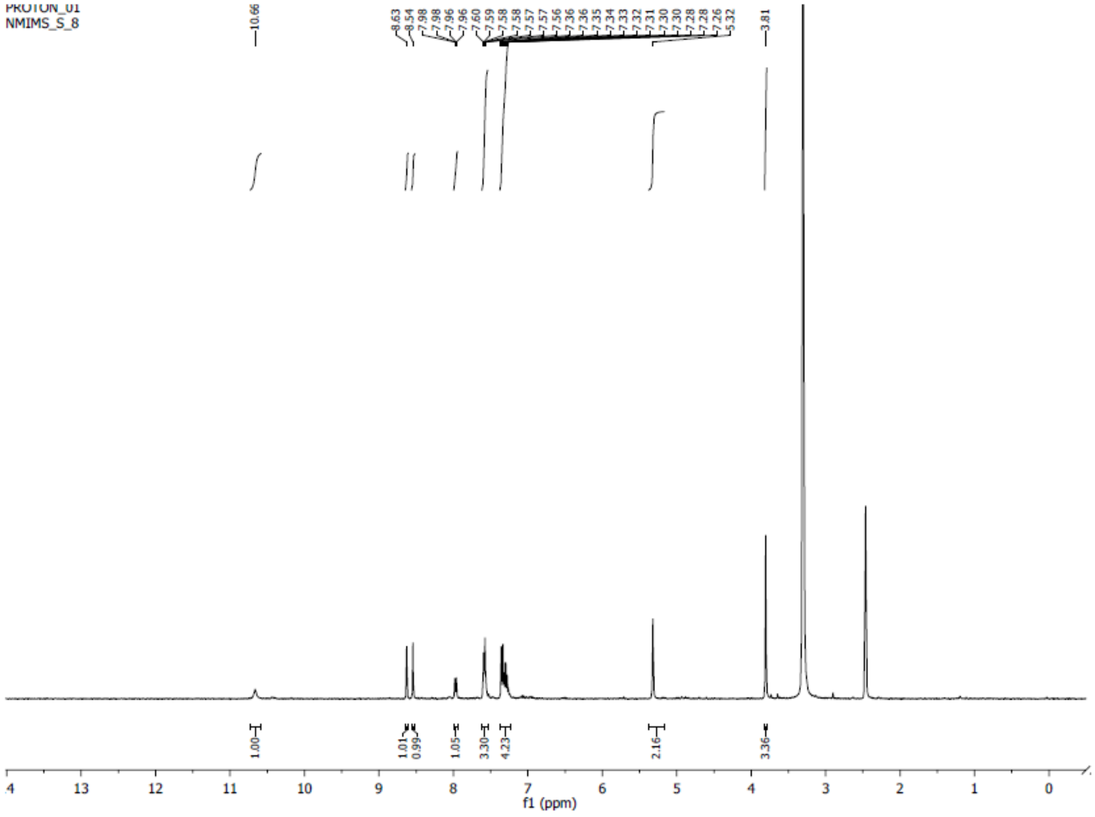

Figure S38 ^{1}H NMR spectrum of compound **26**.

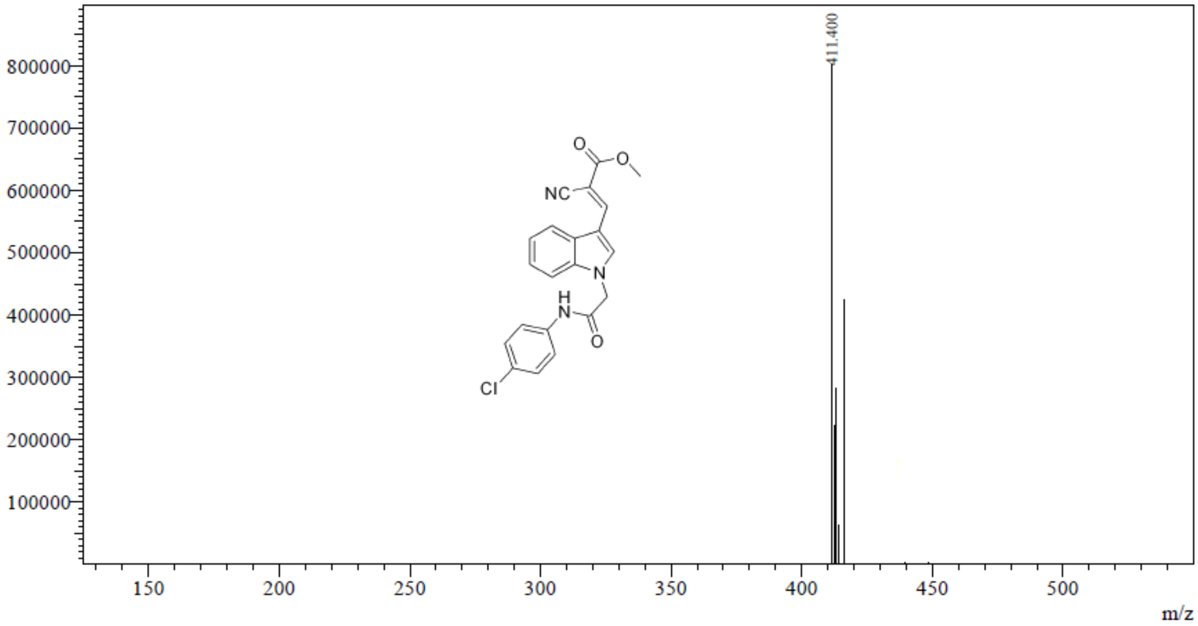

Figure S39 MS spectrum of compound **26**.

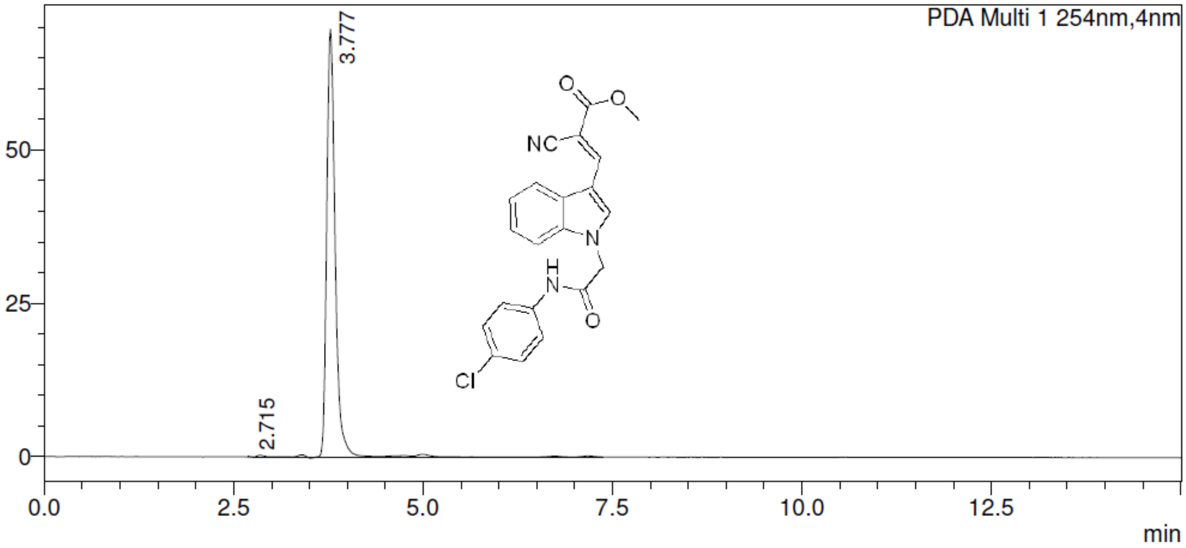

PDA Ch1 254nm

Peak#	Ret. Time	Area	Height	Area%	Height%
1	2.715	-2	5	-0.000	0.007
2	3.777	553281	69821	100.000	99.993
Total		553279	69826	100.000	100.000

Figure S40 HPLC Chromatogram of compound **26**.

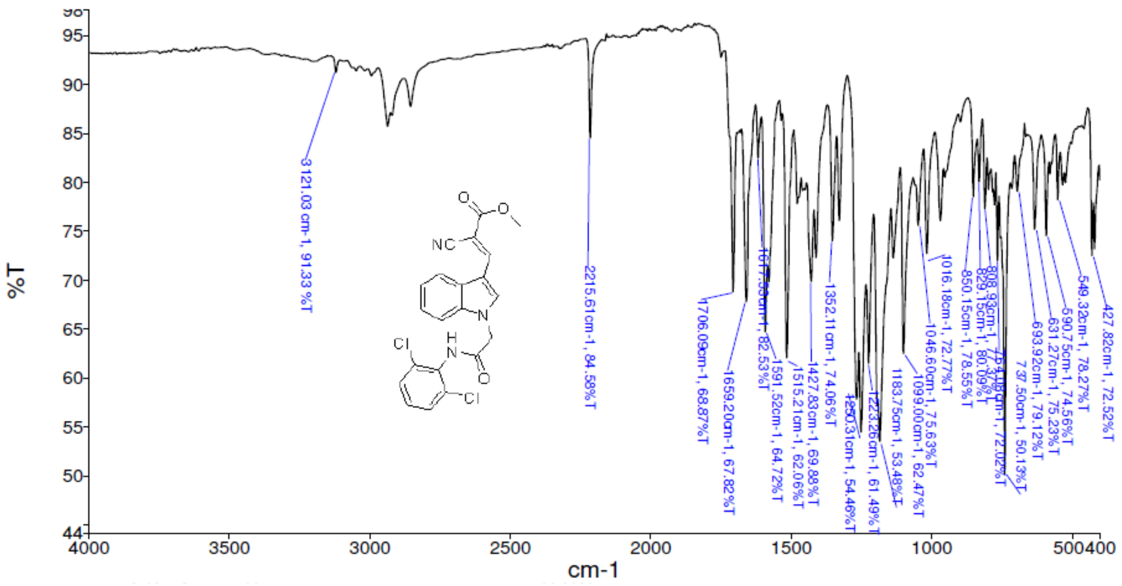

Figure S41 FTIR spectrum of compound **27**.

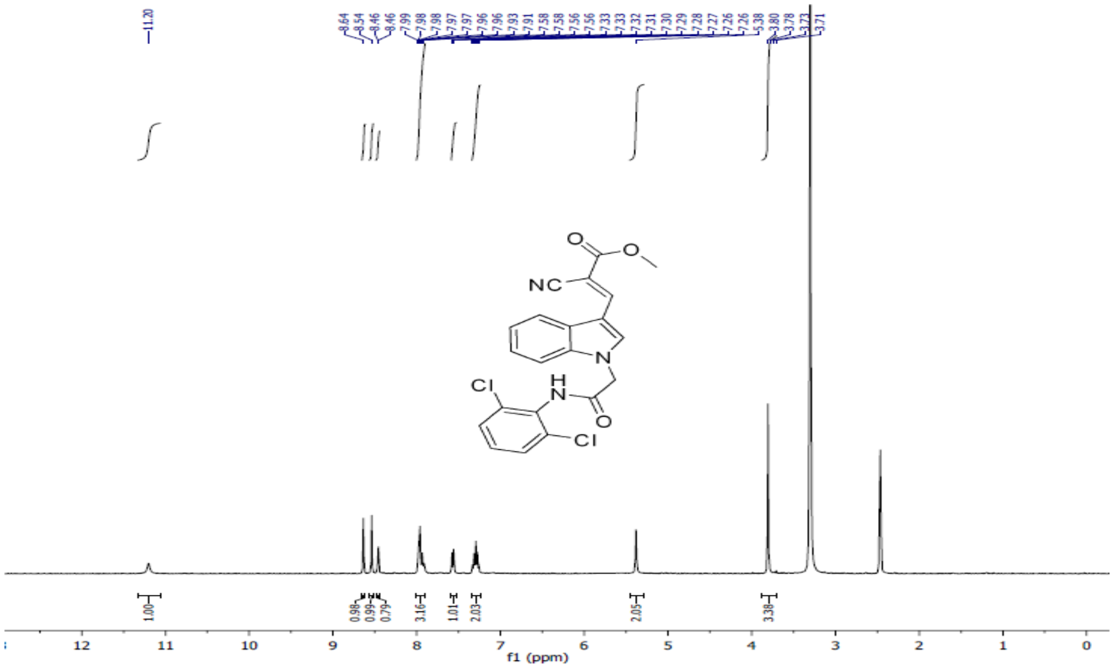

Figure S42 ^{1}H NMR spectrum of compound **27**.

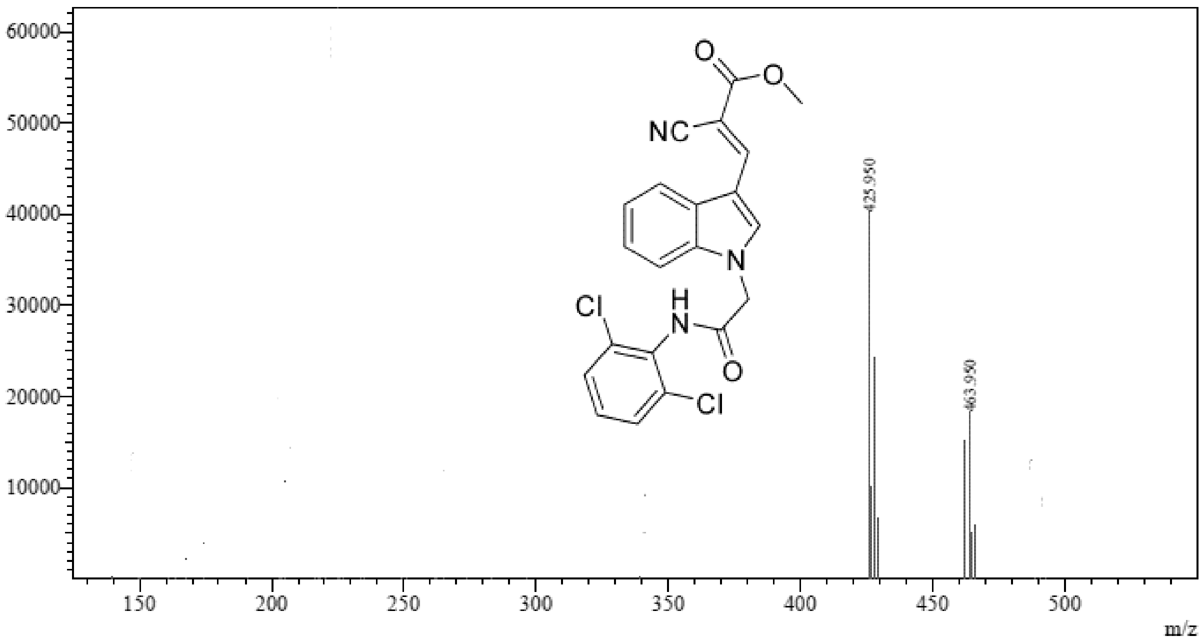

Figure S43 MS spectrum of compound **27**.

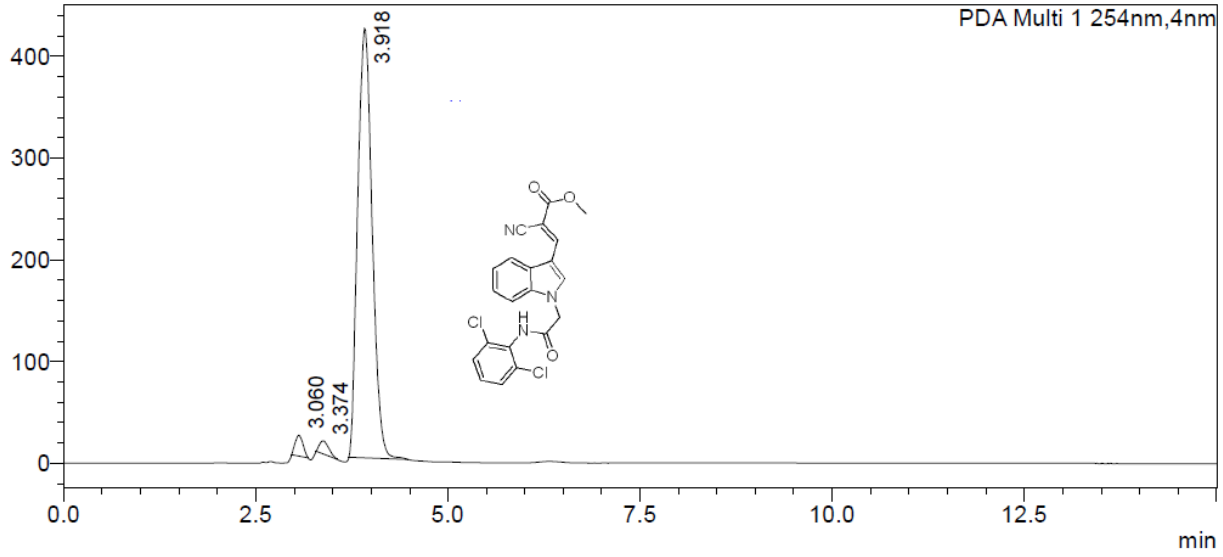

PDA Ch1 254nm

Peak#	Ret. Time	Area	Height	Area%	Height%
1	3.060	144228	20370	2.494	4.477
2	3.374	106315	12693	1.839	2.790
3	3.918	5532079	421896	95.667	92.733
Total		5782622	454959	100.000	100.000

Figure S44 HPLC chromatogram of compound **27**.

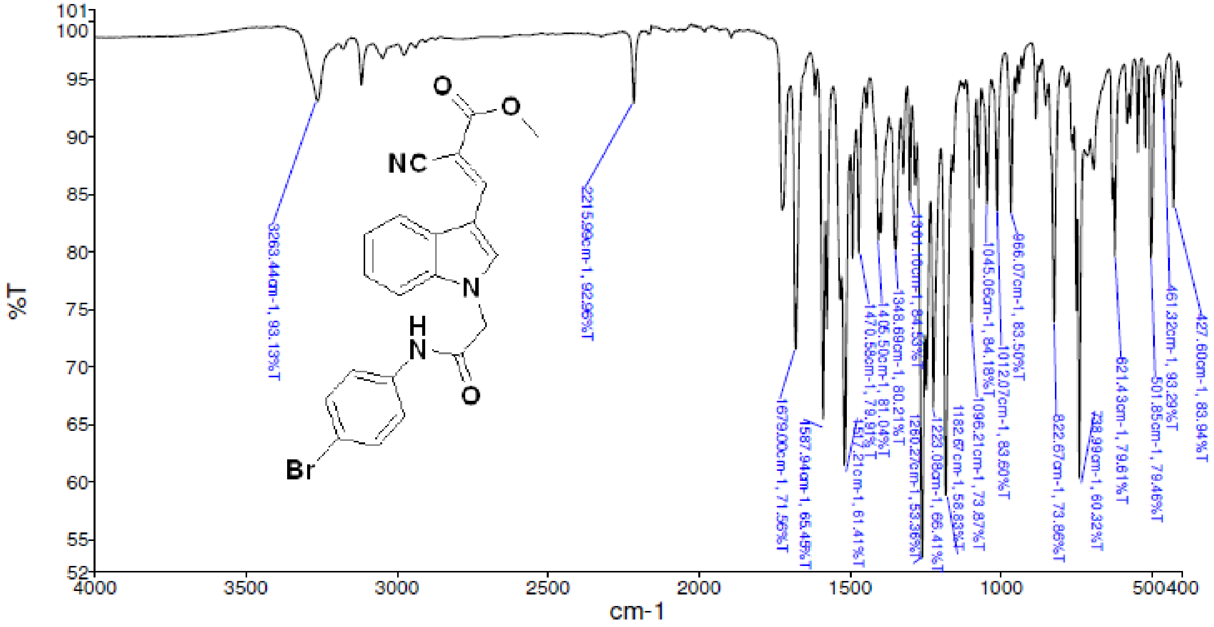

Figure S45 FTIR spectrum of compound **28**.

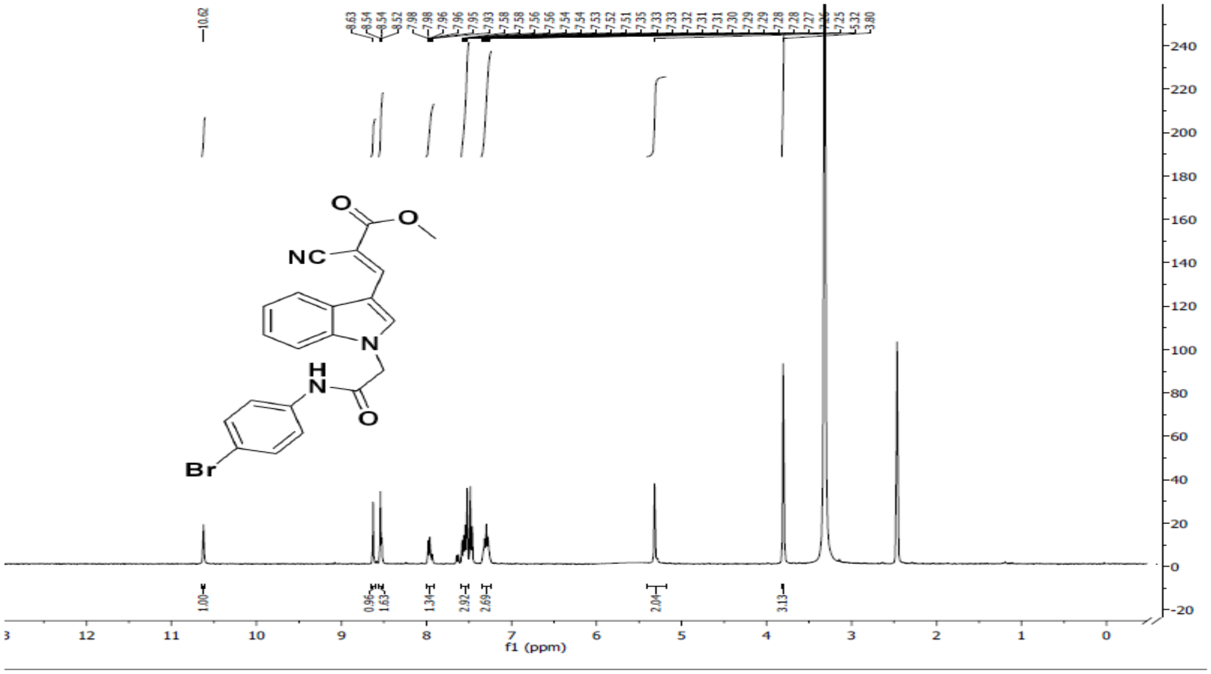

Figure S46 ^{1}H NMR spectrum of compound **28**.

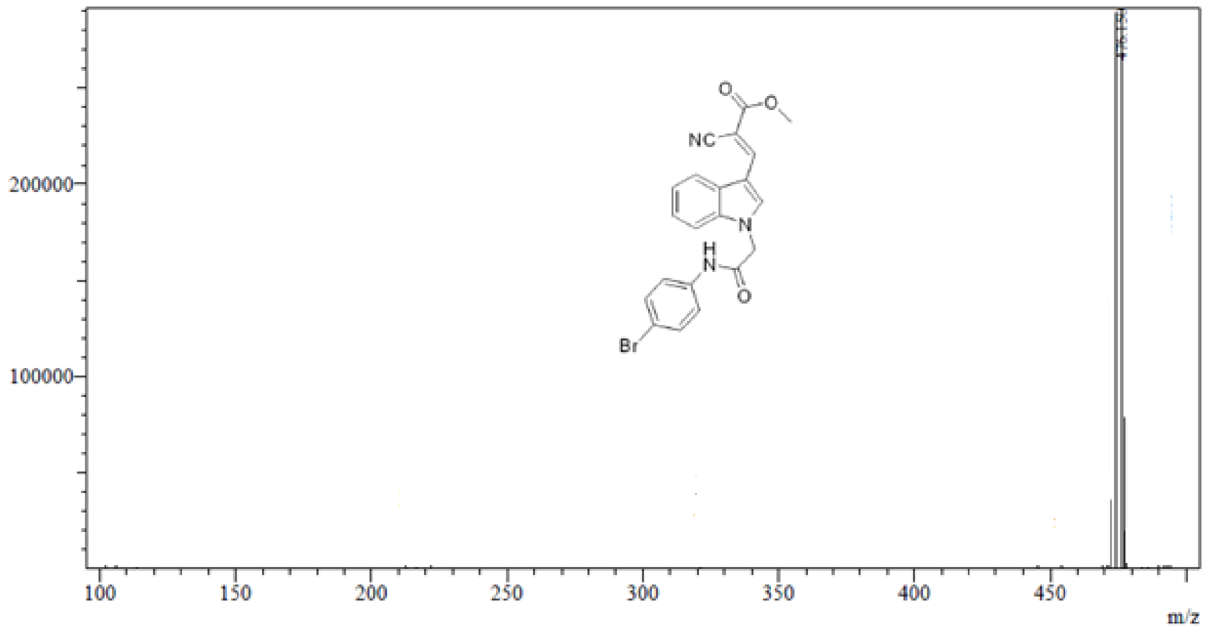

Figure S47 MS spectrum of compound **28**.

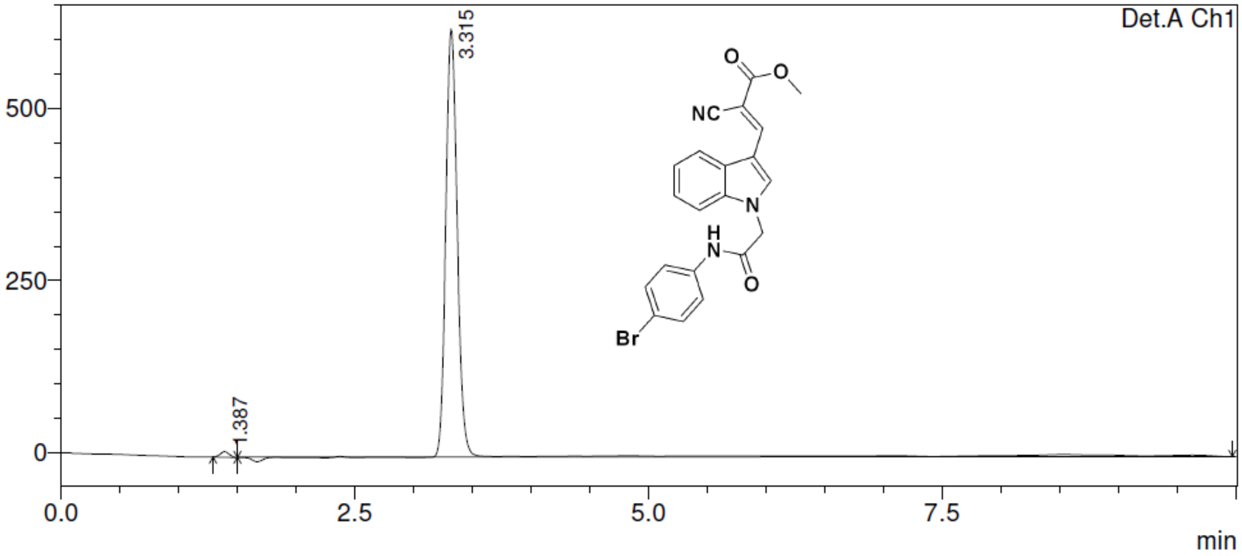

PeakTable

Detector A Ch1 254nm

Peak#	Ret. Time	Area	Height	Area %	Height %
1	1.387	43788	8288	0.921	1.315
2	3.315	4708904	621957	99.079	98.685
Total		4752692	630245	100.000	100.000

Figure S48 HPLC chromatogram of compound **28**.

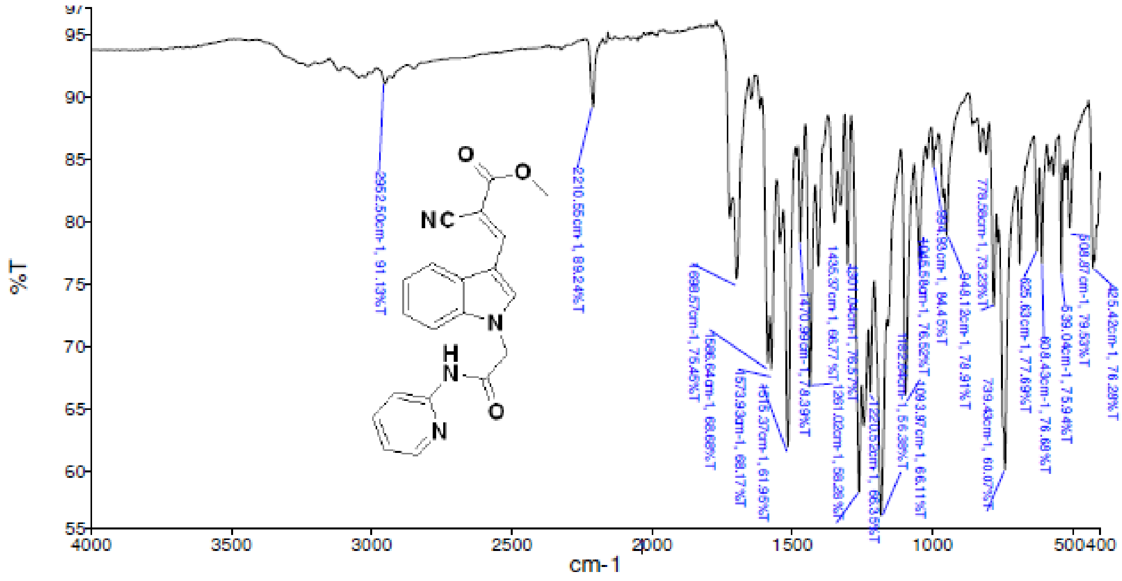

Figure S49 FTIR spectrum of compound **29**.

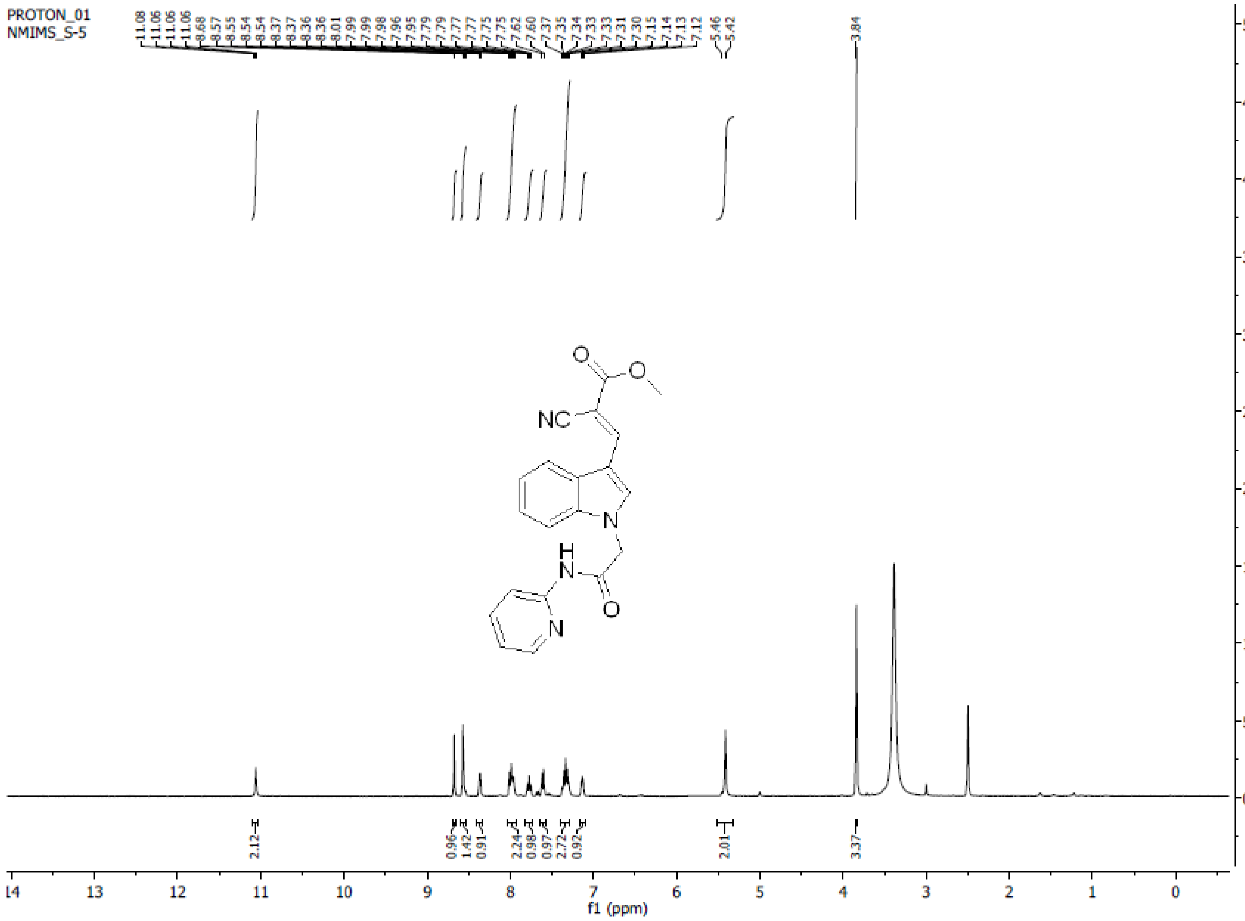

Figure S50 ^{1}HNMR spectrum of compound **29**.

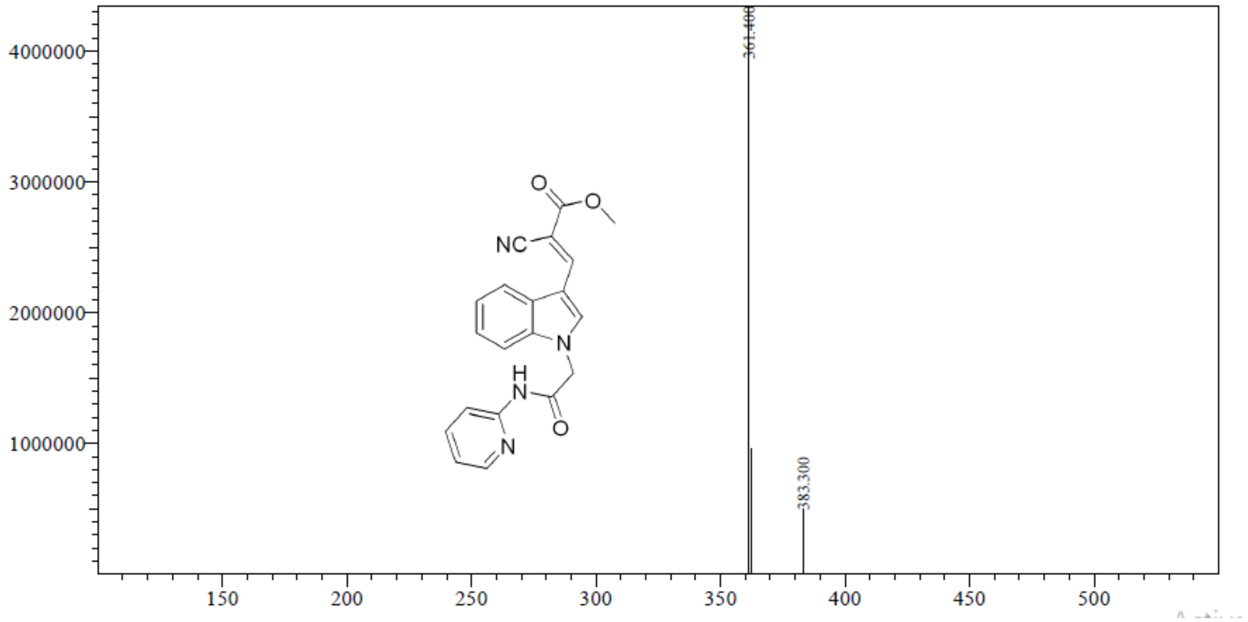

Figure S51 MS spectrum of compound **29**.

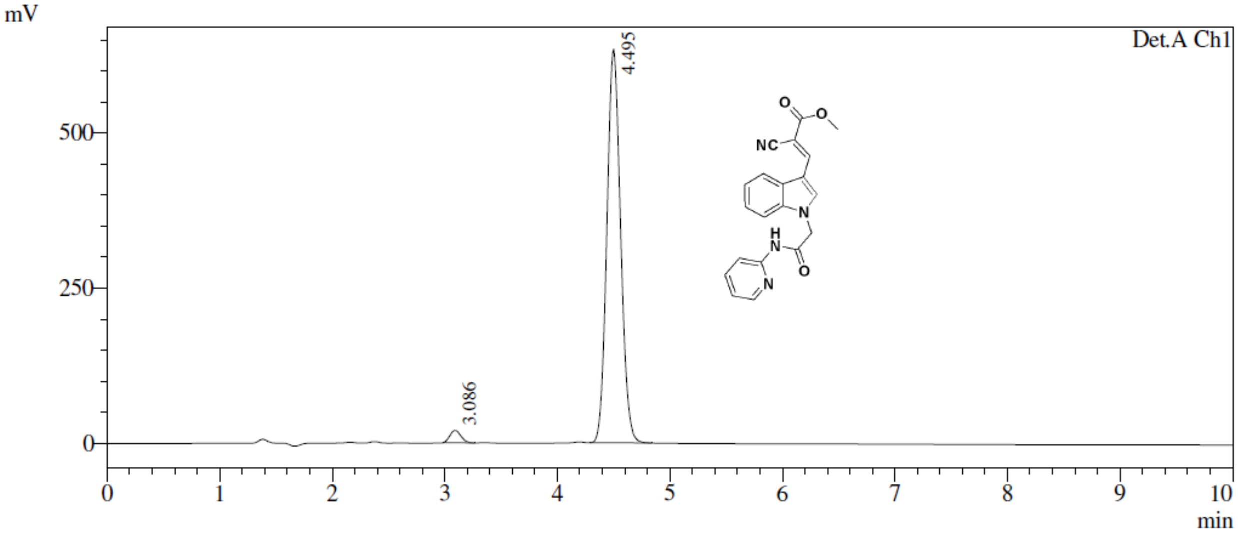

Peak#	Ret. Time	Area	Height	Area %	Height %
1	3.086	133049	19911	2.411	3.043
2	4.495	5385842	634471	97.589	96.957
Total		5518891	654382	100.000	100.000

Figure S52 HPLC chromatogram of compound **29**.

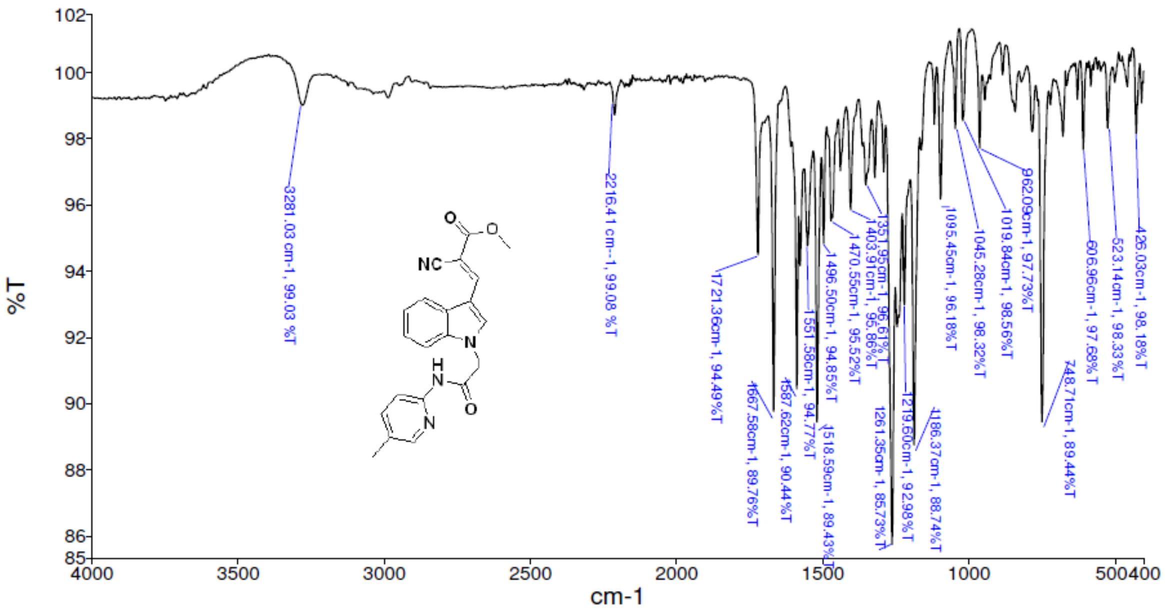

Figure S53 FTIR spectrum of compound **30**.

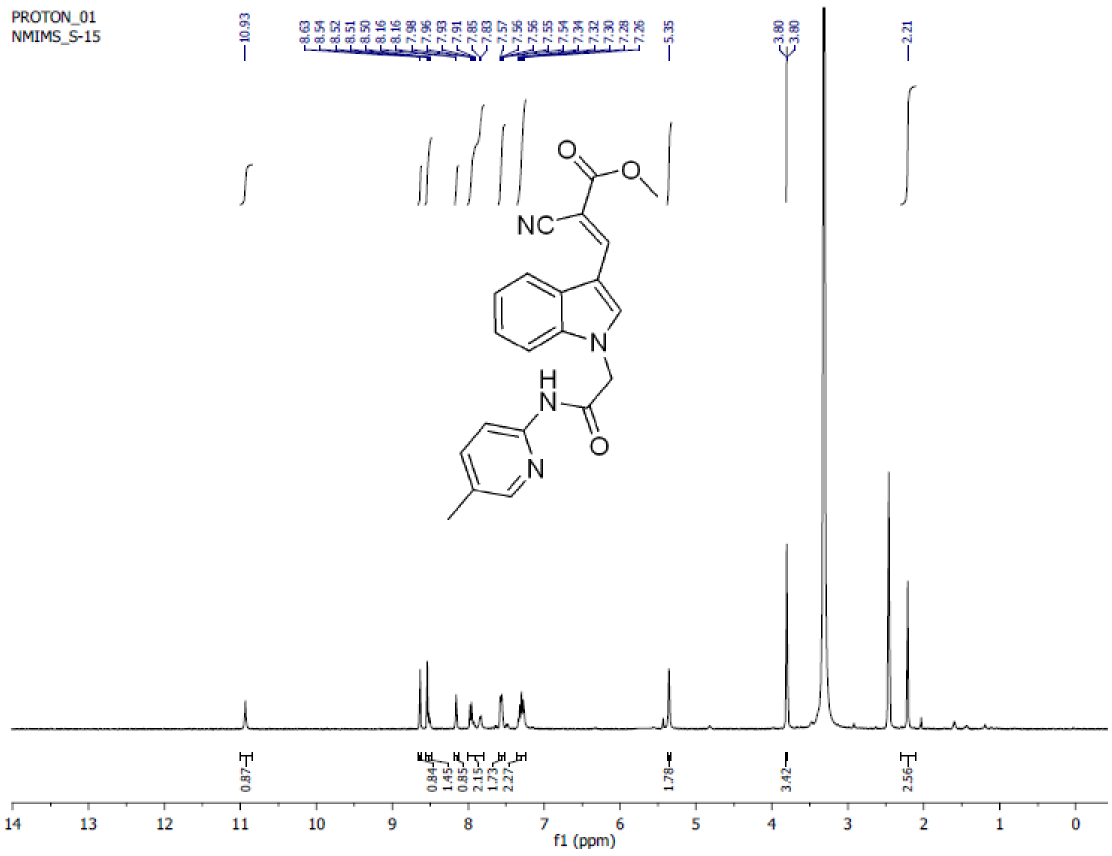

Figure S54 ^{1}H NMR spectrum of compound **30**.

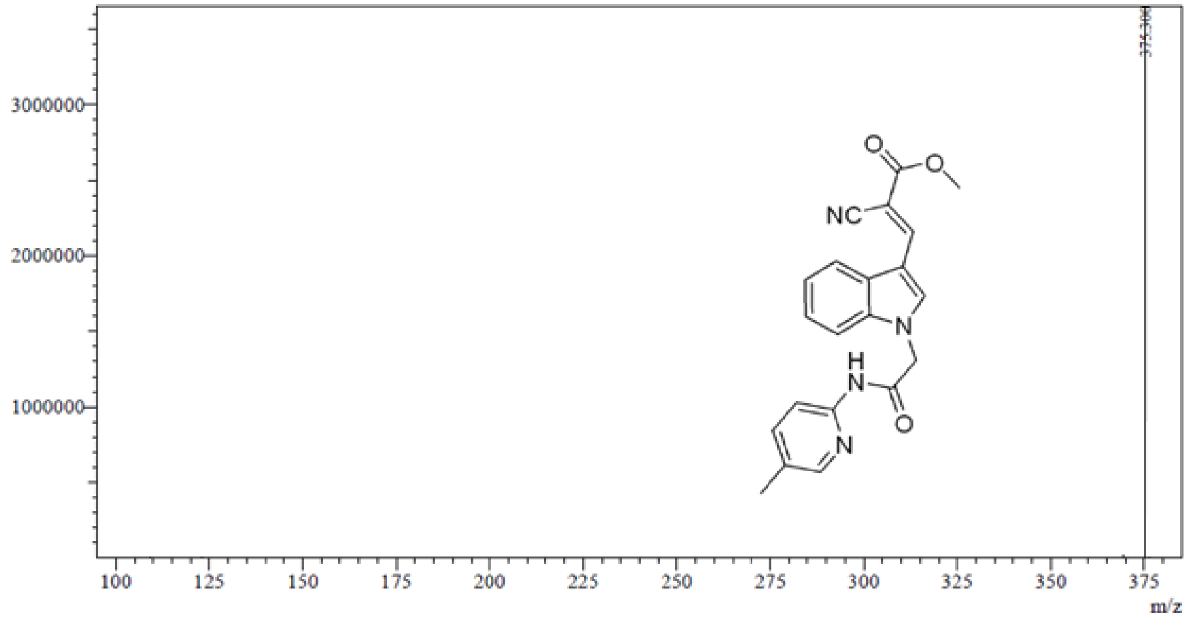

Figure S55 MS spectrum of compound **30**.

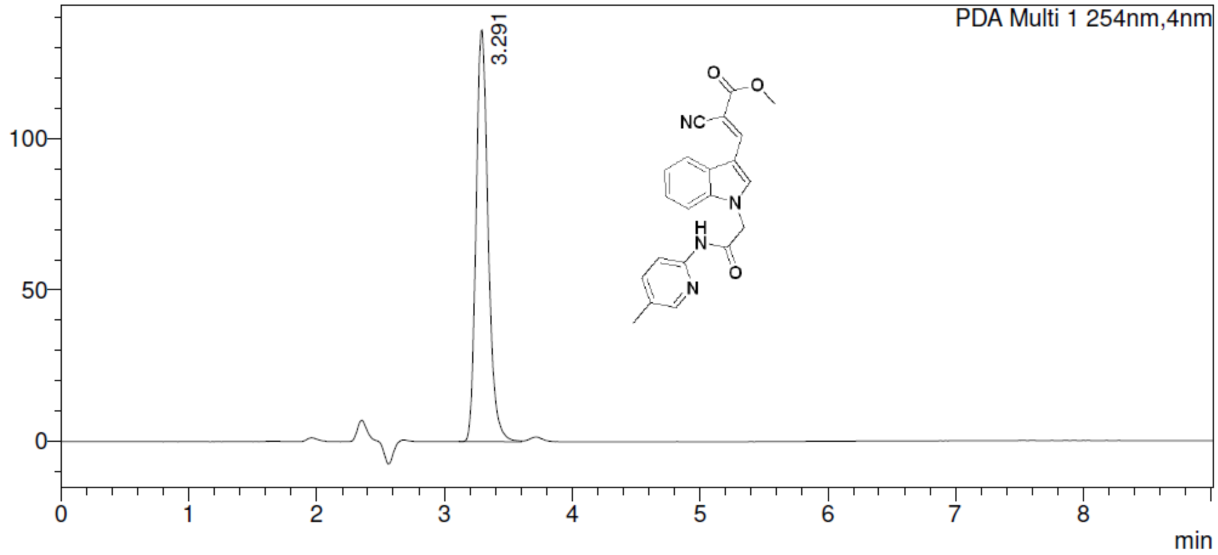

PDA Ch1 254nm

Peak#	Ret. Time	Area	Height	Area%	Height%
1	3.291	897779	135996	100.000	100.000
Total		897779	135996	100.000	100.000

Figure S56 HPLC chromatogram of compound **30**.

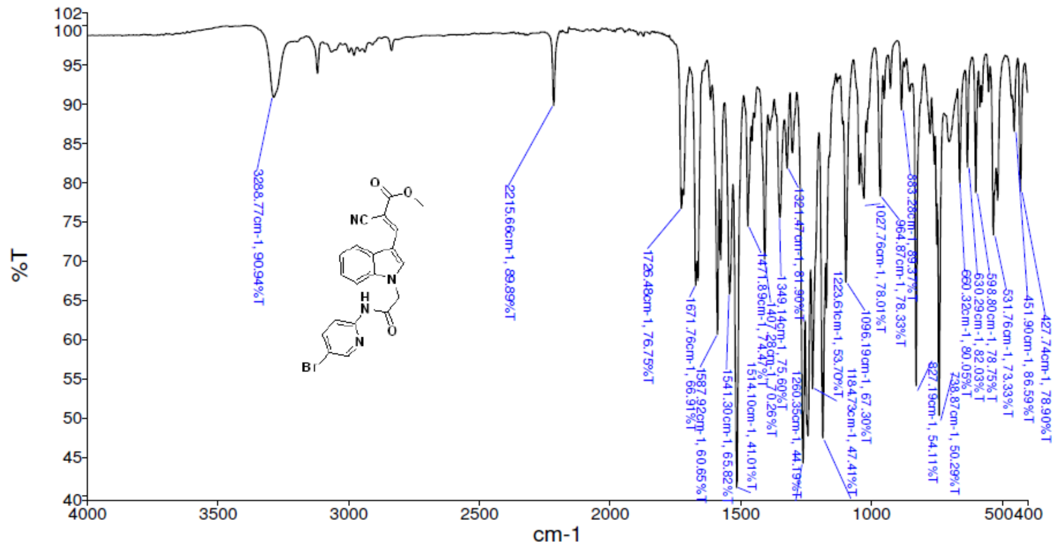

Figure S57 FTIR spectrum of compound **31**.

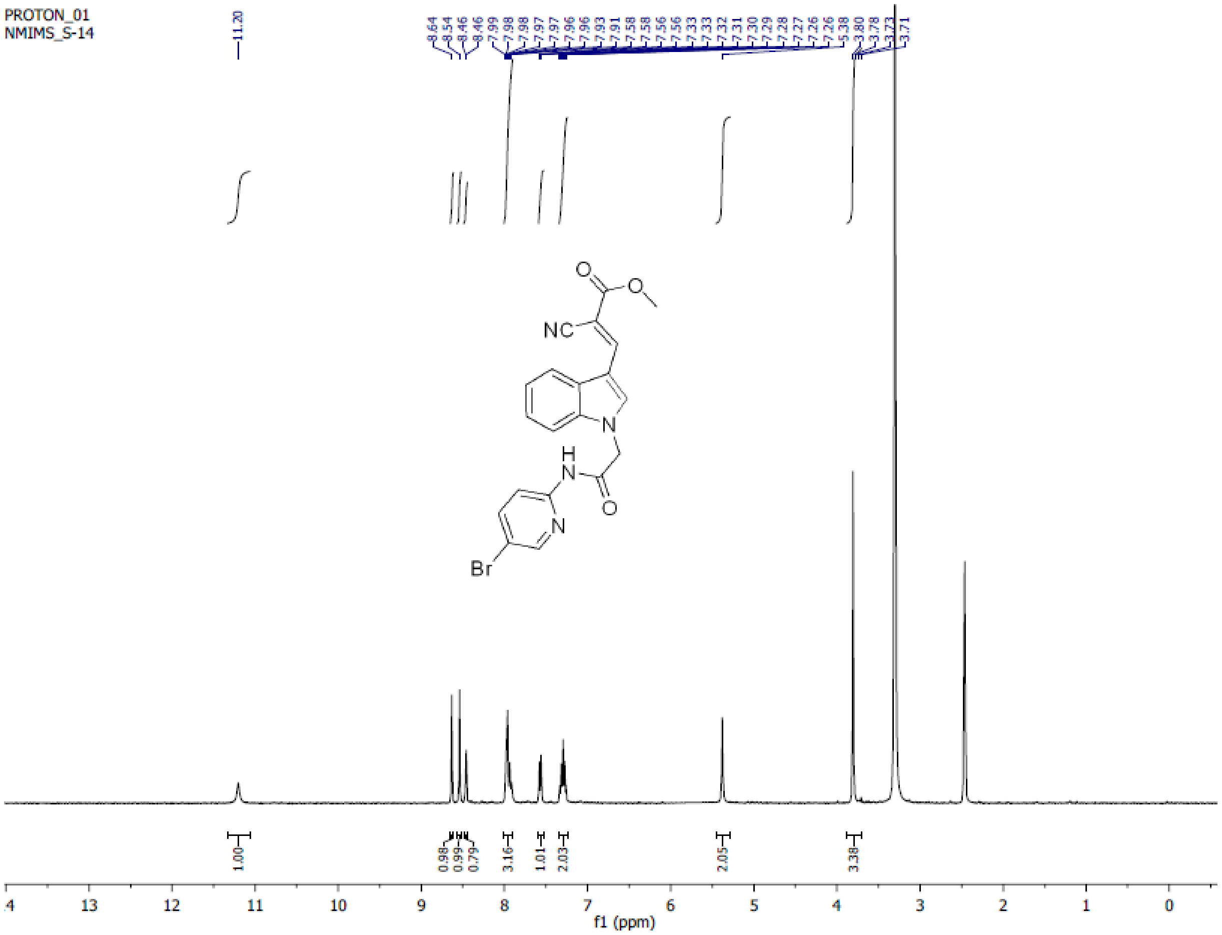

Figure S58 ^{1}H NMR spectrum of compound **31**

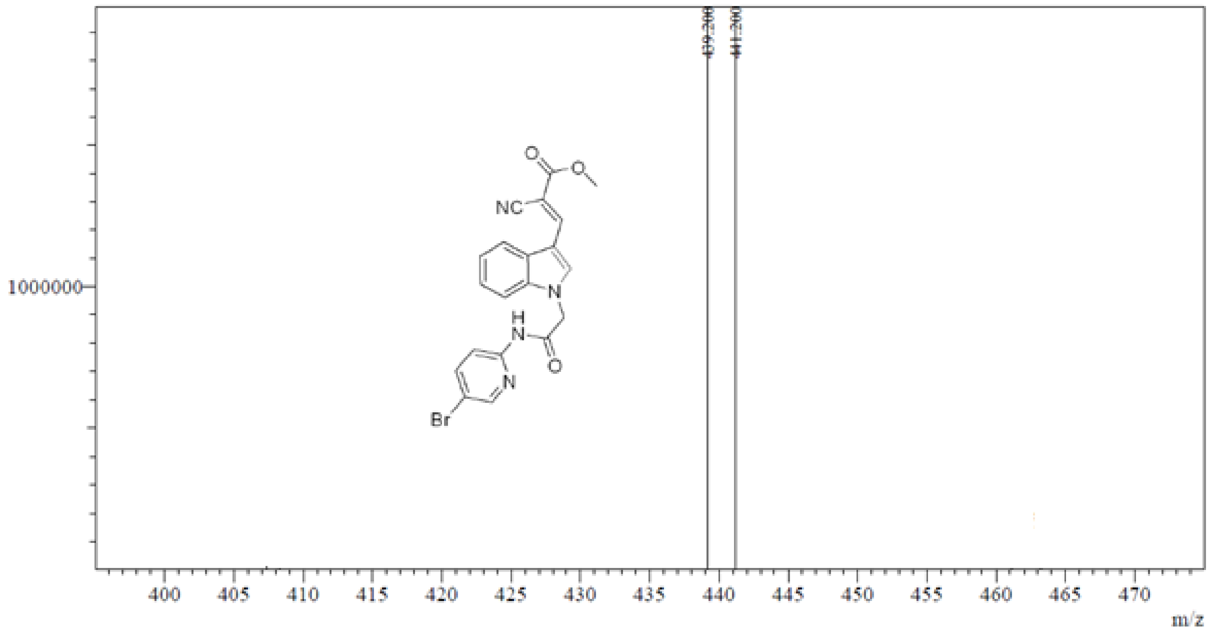

Figure S59 MS spectrum of compound **31**.

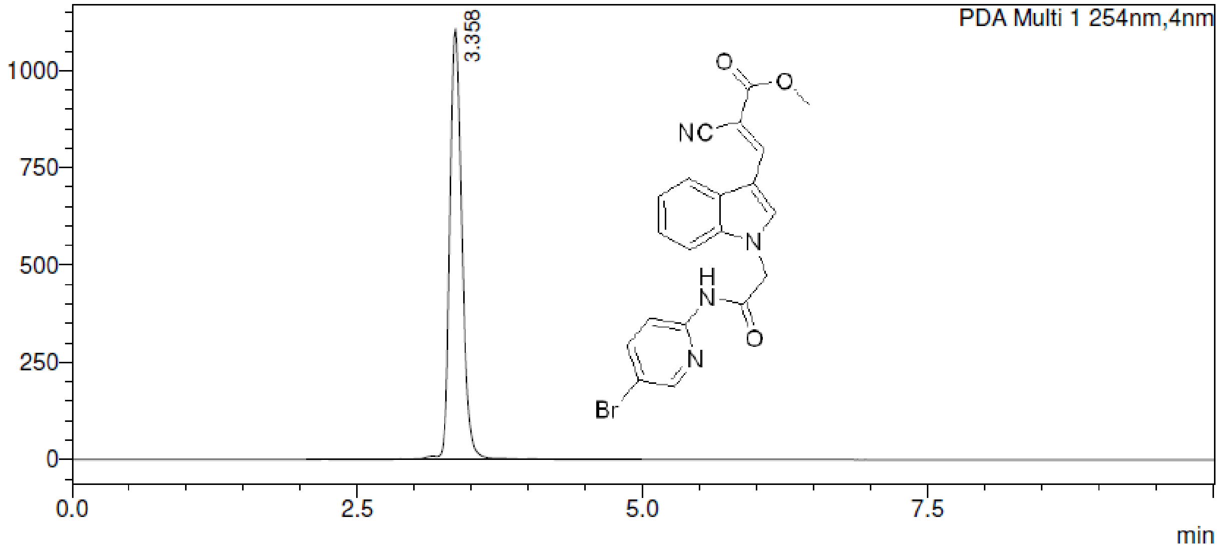

PDA Ch1 254nm

Peak#	Ret. Time	Area	Height	Area%	Height%
1	3.358	8221686	1107482	100.000	100.000
Total		8221686	1107482	100.000	100.000

Figure S60 HPLC chromatogram of compound **31**.

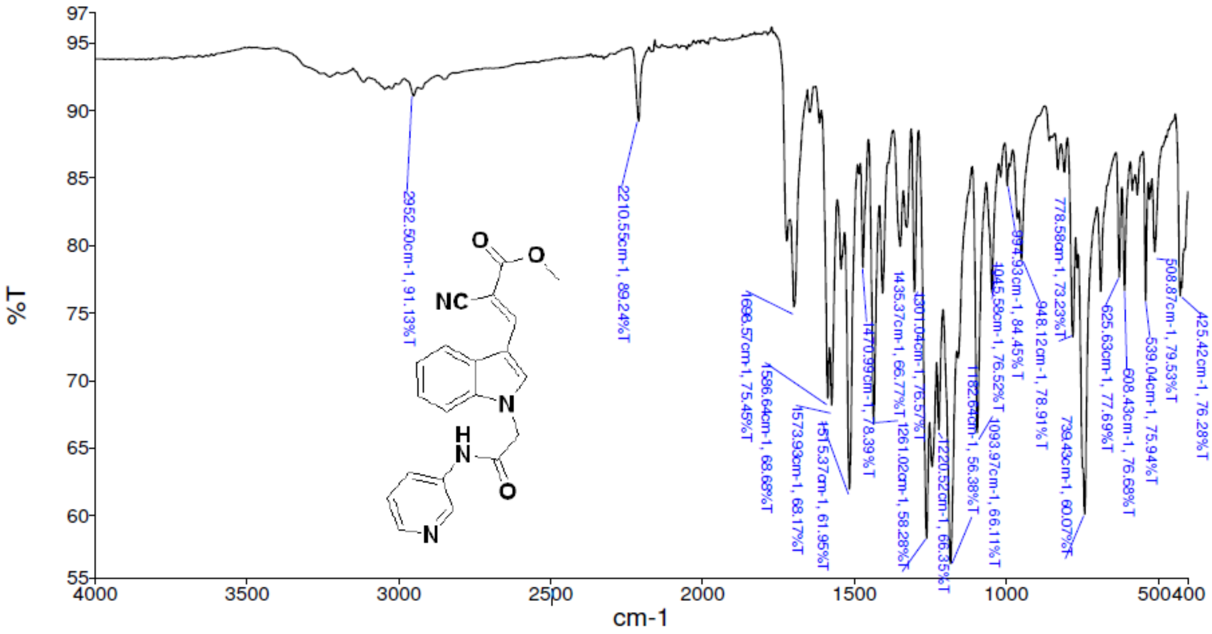

Figure S61 FTIR spectrum of compound **32**.

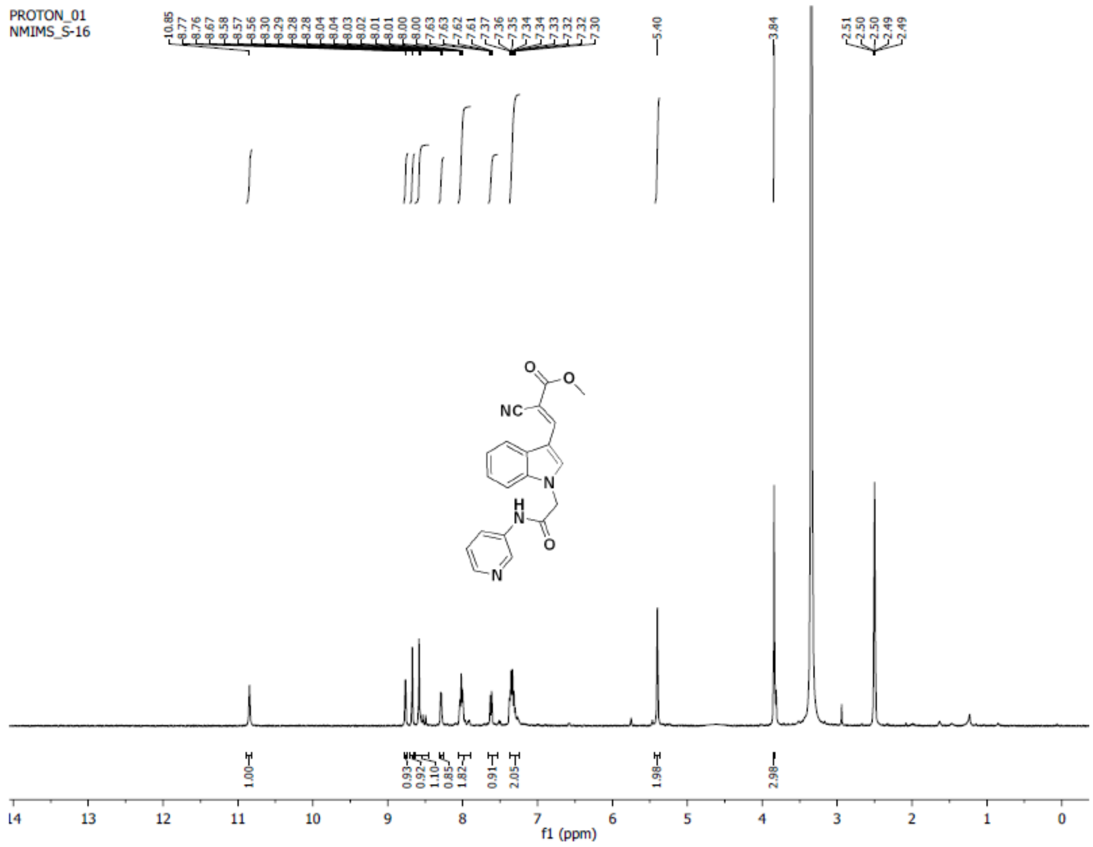

Figure S62 ^{1}H NMR spectrum of compound **32**.

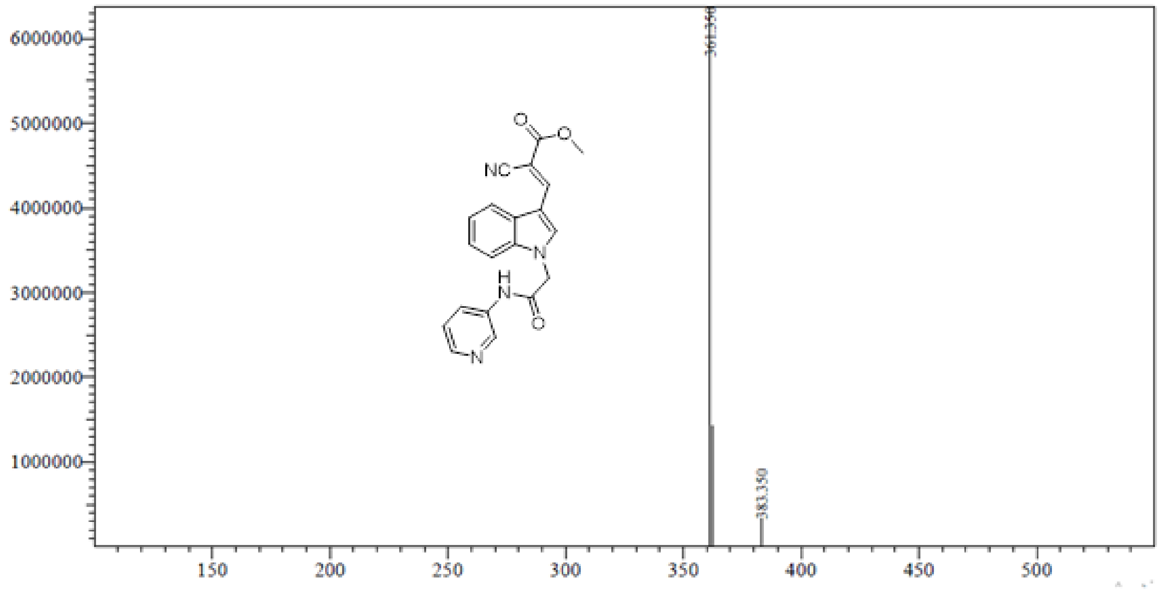

Figure S63 MS spectrum of compound **32**.

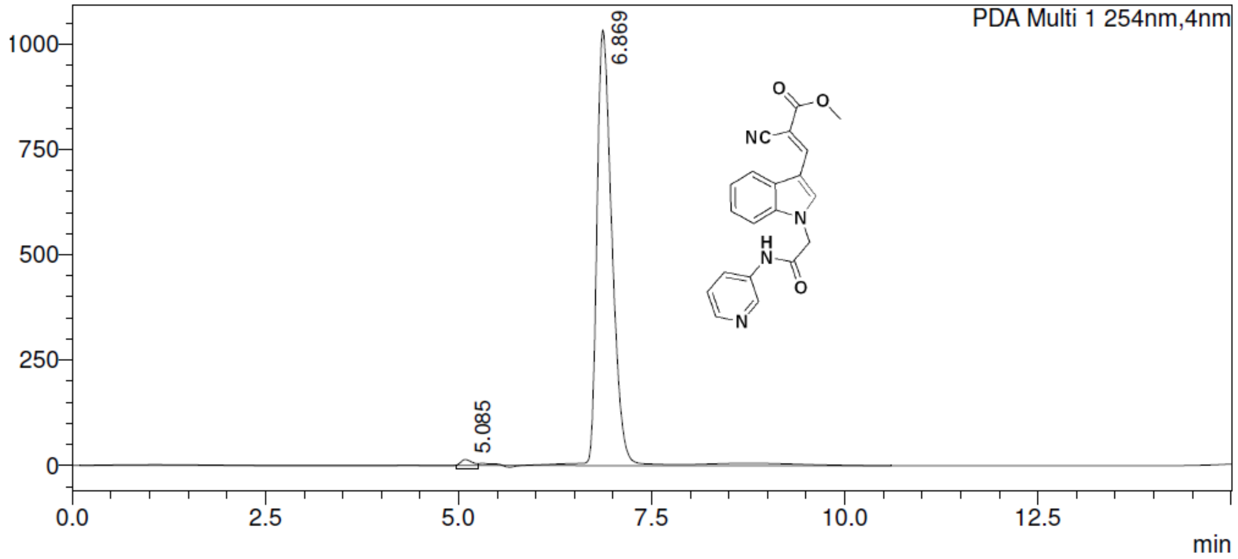

PDA Ch1 254nm

Peak#	Ret. Time	Area	Height	Area%	Height%
1	5.085	272538	21708	1.819	2.056
2	6.869	14710132	1033983	98.181	97.944
Total		14982670	1055690	100.000	100.000

Figure S64 HPLC chromatogram of compound **32**.
